# Elastin stabilization prevents impaired biomechanics in human pulmonary arteries and pulmonary hypertension in rats with left heart disease

**DOI:** 10.1038/s41467-023-39934-z

**Published:** 2023-07-21

**Authors:** Mariya M. Kucherenko, Pengchao Sang, Juquan Yao, Tara Gransar, Saphala Dhital, Jana Grune, Szandor Simmons, Laura Michalick, Dag Wulsten, Mario Thiele, Orr Shomroni, Felix Hennig, Ruhi Yeter, Natalia Solowjowa, Gabriela Salinas, Georg N. Duda, Volkmar Falk, Naren R. Vyavahare, Wolfgang M. Kuebler, Christoph Knosalla

**Affiliations:** 1 Department of Cardiothoracic and Vascular Surgery, Deutsches Herzzentrum der Charité (DHZC), Augustenburger Platz 1, 13353 Berlin, Germany; 2 Charité-Universitätsmedizin Berlin, corporate member of Freie Universität Berlin and Humboldt-Universität zu Berlin, Germany, Charitéplatz 1, 10117 Berlin, Germany; 3 Institute of Physiology, Charité-Universitätsmedizin Berlin, corporate member of Freie Universität Berlin and Humboldt-Universität zu Berlin, Charitéplatz 1, 10117 Berlin, Germany; 4 DZHK (German Centre for Cardiovascular Research), partner site Berlin, Germany; 5 Department of Bioengineering, Clemson University, 29634 Clemson, SC USA; 6 Julius Wolff Institute for Biomechanics and Musculoskeletal Regeneration, Berlin Institute of Health at Charité - Universitätsmedizin Berlin, Augustenburger Platz 1, 13353 Berlin, Germany; 7 NGS Integrative Genomics (NIG), Justus-von-Liebig-Weg 11, 37077 Göttingen, Germany; 8 Berlin Institute of Health Center for Regenerative Therapies, Berlin Institute of Health at Charité - Universitätsmedizin Berlin, Augustenburger Platz 1, 13353 Berlin, Germany; 9 Department of Health Science and Technology, Translational Cardiovascular Technology, LFW C 13.2, ETH Zurich, Universitätstrasse 2, 8092 Zürich, Switzerland; 10 Departments of Physiology and Surgery, University of Toronto, 1 King´s College Circle, Toronto, ON M5S 1A8 Canada

**Keywords:** Mechanisms of disease, Molecular medicine, Hypertension, Translational research, Experimental models of disease

## Abstract

Pulmonary hypertension worsens outcome in left heart disease. Stiffening of the pulmonary artery may drive this pathology by increasing right ventricular dysfunction and lung vascular remodeling. Here we show increased stiffness of pulmonary arteries from patients with left heart disease that correlates with impaired pulmonary hemodynamics. Extracellular matrix remodeling in the pulmonary arterial wall, manifested by dysregulated genes implicated in elastin degradation, precedes the onset of pulmonary hypertension. The resulting degradation of elastic fibers is paralleled by an accumulation of fibrillar collagens. Pentagalloyl glucose preserves arterial elastic fibers from elastolysis, reduces inflammation and collagen accumulation, improves pulmonary artery biomechanics, and normalizes right ventricular and pulmonary hemodynamics in a rat model of pulmonary hypertension due to left heart disease. Thus, targeting extracellular matrix remodeling may present a therapeutic approach for pulmonary hypertension due to left heart disease.

## Abstract (web summary)

Pulmonary hypertension due to left heart disease is characterized by pulmonary arterial stiffening that results from extracellular matrix remodeling. Here, the authors show that elastin stabilization improves arterial biomechanics and attenuates pulmonary hypertension.

## Introduction

The passive mechanics of conduit arteries are determined by two major extracellular matrix (ECM) components – elastic and collagen fibers. In the medial layer of the arterial wall, multiprotein elastic fibers are organized into interconnected fenestrated lamellae anchored to smooth muscle cells (SMCs) and surrounded by collagen fibers. These elastic fibers allow arteries to extend and recoil, while collagen yields structural strength and limits vessel distension. In addition, elastic fibers inhibit the proliferation and migration of SMCs and instead induce and maintain their contractile phenotype^1–3^. In unloaded healthy arteries, both elastic and collagen fibers appear tortuous. Under physiological vascular pressures, most elastic lamellae become uncoiled, while only 10% of collagen fibers straighten and bear the load^4–6^. With further increases in vascular pressure, the amount of load-bearing collagen fibers increases and limits arterial distension.

Vascular disease in both the systemic circulation and the pulmonary circulation is frequently associated with altered biomechanical properties of blood vessels, which can be attributed to changes in the ratio of collagen fibers to elastin fibers and/or the crosslinking of fibers^7,8^. Even though elastin is one of the most stable ECM proteins^9^, it can be proteolytically degraded by serine and cysteine proteases (such as neutrophil and pancreatic elastases and trypsin or cathepsins, respectively) and members of the matrix metalloprotease (MMP) family. Elastic fiber fragmentation and degradation may similarly result from the downregulation of tissue inhibitors of MMPs (e.g. TIMPs)^10^. In addition to elastolysis, ECM stiffness may further increase in pathological conditions via extensive collagen expression and deposition or enzymatic or non-enzymatic cross-linking of fibers by lysyl oxidases (LOXs) or advanced glycation end-products (AGEs), respectively^7,8,10–13^. As a result of these processes, arterial biomechanical competences progressively deteriorate, accelerating vascular disease.

Pulmonary artery (PA) stiffening has emerged as a marker and predictor of disease severity and poor functional status in pulmonary arterial hypertension (PAH, World Health Organization Group I PH)^14–17^. In a rat model of hypoxic PH, PA remodeling and gene expression were found to be associated with changes in PA zero-stress state and rheology^18,19^. In monocrotaline-induced pulmonary hypertension (PH), disease progression is associated with increased collagen synthesis and a decreased proportion of elastin in the PA media^20^, while transgenic S100A4/Mts1 mice that occasionally develop PH show upregulated expression of fibulin-5, an ECM component that controls elastic fiber assembly^21^. The biosynthesis of elastin and fibrillin-1, two major constituents of elastic fibers, is negatively regulated by increased TGF-β and reduced BMPR2 signaling, i.e., classic drivers of PH and lung vascular remodeling^22^. Accordingly, elastic fibers in the PA wall of patients with PAH, especially in BMPR2 mutation carriers, are reduced^22^. Importantly, progressive PA stiffening may in turn promote right ventricular (RV) dysfunction and lung vascular injury and remodeling, thus driving PH pathology in a positive feed-forward loop. Specifically, stiffening of conduit PAs increases pulse-wave velocity, further augmenting pulmonary arterial pressure (PAP) and RV afterload, while increased pulse pressure amplitude in distal vessels promotes microvascular injury and hence, remodeling^23,24^. Consequently, the stiffening of proximal conduit PAs as detected by intravascular ultrasound and cardiac magnetic resonance imaging (MRI) has recently emerged as a sensitive biomarker and risk predictor in PAH^25,26^.

In contrast to PAH, which is a rare disease, PH caused by left heart disease (PH-LHD, World Health Organization Group II PH) is poised to become a global pandemic affecting approximately 1% of the global population. The prevalence even increases up to 10% in individuals over 65 years old^27^. Pathologically, PH-LHD is caused by passive backward congestion of pressure from the left ventricle (LV) into the pulmonary circulation (isolated postcapillary PH) and subsequent active remodeling in all segments of the pulmonary vasculature^28,29^ (combined post- and precapillary PH). Both pre- and postcapillary PH increase RV afterload and ultimately cause death by progressive RV failure^30–32^. The clinical relevance of PH-LHD is highlighted by the fact that in heart failure patients, the presence of PH accelerates disease progression and increases morbidity and mortality independent of the severity of left ventricular systolic dysfunction or the degree of functional mitral regurgitation^32,33^. PH-LHD patients also comprise a high-risk group for surgical treatment of heart failure with a high chance for impaired outcomes with the implantation of a left ventricular assist device and/or heart transplantation^34^. In PH-LHD, however, changes in PA biomechanical competences and tissue stiffness have thus far not been addressed. This knowledge gap is particularly striking given the high incidence of PH-LHD and its very different pathophysiology relative to PAH. An in-depth analysis of PA stiffening in PH-LHD may thus not only yield a novel biomarker for this disease but also provide a basis for cellular, molecular, and physiological insights that may facilitate improved disease modeling and the development of novel diagnostic tools and therapeutic strategies.

In the present study, we performed a comprehensive mechanobiological analysis of PA samples from patients with left heart disease (LHD). We identified PA stiffening as a characteristic feature of PH-LHD. ECM remodeling precedes the onset of clinical PH and was characterized by progressive fragmentation and degradation of elastin and a parallel increase in AGE-crosslinked fibrillar collagens. In ex vivo cultured human PAs, the stabilization of elastin with the polyphenolic compound pentagalloyl glucose (PGG) reduced elastolysis and improved arterial biomechanical competences. In a rat model of PH-LHD, nanoparticle (NP)-based targeted delivery of PGG reversed PA stiffening, normalized lung vascular homeostasis, and prevented the development of PH, thus pointing toward a core role of ECM remodeling in PH and accordingly, to ECM as a promising therapeutic target in LHD patients.

## Results

### Conduit PAs are stiffened in PH-LHD

**Fig. 1:**
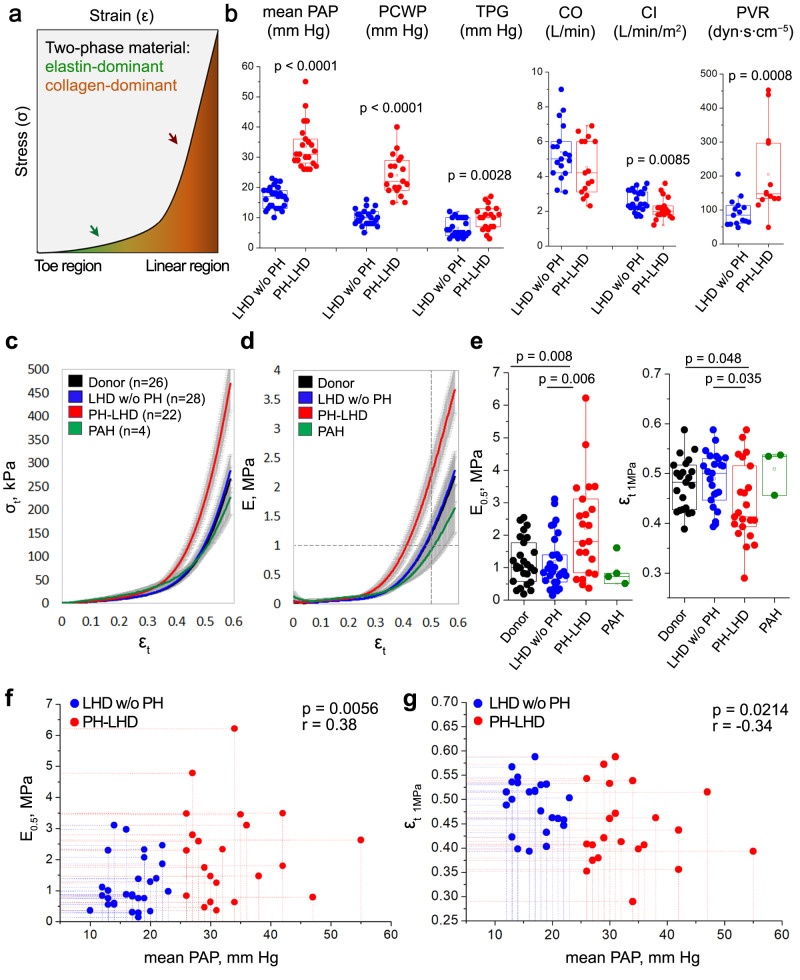
Conduit pulmonary arteries are stiffened in PH-LHD. **a** Schematic illustration depicting the typical stress/strain curve of a conduit artery undergoing a uniaxial tensile test. The toe region (green) reflects the tensile properties of primarily elastin-enriched material, while the linear region (brown) is dominated by collagen-enriched material. **b** Box-and-whisker plots show pulmonary hemodynamics and cardiac function in patients with LHD w/o PH or PH-LHD. Groups differ in mean pulmonary arterial pressure (mean PAP, *p* < 0.0001, LHD w/o PH *n* = 28, PH-LHD *n* = 22 patients), pulmonary capillary wedge pressure (PCWP, *p* < 0.0001, LHD w/o PH *n* = 23, PH-LHD *n* = 19 patients), transpulmonary gradient (TPG, *p* = 0.0028, LHD w/o PH *n* = 23, PH-LHD *n* = 19 patients), pulmonary vascular resistance (PVR, *p* = 0.0008, LHD w/o PH *n* = 15, PH-LHD *n* = 13 patients), and cardiac index (CI, *p* = 0.0085, LHD w/o PH *n* = 18, PH-LHD *n* = 15 patients). **c** Group data show σ_t_/ε_t_ curves (mean ± SEM) for biologically independent PA samples from donors (*n* = 26), LHD w/o PH (*n* = 28), PH-LHD (*n* = 22), or PAH (*n* = 4) patients. **d** Group data show corresponding E/ε_t_ curves (mean ± SEM) for biologically independent PA samples from donors (*n* = 26), LHD w/o PH patients (*n* = 28), PH-LHD patients (*n* = 22), or PAH patients (*n* = 4). Dashed lines indicate 0.5 ε_t_ for which individual stiffness (E_0.5_) was derived, and 1 MPa stiffness for which corresponding individual true strain (ε_t 1MPa_) is reported. **e** Box-and-whisker plots show E_0.5_ in biologically independent PA samples from donors (*n* = 26), LHD w/o PH (*n* = 28), PH-LHD (*n* = 22), or PAH (*n* = 4) patients (*p* = 0.008 for PH-LHD vs. donor and *p* = 0.006 for PH-LHD vs. LHD w/o PH) and ε_t 1MPa_ in biologically independent PA samples from donors (*n* = 22), LHD w/o PH (*n* = 24), PH-LHD (*n* = 22), and PAH (*n* = 4) patients (*p* = 0.048 for PH-LHD vs. donor and *p* = 0.035 for PH-LHD vs. LHD w/o PH). **f**–**g** Scatter diagrams show the relationship between mean PAP and parameters of pulmonary arterial biomechanics, namely, E_0.5_ (*n* = 50 patients/biologically independent PA samples, r = 0.38, *p* = 0.0056, 95% CI = 0.1124 to 0.6053) and ε_t 1MPa_ (*n* = 46 patients/biologically independent PA samples, r = −0.34, *p* = 0.0214, 95% CI = −0.5784 to 0.04450). Box-and-whisker plots overlaid with dot plots (**b**, **e**) show individual data points, mean (rectangle), median (line within the box), lower and upper 25% quartiles (limits of the box), 1.5 IQR (whiskers), and outliers (if applicable). *Statistics*: **b**, **e** Unpaired two-tailed Mann–Whitney U-test; **f**–**g** Spearman’s coefficient of correlation Rho (r) and corresponding two-tailed statistics. Source data are provided as a Source Data file.

To compare PA biomechanical properties between LHD and healthy-heart controls (donor hearts), circumferential uniaxial tensile tests were performed ex vivo on conduit PA samples obtained from donors and recipients during cardiac transplantation (Suppl. Fig. 1a, b, for the underlying disease of the LHD cohort, see Methods). Since arterial mechanics are primarily determined by elastic and collagen fibers, the arterial wall can be structurally viewed as a two-phase material^35^. Accordingly, arterial stress/strain curves sequentially reveal a low-energy toe region and a high-energy linear region reflecting the biomechanical properties of the elastin- and collagen-dominated material, respectively (Fig. 1a)^35,36^. PA force/displacement curves revealed characteristic differential patterns between LHD and control samples (Suppl. Fig. 1c). Next, we differentiated PH-LHD patients with a mean PAP ≥ 25 mm Hg and a pulmonary capillary wedge pressure (PCWP) ≥ 15 mm Hg from LHD patients w/o PH. Groups were matched by age and sex distribution (Methods). In comparison to LHD w/o PH patients, the PH-LHD cohort had a significantly increased transpulmonary gradient (TPG) and pulmonary vascular resistance (PVR) and a reduced cardiac index (CI) (Fig. 1b). In line with previous reports^37^, PA ectasia was evident in PH-LHD patients by computed tomography (CT) as increased PA trunk diameter (Suppl. Fig. 2). Conversion of force/displacement to true stress/strain (σ_t_/ε_t_) curves (see Methods) revealed steeper averaged σ_t_/ε_t_ curves in PH-LHD patients as compared to healthy-heart donors or LHD w/o PH subjects, respectively (Fig. 1c). A similar difference between these groups was evident in stiffness (E, instantaneous Young’s modulus derived from stress as function of strain) over ε_t_ curves (Fig. 1d). Quantitative analyses of E/ε_t_ curves showed higher PA stiffness at 0.5 strain (E_0.5_) in samples from PH-LHD patients as compared to healthy-heart donors or LHD w/o PH patients, while true strain required to generate 1 MPa stiffness (ε_t 1MPa_) was significantly lower (Fig. 1e), together demonstrating PA stiffening in PH-LHD.

Spearman analyses yielded significant correlations between PA biomechanical properties and pulmonary hemodynamics for mean PAP (Fig. 1f-g) and PCWP (Suppl. Table 1), consolidating the close association between PA stiffness and pulmonary hemodynamics in PH-LHD. In consideration of the recent reduction of the cut-off value for the diagnosis of chronic PH from ≥ 25 to > 20 mmHg at rest^38^, we specifically evaluated PA biomechanical competences in LHD patients with a mean PAP in the range of 20-25 mmHg (*n* = 4). No significant differences in E_0.5_ or ε_t 1MPa_ were detectable between these patients, healthy-heart donors, or LHD patients with a mean PAP ≤ 20 mmHg (Suppl. Fig. 1d, e), yet this result should be interpreted with caution in view of the low sample size of this patient subgroup. We also found no evidence that PA stiffening in PH-LHD is related to diabetes mellitus as a comorbidity (Suppl. Fig. 1e). However, in addition to correlations with pulmonary hemodynamics, PA stiffening was more pronounced with age and more frequent in male patients (Suppl. Fig. 3). In line with previous studies reporting beneficial effects of estrogen on PA biomechanics in a rat model of PH^39,40^, these sex differences (which yet did not reach the level of significance in our study) may indicate an increased sensitivity to develop PA stiffening in PH-LHD males relative to females.

Conduit PA samples from PAH patients yielded stress/strain curves that resembled the patterns in donor and LHD w/o PH subjects but not that in PH-LHD patients (Fig. 1c, d and Suppl. Fig. 1c’). Analogously, E_0.5_ and ε_t 1MPa_ values in PAH samples were largely similar to those in control samples (Fig. 1e). These results indicate that stiffening of the conduit PA is distinctly more pronounced in PH-LHD patients than in PAH patients.

### Transcriptional regulation of PA stiffening

**Fig. 2:**
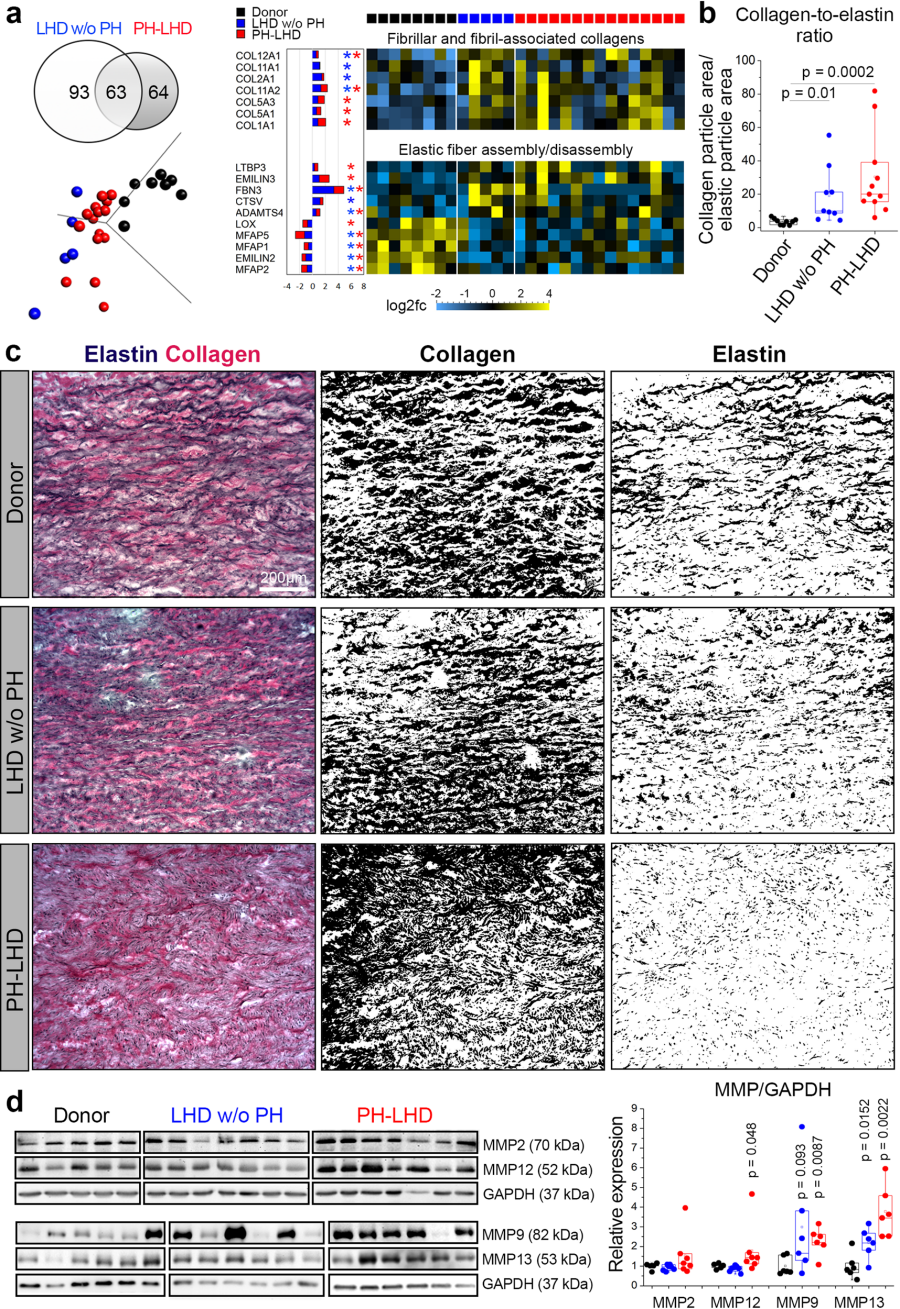
Transcriptional regulation of pulmonary artery stiffening. **a** Venn diagram showing the numbers of genes involved in ECM remodeling that are differentially expressed in PA samples of LHD w/o PH patients vs. PH-LHD patients. A principal component analysis (PCA) plot depicts sample clustering based on ECM gene expression profiles. Heat maps display log2-fold changes in the expression of fibrillar and fibril-associated collagens and genes involved in elastic fiber assembly/disassembly in PA samples of LHD w/o PH and PH-LHD patients relative to donor samples. **b** Box-and-whisker plot showing the collagen-to-elastin ratio in biologically independent PA samples from donors (*n* = 9), LHD w/o PH (*n* = 9), or PH-LHD (*n* = 11) patients (*p* = 0.01 for LHD w/o PH vs. donor, and *p* = 0.0002 for PH-LHD vs. donor). **c** Representative images of EVG-stained PA medial walls from a donor, an LHD w/o PH patient, and a PH-LHD patient (left panels). The center panels show collagen-positive area only, right panels show elastic fibers only. Notably, the combination of hematoxylin with EVG also stains cell nuclei, which are hence included in the elastin stain signal on the right panel. The presented images are derived from biologically independent PA samples from donors (*n* = 9), LHD w/o PH (*n* = 9), and PH-LHD (*n* = 11) patients. **d** Immunoblots show protein levels of MMP2 (70 kDa), MMP12 (52 kDa), and GAPDH (loading control, 37 kDa) in biologically independent PA samples from donors (*n* = 5), LHD w/o PH (*n* = 7), and PH-LHD (*n* = 7) patients and MMP9 (82 kDa), MMP13 (53 kDa), and GAPDH (loading control, 37 kDa) in biologically independent PA samples from donors (*n* = 6), LHD w/o PH (*n* = 6), and PH-LHD (*n* = 6) patients. Box-and-whisker plots show quantitative densitometric data of MMP expression normalized to GAPDH and the mean of the donor controls. MMP expression differs between groups as follows: MMP12 (*p* = 0.048 for PH-LHD vs. donor), MMP9 (*p* = 0.093 for LHD w/o PH vs. donor and *p* = 0.0087 for PH-LHD vs. donor), MMP13 (*p* = 0.0152 for LHD w/o PH vs. donor and *p* = 0.0022 for PH-LHD vs. donor). Box-and-whisker plots overlaid with dot plots (**b**, **d**) show individual data points, mean (rectangle), median (line within the box), lower and upper 25% quartiles (limits of the box), 1.5 IQR (whiskers), and outliers (if applicable). *Statistics*: **b** Unpaired two-tailed Kruskal-Wallis one-way ANOVA on ranks followed by pairwise multiple comparisons (Dunn’s test). **d** Unpaired two-tailed Mann–Whitney U test (vs. donor). Source data are provided as a Source Data file. Full scan blots are provided in Suppl. Fig. 12.

To obtain insights into the mechanisms that drive PA stiffening in PH-LHD, we performed transcriptomic analyses of ECM structural constituents and their turnover in human PA samples. Although increased PA stiffness was a specific hallmark of PH-LHD patients, transcriptomic analyses revealed robust dysregulation of genes associated with ECM formation or remodeling not only in PH-LHD samples but also in samples from LHD w/o PH patients. Out of 220 dysregulated candidate genes, 156 genes were differentially expressed in LHD w/o PH samples, and 127 genes were differentially expressed in PH-LHD samples compared to healthy donors (Fig. 2a). Principal component analysis (PCA) of matrisome gene expression revealed the clustering of LHD w/o PH samples in close proximity to PH-LHD samples and apart from donor samples (Fig. 2a). This finding suggests a substantial dysregulation of ECM genes in the PA wall of LHD patients that precedes the development of overt PA stiffening and PH. Importantly, a sizeable number of differentially regulated ECM genes were shared between the LHD w/o PH and PH-LHD groups (Suppl. Figs. 4–6), indicating that transcriptional dysregulation of the PA matrisome presents (at least in part) a continuous process that initiates at the stage of LHD and continues throughout the progression to PH.

To gain mechanistic insight into the process of PA stiffening, we next focused our transcriptomic analyses on genes regulating elastic and collagen fibers (Fig. 2a). Prominent among the genes upregulated in LHD w/o PH and/or PH-LHD samples were those coding for subunits of fibrillar collagen types I, II, V, and XI and fibril-associated collagen XII. Specifically, subunits of fibrillar collagen II and XI were upregulated in PAs in LHD w/o PH, while PH-LHD PAs showed increased expression of collagen I and V, a finding that is notable as the upregulation of collagen I has been implicated with vascular stiffening in hypertension and aging^41,42^ and since collagen I fragments are increased in the blood of PAH patients^43^.

In contrast to the upregulation of collagen subunits, several genes involved in elastic fiber assembly, regulation, and anchoring, including microfibrillar-associated proteins (MFAP) 1, 2, and 5, elastin microfibril interface-located protein 2 (EMILIN2), and the crosslinking enzyme lysine-6-oxidase (LOX), were downregulated in both LHD w/o PH and PH-LHD samples. Conversely, the expression of the elastic fiber-degrading enzymes cysteine cathepsin V (CTSV) and disintegrin and metalloproteinase with thrombospondin motifs-4 (ADAMTS4)^44^ was increased in PH-LHD samples, as were two proteins, transforming growth factor beta-binding protein 3 (LTBP3) and EMILIN3, that localize on elastic fibers to antagonize transforming growth factor beta (TGF-β) signaling^45–47^.

Combined, our biomechanical and matrisome analyses identify extensive remodeling of elastic and collagen matrices in the PA wall as a likely cause of vascular stiffening in PH-LHD. To explore this hypothesis, we next visualized elastic and collagen fibers on histological PA sections by Verhoeff’s elastin Van Gieson (EVG) staining. Relative to healthy-heart controls, extensive ECM remodeling was evident in LHD w/o PH samples but was even more pronounced in PH-LHD samples (Fig. 2b-c). While collagen staining was increased, elastic fiber staining was decreased in LHD w/o PH samples and even more so in PH-LHD samples, resulting in a progressive increase in the collagen-to-elastin ratio.

Quantification of protein expression for several matrix metalloproteinases (MMPs) previously implicated in the degradation of arterial elastic and collagen fibers, vascular stiffening^12,48–51^ and the development of PAH^52^ revealed increased expression of the gelatinase MMP9, the macrophage elastase MMP12, and the interstitial collagenase MMP13 in PAs of PH-LHD patients, but importantly also in part in LHD w/o PH patients with MMP9 just missing (*p* = 0.093) and MMP13 reaching the level of significance (Fig. 2d). Intriguingly, the latter finding indicates that enzymatic dysregulation of ECM turnover precedes PA stiffening and clinical PH in PH-LHD.

### Progressive fragmentation and degradation of elastic fibers

**Fig. 3:**
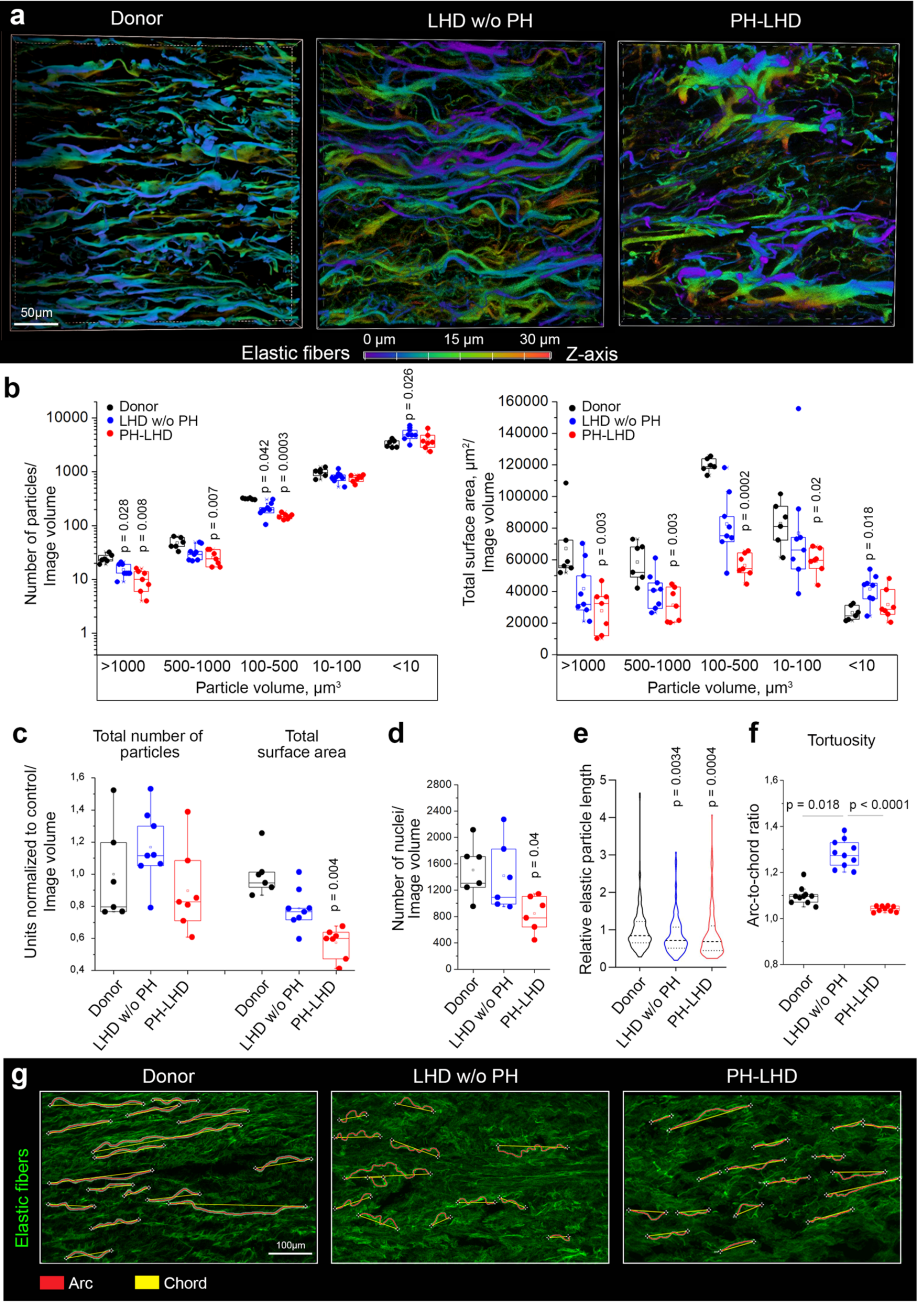
Progressive fragmentation and degradation of elastic fibers. **a** Representative images show elastic fibers in arterial wall media as detected by autofluorescence in the PA of a donor, an LHD w/o PH patient, and a PH-LHD patient. Pseudocolors reflect the vertical depth of fibers in the z-axis (scale). **b** Box-and-whisker plots show the number and surface area of elastic particles classified by volume in biologically independent PA samples from donors (*n* = 6), LHD w/o PH (*n* = 8), and PH-LHD (*n* = 7) patients. The corresponding p-values for statistical comparisons between LHD w/o PH or PH-LHD groups vs. donors are given. **c** Box-and-whisker plots show the total number and total surface area of elastic fibers in biologically independent PA samples from donors (*n* = 6), LHD w/o PH (*n* = 8), and PH-LHD (*n* = 7) patients normalized to donor control (*p* = 0.004 for PH-LHD vs. donor). **d** Box-and-whisker plot shows numbers of nuclei as quantified by DRAQ5 staining in biologically independent PA samples from donors (*n* = 6), LHD w/o PH (*n* = 6) and PH-LHD (*n* = 6) patients (*p* = 0.04 for PH-LHD vs. donor). **e** Violine plot shows the distribution of elastic particle lengths in PA samples from donors (*n* = 195 particles examined in 4 biologically independent samples), LHD w/o PH (*n* = 143 particles examined in 3 biologically independent samples), and PH-LHD (*n* = 133 particles examined in 3 biologically independent samples) patients (*p* = 0.0034 for LHD w/o PH vs. donor and *p* = 0.0004 for PH-LHD vs. donor). **f** Box-and-whisker plot showing elastic fiber tortuosity measured as the arc-to-chord ratio in biologically independent PA samples from donors (*n* = 10), LHD w/o PH (*n* = 10) and PH-LHD (*n* = 9) patients (*p* = 0.018 for LHD w/o PH vs. donor, and *p* < 0.0001 for PH-LHD vs. LHD w/o PH). **g** Single-plane confocal images show elastic fibers as detected by autofluorescence with corresponding arc (red) and chord (yellow) lengths. Images are derived from biologically independent PA samples from donors (*n* = 10), LHD w/o PH (*n* = 10), and PH-LHD (*n* = 9) patients. Box-and-whisker plots overlaid with dot plots (**b**–**d**, **f**) show individual data points, mean (rectangle), median (line within the box), lower and upper 25% quartiles (limits of the box), 1.5 IQR (whiskers), and outliers (if applicable); violine plot (**e**) shows median (dashed line) and lower and upper 25% quartiles (dotted lines).*Statistics*: **b**–**f** Unpaired two-tailed Kruskal-Wallis one-way ANOVA on ranks followed by pairwise comparisons vs. donor. Source data are provided as a Source Data file.

To further explore structural changes in the elastic lamellae, we next imaged the medial layer of the PA wall by confocal microscopy followed by 3D network reconstruction (Fig. 3a). In comparison to both donor and LHD w/o PH samples, elastic fibers in the PAs of PH-LHD patients were fewer and arranged in a more random pattern (Fig. 3a and Suppl. Videos 1–3). Volumetric classification of elastic particles revealed that the total number of particles, as well as the particle surface area, were significantly reduced for all particle sizes in PH-LHD samples compared to healthy donor samples, while small ( < 10 µm^3^) particles were increased in the PAs of LHD w/o PH patients (Fig. 3b). The latter finding was associated with a distinct decrease in all larger particle classes ( > 10 µm³); however, the difference failed to reach significance for each individual class. That notwithstanding, the loss of larger particles and the accumulation of smaller particles indicate that LHD w/o PH is likely associated with the progressive fragmentation of elastic fibers. Compared to the values in LHD w/o PH samples, the total number and surface area of overall particles were decreased in PH-LHD samples (Fig. 3c), suggesting additional degradation of elastic fibers. Transmission electron microscopy (TEM) of the PA media revealed thinner elastic fibers with delaminated fragments in LHD w/o PH samples compared to control samples, while elastic fibers in PH-LHD samples were largely deteriorated and fragmented (Suppl. Fig. 7a, blue arrows). In both LHD w/o PH and PH-LHD samples, the elastin core of the elastic fibers was substantially degraded (Suppl. Fig. 7b, pink arrows), although the expression levels of α-elastin or fibrillin-1 were unchanged (Suppl. Fig. 7c). Considering that elastic fiber fragmentation may enhance the release of TGF-β^53^, a key autocrine driver of lung vascular remodeling^54^ that is bound to fibrillin through the latent TGF-β binding protein (LTBP) – latency-associated peptide (LAP) complex^55^, we probed plasma samples of LHD patients for mature TGF-β. In plasma of LHD w/o PH patients, levels of monomeric and dimeric TGF-β were comparable to healthy controls, yet both forms were elevated in PH-LHD patients (Suppl. Fig. 7d–f). Next, we probed for potential interdependencies between plasma TGF-β levels and pulmonary hemodynamics or PA biomechanics (Suppl. Fig. 7g), respectively. Yet, Spearman correlation analyses revealed no significant correlations between plasma TGF-β levels and either mean PAP or PA maximal stress (Suppl. Fig. 7h). On the one hand, this does not preclude an important role of TGF-β in PA remodeling as plasma TGF-β levels do not adequately reflect local cytokine concentrations. On the other hand, these findings do not support a direct effect of PA elastolysis on systemic TGF-β bioavailability in LHD patients, potentially because PA stiffening also negatively regulates vascular cell contraction which is required for the activation of latent TGF-β^56^.

Since elastic fibers form a physiological niche that maintains functional SMCs, we next quantified the cell number per image volume by DRAQ5 staining of histological sections. In line with a progressive dysregulation of the elastic fiber micromilieu, significantly fewer cells were detected in PH-LHD samples than in control samples (Fig. 3d). TEM imaging revealed that in healthy PAs, a substantial SMC surface interacts with the elastic fibers (Suppl. Fig. 7b, green arrows). In both the LHD w/o PH and PH-LHD arteries, SMC-elastic fiber connections were, in contrast, markedly reduced, suggesting impaired SMC anchoring to remodeled elastic fibers (Suppl. Fig. 7b).

In addition to elastic fiber fragmentation, residual elastic fibers in LHD w/o PH samples appeared crimped, i.e., shorter albeit of increased tortuosity (Suppl. Videos 1–3 and Suppl. Fig. 7a). In contrast, fibers in PH-LHD samples were similarly shorter but again straightened (Fig. 3e–g). As we will discuss below, this finding may indicate increased fragmentation of elastic lamellae in LHD w/o PH, resulting in fiber retraction, which in an unloaded artery will appear as hypercrimping. In contrast, straightened elastic fibers in PH-LHD PAs fail to retract upon unloading, indicating reduced recoil of the pathologically remodeled fiber network.

### Increased deposition of fibrillar collagen

**Fig. 4:**
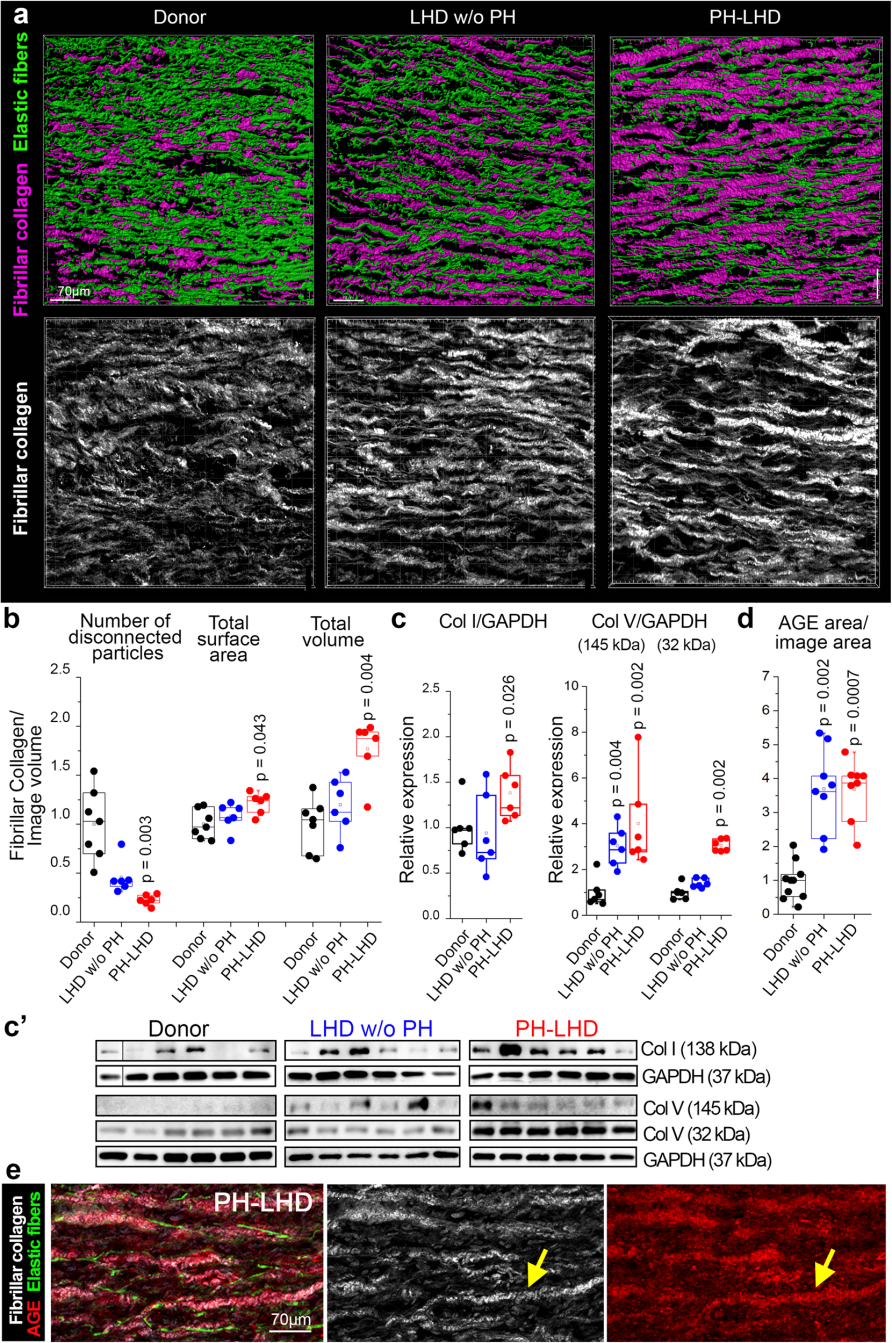
Increased deposition of fibrillar collagen. **a** Representative images show (upper panel) exemplary 3D reconstruction of fibrillar collagen and (lower panel) volume reconstructions of both fibrillar collagen and elastic fiber networks in the arterial media as detected by SHG and autofluorescence, respectively, in the PA of a donor, an LHD w/o PH patient, and a PH-LHD patient. **b** Box-and-whisker plots show the number of disconnected collagen particles, total surface area, and total volume of fibrillar collagen normalized to control and to image volume in the PA of a donor, an LHD w/o PH patient, and a PH-LHD patient. **c**–**c**’ Immunoblots show protein levels of Col I (138 kDa), full length (145 kDa), and C-terminal truncation product (32 kDa) of Col V, and loading control GAPDH (37 kDa) in biologically independent PA samples from donors (*n* = 6), LHD w/o PH (*n* = 6), and PH-LHD (*n* = 6) patients. Box-and-whisker plots show quantitative densitometric data of Col I and Col V expression (normalized to GAPDH and to the mean of the donor controls) in biologically independent PA samples from donors (*n* = 6), LHD w/o PH (*n* = 6), and PH-LHD (*n* = 6) patients. Collagen expression differs between groups as follows: Col I (*p* = 0.026 for PH-LHD vs. donor), Col V 145 kDa (*p* = 0.004 for LHD w/o PH vs. donor, and *p* = 0.002 for PH-LHD vs. donor), and Col V 32 kDa (*p* = 0.002 for PH-LHD vs. donor). **d** Box-and-whisker plots show area of anti-AGE immunostaining normalized to area of the image and to donor controls in biologically independent PA samples from donors (*n* = 10), LHD w/o PH (*n* = 8), and PH-LHD (*n* = 8) patients (*p* = 0.0014 for LHD w/o PH vs. donor and *p* = 0.0004 for PH-LHD vs. donor). **e** Representative images of the arterial media in a PA sample from a PH-LHD patient show co-localization of anti-AGE immunostaining (right) with fibrillar collagen (center). Similar co-localization was confirmed in biologically independent PA samples from *n* = 11 LHD w/o PH or PH-LHD patients. Box-and-whisker plots overlaid with dot plots (**b**–**d**) show individual data points, mean (rectangle), median (line within the box), lower and upper 25% quartiles (limits of the box), 1.5 IQR (whiskers), and outliers (if applicable). *Statistics*: **b**, **d** Unpaired two-tailed Kruskal–Wallis one-way ANOVA on ranks followed by pairwise multiple comparisons (Dunn’s test); **c** Unpaired two-tailed Mann–Whitney U test (vs. donor). Source data are provided as a Source Data file. Full scan blots are provided in Suppl. Fig. 12.

Next, we observed changes in fibrillar collagen in conjunction with elastic fibers by second-harmonic generation (SHG) imaging (Fig. 4a). In donor PA samples, fibrillar collagen was loosely packed and interwoven by a dense network of elastic fibers. In comparison, fibrillar collagen in LHD w/o PH samples appeared more condensed and elongated but was still embedded in a mesh of multiple tortuous elastic fibers. In PH-LHD samples, however, fibrillar collagen formed long massive rods that were separated only by residual fragments of straightened elastic fibers, suggesting significant deposition of fibrillar collagen. Consistent with this notion, quantitative image analysis revealed a reduced number of disconnected particles, an increase in surface area, and a marked increase in the total volume of fibrillar collagen in PH-LHD PAs compared to donor PAs (Fig. 4b). Ultrastructural analysis showed that in comparison to control PAs, collagen fibrils were more densely packed into collagen fibers in LHD w/o PH samples and even more so in PH-LHD arteries (Suppl. Fig. 8a, red arrows). Western blot analyses revealed that the increase in fibrillar collagen was associated with the upregulation of both collagen I and collagen V (Fig. 4c–c´), the latter controlling fibrillogenesis of type I collagen^57^ but also forming collagen fibers on its own. Notably, collagen V was increased as full-length pro-collagen in samples of both LHD w/o PH and PH-LHD patients, yet its cleaved C-terminal peptide that indicates the formation of collagen V fibrils was only elevated in PH-LHD samples (Fig. 4c–c’).

Previously, enzymatic crosslinking of collagen I by LOXs was proposed as a pathomechanism of PA stiffening in PAH^58^. However, this mechanism seems less relevant in PH-LHD, as matrisome analyses detected reduced LOX gene expression in the PAs of PH-LHD patients, a finding that was also confirmed at the protein level (Suppl. Fig. 8b–e). Alternatively, collagen crosslinking may be caused by a nonenzymatic reaction between sugar and free amino residues, resulting in the formation of AGEs, which have been shown to promote vascular stiffening^59–61^. While immunostaining detected only traces of AGEs in donor PAs, samples from LHD w/o PH or PH-LHD patients showed significantly higher AGE levels in the arterial wall (Fig. 4d and Suppl. Fig. 8f). SHG imaging revealed that AGEs colocalized with fibrillar collagens (Fig. 4e), suggesting collagen crosslinking by AGEs in PH-LHD.

### Elastin stabilization improves human PA biomechanics ex vivo

Since elastic fiber fragmentation in conduit PAs emerged as an early event in LHD that preceded changes in pulmonary hemodynamics and vascular biomechanics, therapeutic targeting of arterial elastin may present a promising modality to prevent PA stiffening and alleviate reactive PH. PGG is an elastin-stabilizing polyphenolic compound that has been proven beneficial in abdominal aortic aneurysm (AAA) animal models^62–65^. To test whether the stabilization of elastic fibers by PGG may also rescue PA biomechanics, we cultured human PAs ex vivo in the presence or absence of 0.1% PGG and induced elastolysis by incubation with 1 U porcine elastase for 24 h. Elastic fibers were almost completely lost by elastase treatment but partially preserved in the presence of PGG (Suppl. Fig. 9a). Importantly, treatment with PGG improved PA biomechanics, as demonstrated by right shifted σ_t_/ε_t_ and E/ε_t_ curves and increased ε_t 1MPa_ in elastase-treated as well as naïve PAs (Suppl. Fig. 9b–c). As such, PGG may present a promising approach to improve PA biomechanics in pathological scenarios of increased elastolysis, such as PH-LHD.

### Targeted delivery of PGG attenuates PA stiffening and PH in a rat model of PH-LHD

**Fig. 5:**
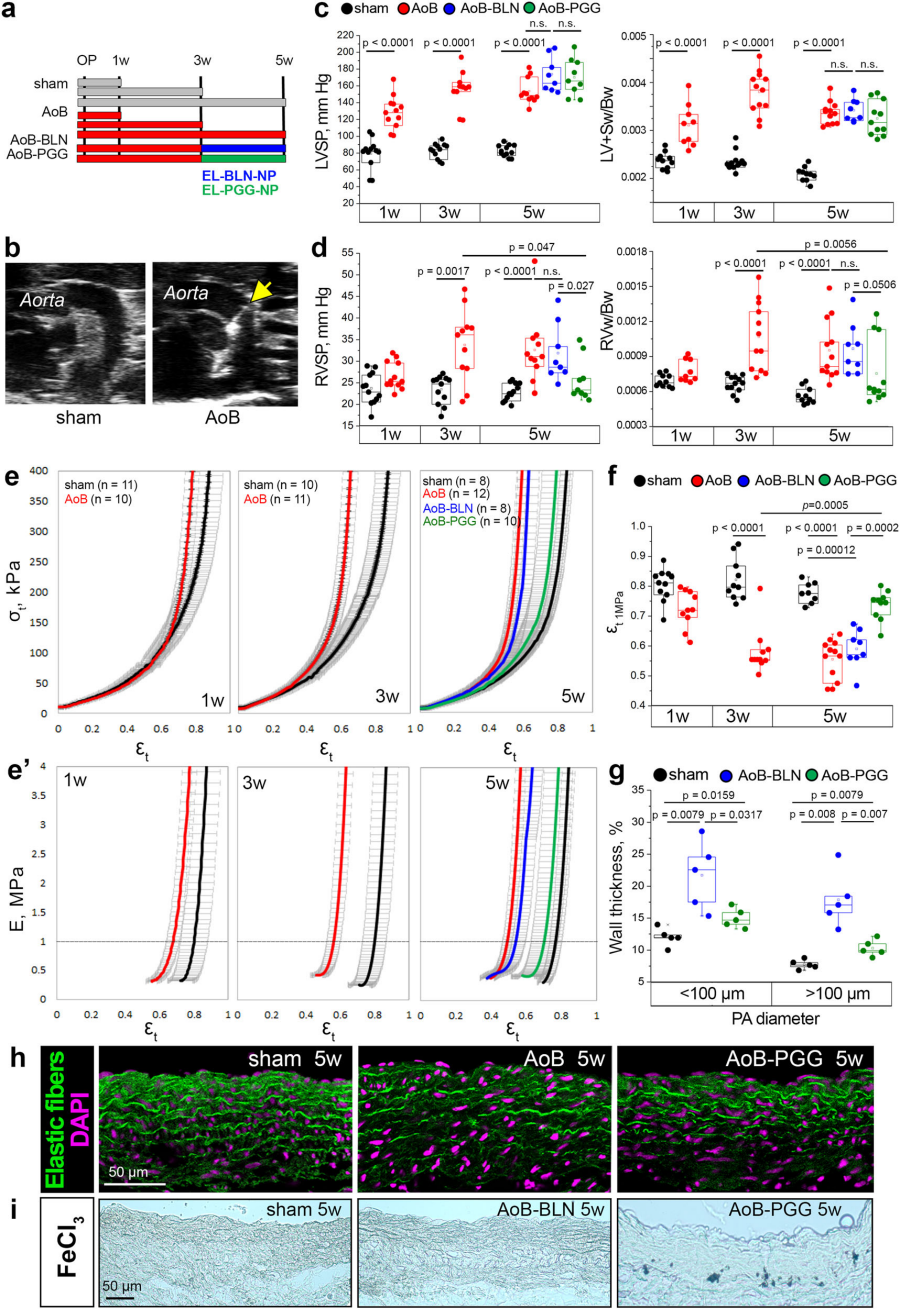
Targeted delivery of PGG attenuates PA stiffening and PH in a rat model of PH-LHD. **a** Schematic depiction of the experimental protocol. Animals underwent sham surgery for AoB with or without PGG treatment and were analyzed after 1 week (1w; sham, AoB), 3 weeks (3w; sham, AoB), or 5 weeks (5w; sham, AoB, AoB-BLN, AoB-PGG). OP, operation (AoB or sham); blue and green bars indicate the time interval of EL-BLN-NP or EL-PGG-NP treatment. **b** Representative echocardiographic images show clip placement (yellow arrow) on the ascending aorta in AoB animals compared to sham rats. **c**, **d** Box-and-whisker plots show left (LVSP) and right (RVSP) ventricular systolic pressures assessed by cardiac catheterization in 1w sham (*n* = 12), 1w AoB (*n* = 12), 3w sham (*n* = 12), 3w AoB (*n* = 11), 5w sham (*n* = 12), 5w AoB (*n* = 11), 5w AoB-BLN (*n* = 8), and 5w AoB-PGG (*n* = 9) animals. Left (LV+Sw/Bw) and right (RVw/Bw) ventricular weights normalized to body weight assessed in 1w sham (*n* = 10), 1w AoB (*n* = 9), 3w sham (*n* = 12), 3w AoB (*n* = 12), 5w sham (*n* = 10), 5w AoB (*n* = 12), 5w AoB-BLN (*n* = 8), and 5w AoB-PGG (*n* = 11) animals. Parameters differ between groups as follows: LVSP and LVw+S/Bw (*p* < 0.0001 for 1w AoB vs. 1w sham, 3w AoB vs. 3w sham, and 5w AoB vs. 5w sham), RVSP (*p* = 0.0017 for 3w AoB vs. 3w sham, *p* < 0.0001 for 5w AoB vs. 5w sham, *p* = 0.027 for 5w AoB-PGG vs. 5w AoB-BLN, and *p* = 0.047 for 5w AoB-PGG vs. 3w AoB), RVw/Bw (*p* < 0.0001 for 3w AoB vs. 3w sham, *p* < 0.0001 for 5w AoB vs. 5w sham, *p* = 0.0506 for 5w AoB-PGG vs. 5w AoB-BLN, and *p* = 0.0056 for 5w AoB-PGG vs. 3w AoB). **e** Group data show σ_t_/ε_t_ curves (mean ± SD) for PAs of AoB and sham animals at 1, 3, and 5 weeks after surgery, and AoB-BLN and AoB-PGG rats after 5 weeks. e’, Group data show corresponding E/ε_t_ (mean ± SD) for PAs of AoB and sham animals at 1, 3, and 5 weeks after surgery, and AoB-BLN and AoB-PGG rats after 5 weeks. Dashed lines indicate 1 MPa stiffness E for which the corresponding strain (ε_t 1MPa_) is reported. **f** Box-and-whisker plots show ε_t 1MPa_ in biologically independent PA samples from 1w sham (*n* = 11), 1w AoB (*n* = 10), 3w sham (*n* = 10), 3w AoB (*n* = 11), 5w sham (*n* = 8), 5w AoB (*n* = 12), 5w AoB-BLN (*n* = 8), and 5w AoB-PGG (*n* = 10) animals (*p* < 0.0001 for 3w AoB vs. 3w sham and 3w AoB vs. 5w sham, *p* = 0.00012 for 5w AoB-BLN vs. 5w sham, *p* = 0.0002 for 5w AoB-PGG vs. 5w AoB-BLN, and *p* = 0.0005 for 5w AoB-PGG vs. 3w AoB). **g** Box-and-whisker plots show PA wall thickness (quantified relative to arterial diameter in %) in the lungs of sham (*n* = 5), AoB-BLN (*n* = 5), and AoB-PGG (*n* = 5) animals. Each dot represents the average value per animal. **h** Representative images show elastic fibers as visualized by autofluorescence in the PAs of sham, AoB, and AoB-PGG rats 5 weeks after surgery. Nuclei are stained by DAPI. The phenotype was confirmed in *n* = 3 animals per study group. **i** Representative bright-field microscopy images show the PA from a sham rat, an AoB rat, and an AoB-PGG rat 5 weeks post AoB. PGG was detected by FeCl_3_ staining in the PAs of AoB-PGG rats. Results were replicated in 2 animals per group. Box-and-whisker plots overlaid with dot plots (**c-d**, **f**, **g**) show individual data points, mean (rectangle), median (line within the box), lower and upper 25% quartiles (limits of the box), 1.5 IQR (whiskers) and outliers (if applicable). *Statistics*: **c**, **d**, **f**, **g** Unpaired two-tailed Mann–Whitney U test. Source data are provided as a Source Data file.

To test this notion, we investigated the therapeutic potential of elastin stabilization by PGG in an established rat model of PH-LHD subsequent to supracoronary aortic banding (AoB)^66,67^. Compared to sham-operated animals, AoB rats developed increased left ventricular systolic pressure (LVSP) and left ventricular hypertrophy (measured as (LV+septum weight (Sw))/body weight (Bw)) 1-week post AoB and increased right ventricular systolic pressure (RVSP) and right ventricular hypertrophy (quantified as RVw/Bw) 3 weeks post AoB (Fig. 5a-d, black and red graphs). σ_t_/ε_t_ and E/ε_t_ curves revealed PA stiffening with progressively decreasing ε_t 1MPa_ at 1, 3, and 5 weeks post AoB (Fig. 5e, f), and histological analyses at week 5 post AoB identified that the walls of PAs in AoB rats contained broken and straightened elastic fibers (Fig. 5h), thus replicating findings in human PH-LHD patients.

Due to its low oral bioavailability^68^ and strong binding to plasma proteins^69^, PGG delivery to specific target sites in vivo requires specific delivery systems^70^. Here, we used a nanoparticle formulation that shields PGG until it reaches its target tissue. The nanoparticles were conjugated with an elastin antibody (EL-PGG-NPs)^65^ to specifically target PGG to damaged elastin fibers in vivo^65,68,71^. EL-PGG-NPs were intravenously administered to rats at weeks 3 and 4 after AoB, i.e., at a time point when PH-LHD had already been established (Fig. 5a; AoB-PGG group). Effective delivery of EL-PGG-NPs to the PA was confirmed postmortem by polyphenol-specific histological FeCl_3_ staining (Fig. 5i). At week 5 post AoB, AoB-PGG animals had a similar increase in LVSP and LV hypertrophy as vehicle-treated AoB rats (AoB-BLN); however, RVSP and RV hypertrophy were markedly reduced (albeit just missing the level of significance in the case of RV hypertrophy) and did not differ from corresponding values in sham controls (Fig. 5c, d; blue and green graphs). EL-PGG-NP treatment also restored at large PA tensile properties as determined by σ_t_/ε_t_ and E/ε_t_ curves, increased ε_t 1MPa_ (Fig. 5e, f) and normalized – at least in part – pulmonary arterial wall thickness as well as structure and crimping of elastic fibers in the conduit PA wall (Fig. 5g, h, Suppl. Fig. 10). Importantly, these beneficial effects of EL-PGG-NPs were also evident when comparing ε_t 1MPa_ in 5-week EL-PGG-NP-treated AoB rats vs. 3-week untreated AoB rats (Fig. 5f), demonstrating that PGG not only prevented progression but also reversed vascular stiffening in this model of PH-LHD.

**Fig. 6:**
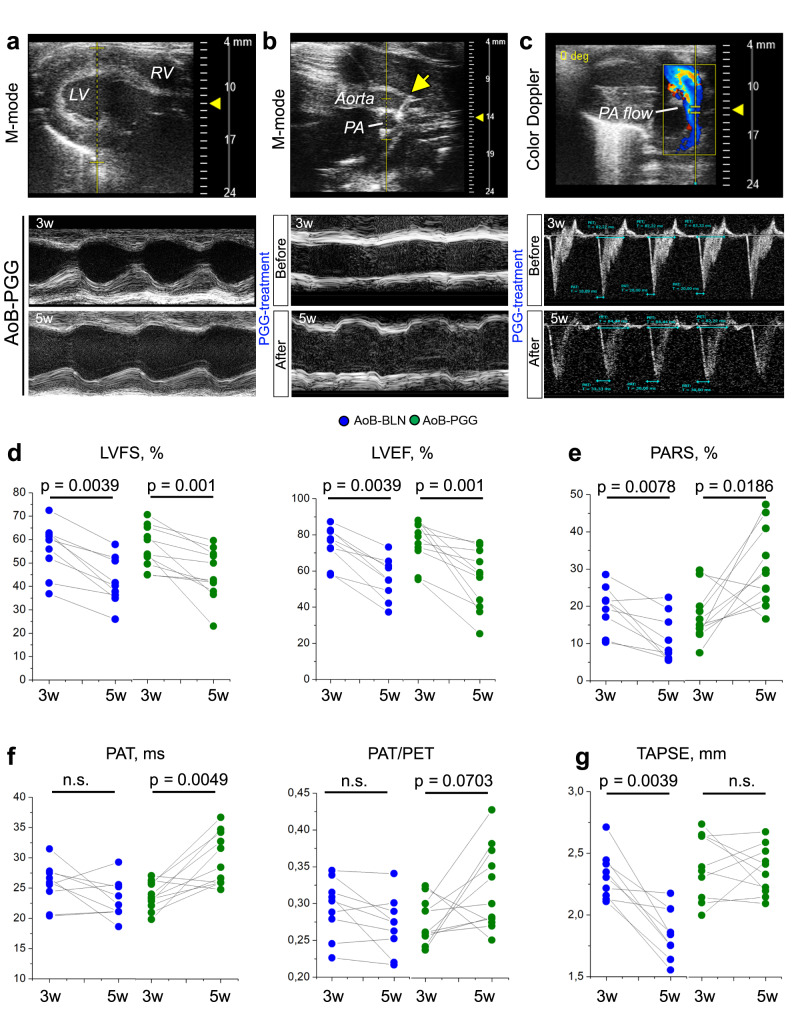
Treatment with PGG improves pulmonary arterial biomechanics and hemodynamics in a rat model of PH-LHD. **a** Representative M-mode images acquired by transthoracic echocardiography display dimensions of the LV walls and LV cavity in an AoB-PGG rat at 3 weeks and 5 weeks after surgery. In comparison to 3 weeks, LV shortening was notably reduced at 5 weeks. **b** M-mode images show PA distensibility in an AoB-PGG rat before (3w) and after (5w) PGG treatment. Yellow arrow points to the clip on the aorta. **c** Representative images show pulmonary blood flow as detected by pulse-wave and color Doppler echocardiography and the analysis of pulmonary acceleration time (PAT) and pulmonary ejection time (PET) parameters in an AoB-PGG rat before (3w) and after (5w) PGG treatment. **d**–**g** Line graphs show longitudinal changes in left ventricular fractional shortening (LVFS), left ventricular ejection fraction (LVEF), pulmonary artery radial strain (PARS), PAT, PAT/PET, and tricuspid annular plane systolic excursion (TAPSE) in AoB-BLN (*n* = 9) and AoB-PGG (*n* = 11) animals before (3w) and after (5w) treatment with either vehicle or PGG. Changes between 3 and 5 weeks were detected as follows: LVFS and LVEF (*p* = 0.0039 for AoB-BLN and *p* = 0.001 for AoB-PGG), PARS (*p* = 0.0078 for AoB-BLN and *p* = 0.0186 for AoB-PGG), PAT (*p* = 0.0049 for AoB-PGG), PAT/PET (*p* = 0.0703 for AoB-PGG), and TAPSE (*p* = 0.0039 for AoB-BLN). *Statistics*: **d**–**g** Two-tailed Wilcoxon matched-pairs signed rank test. Source data are provided as a Source Data file.

This notion was further supported by longitudinal transthoracic echocardiography. In line with progressive LV failure, left ventricular fractional shortening and left ventricular ejection fraction (LVFS and LVEF, respectively, Fig. 6a, d) were decreased in both elastin antibody-conjugated NPs lacking PGG (EL-BLN-NP)- and EL-PGG-NP-treated rats at 5 weeks post AoB compared to pretreatment values at 3 weeks post AoB. In EL-BLN-NP rats, PA distensibility (assessed as PA radial strain; Fig. 6b, e) and tricuspid annular plane systolic excursion (TAPSE, Fig. 6g) decreased in parallel, indicating progressive PH-LHD, while the pulmonary acceleration time (PAT) and PAT to pulmonary ejection time (PET) ratio (PAT/PET, Fig. 6c, f) remained largely unchanged. In contrast, EL-PGG-NP-treated rats showed an increase in PA distensibility and PAT, and stabilization of TAPSE over the same time interval. As such, PGG treatment improved PA biomechanics and pulmonary hemodynamics even in the face of progressively deteriorating LV function.

**Fig. 7:**
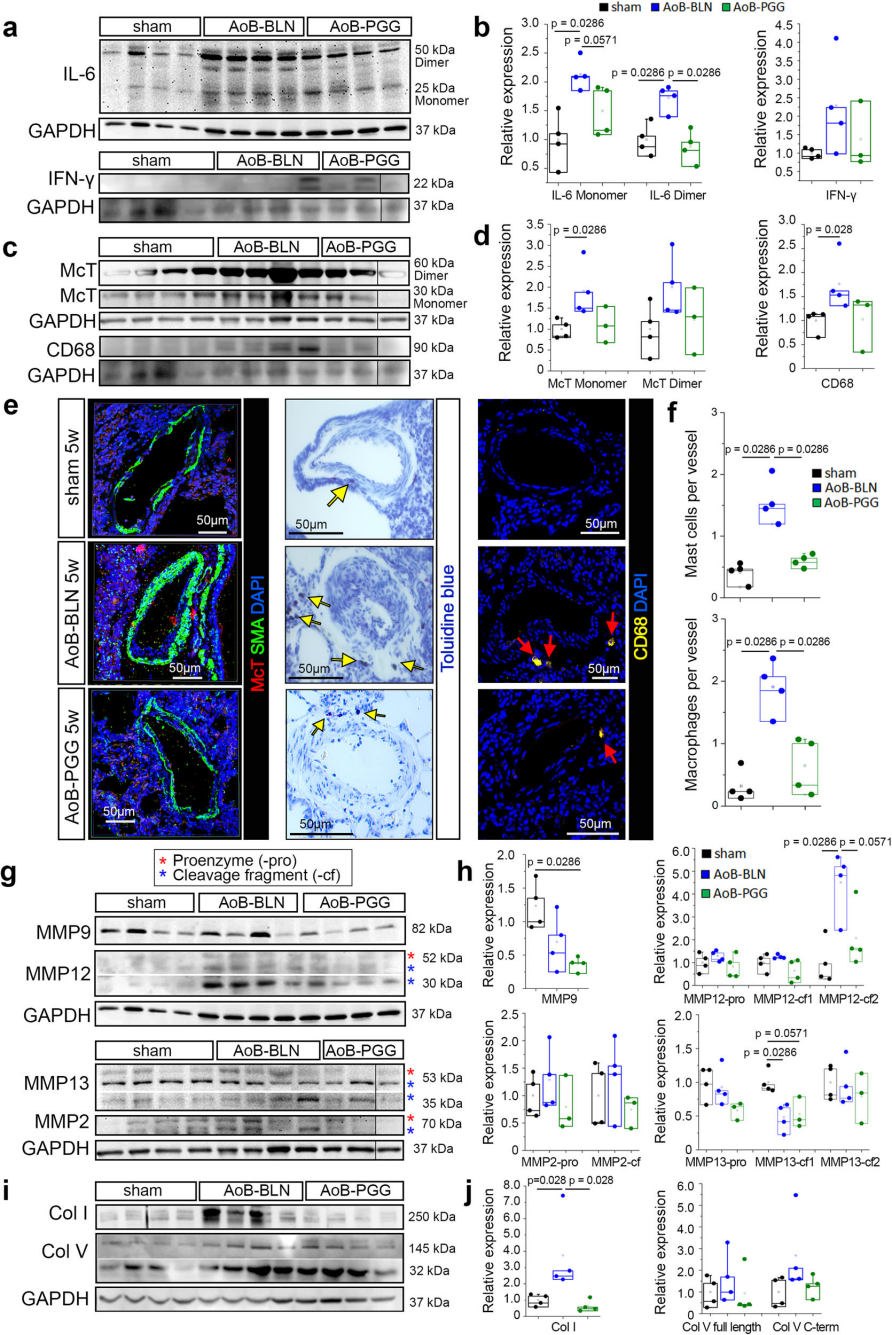
PGG treatment restores homeostasis in lungs of PH-LHD rats. **a** Immunoblots show protein levels of IL-6 (50 kDa dimer and 25 kDa monomer) in lungs of sham (*n* = 4), AoB-BLN (*n* = 4), and AoB-PGG (*n* = 4) animals, IFN-γ (22 kDa) in lungs of sham (*n* = 4), AoB-BLN (*n* = 4), and AoB-PGG (*n* = 3) animals, and corresponding loading control GAPDH (37 kDa). **b** Box-and-whisker plots show quantitative densitometric data for IL-6 (dimer and monomer) and IFN-γ expression normalized to GAPDH and to the mean of the donor controls. IL-6 protein expression differs between groups as follows: monomer (*p* = 0.0286 for AoB-BLN vs. sham, *p* = 0.0571 for AoB-PGG vs. AoB-BLN) and dimer (*p* = 0.0286 for AoB-BLN vs. sham, *p* = 0.0286 for AoB-PGG vs. AoB-BLN). **c** Immunoblots show protein levels of McT (60 kDa dimer and 30 kDa monomer), CD68 (90 kDa), and loading control GAPDH (37 kDa) in lungs of sham (*n* = 4), AoB-BLN (*n* = 4), and AoB-PGG (*n* = 3) animals. **d** Box-and-whisker plots show quantitative densitometric data for McT (dimer and monomer) and CD68 expression (normalized to GAPDH and to the mean of the donor controls). McT monomer and CD68 protein expression differ between groups as follows: *p* = 0.0268 for AoB-BLN vs. sham. **e** Images show mast cells visualized by McT (red) immunofluorescence or Toluidine blue (dark violet) staining, and macrophages detected by CD68 (yellow) immunofluorescent staining in the perivascular space of PAs in lungs of sham, AoB-BLN, and AoB-PGG rats. SMA (green) marks SMCs in the arterial wall, DAPI (blue) marks nuclei. Yellow arrows point to quantified mast cells and red arrows point to quantified macrophages. **f** Box-and-whisker plots show number of mast cells and macrophages surrounding pulmonary vessels of <200 µm diameter in lungs of sham (*n* = 4), AoB-BLN (*n* = 4), and AoB-PGG (*n* = 4) animals (*p* = 0.0268 for AoB-BLN vs. sham and for AoB-PGG vs. AoB-BLN). **g** Immunoblots show protein levels of MMP9 (82 kDa) and MMP12 (54 kDa proenzyme and 45 and 29 kDa cleaved fragments) in lungs of sham (*n* = 4), AoB-BLN (*n* = 4), and AoB-PGG (*n* = 4) animals, and MMP2 (70 kDa proenzyme and 65 kDa cleaved fragment) and MMP13 (53 kDa proenzyme and 48 kDa and 35 kDa cleaved fragments) in lungs of sham (*n* = 4), AoB-BLN (*n* = 4), and AoB-PGG (*n* = 3) animals, and corresponding GAPDH (37 kDa) loading controls. **h** Box-and-whisker plots show quantitative densitometric data for the expression of MMP2, MMP9, MMP12, and MMP13 proenzymes (-pro) and cleaved fragments (-cf) normalized to GAPDH and to the mean of the donor controls. MMP expression differs between groups as follows: MMP2 (*p* = 0.0286 for AoB-PGG vs. sham), MMP12-cf2 (*p* = 0.0286 for AoB-BLN vs. sham and for AoB-PGG vs. AoB-BLN), MMP13-cf1 (*p* = 0.0286 for AoB-BLN vs. sham, *p* = 0.0571 for AoB-PGG vs. sham). **i** Immunoblots show protein levels of Col I (250 kDa), Col V (145 kDa full length and 32 kDa C-terminal cleaved fragment), and loading control GAPDH (37 kDa, corresponding to Col V blot) in lungs of sham (*n* = 4), AoB-BLN (*n* = 4), and AoB-PGG (*n* = 4) animals. **j** Box-and-whisker plots show quantitative densitometric data for the expression of Col I normalized to Ponceau S and Col V (full length and C-terminal fragment) normalized to GAPDH and to the mean of the donor controls (*p* = 0.0286 for AoB-BLN vs. sham and for AoB-PGG vs. AoB-BLN). Box-and-whisker plots overlaid with dot plots (**b**, **d**, **f**, **h**, **j**) show individual data points, mean (rectangle), median (line within the box), lower and upper 25% quartiles (limits of the box), 1.5 IQR (whiskers) and outliers (if applicable). *Statistics*: Unpaired two-tailed Mann-Whitney U test. Source data are provided as a Source Data file. Full scan blots are provided in Suppl. Fig. 12.

In consideration of previous work which demonstrated that PGG-elastin therapy reduces inflammation and restores arterial homeostasis in rodent models of advanced aortic aneurysm^65,72^, we next evaluated the effect of PGG treatment on markers of inflammation in the lungs of PH-LHD rats. Relative to vehicle-treatment, PGG reduced protein levels of interleukin 6 (IL-6) and interferon-gamma (IFN-γ) as well markers of immune cells, namely mast cell tryptase (McT) and the pan-macrophage marker CD68 in AoB rats (Fig. 7a-e). Consistently, quantitative assessment of mast cells and macrophages in lung histological sections by Toluidine blue and CD68 staining, respectively, revealed significant although incomplete reversal of mast cell and macrophage infiltration in PGG-treated AoB rats (Fig. 7e-f). As inflammatory cells may promote elastolysis, ECM remodeling, and ultimately collagen production via increased expression and activation of MMPs^73^, we also probed for the effect of PGG on lung MMPs and collagens. In AoB rats, PGG treatment further downregulated MMP9 and reduced the cleaved (active) form of MMP12 with similar trends in full-length and cleaved MMP2 and MMP13. These findings suggest that PGG not only shields elastic fibers from degradation but also prevents elastolysis by reducing the expression of MMPs which may be linked to decreased markers of lung inflammation. Finally, PGG normalized levels of collagen type I and (albeit not significantly) collagen type V in AoB rats. Taken together, these results demonstrate that PGG treatment largely restored lung vascular homeostasis in a preclinical model of PH-LHD.

Considering that elastic fiber fragmentation may at least in part be mediated by increased expression of MMPs, we finally probed for the effect of the broad spectrum MMP inhibitor Doxycycline (Dox, 30 mg/kg Bw in drinking water during weeks 3–5 post-surgery) in AoB rats. Testing of PA tensile properties revealed σ_t_/ε_t_ and E/ε_t_ curves in Dox-treated AoB rats that were comparable to sham controls (Suppl. Fig. 11a–a’). As such, the effects of Dox treatment on PA biomechanical competences were comparable to those of PGG, in that Dox increased ε_t 1MPa_ (Suppl. Fig. 11b). Analogous to PGG, this effect was associated with reduced thickening of the PA wall (Suppl. Fig. 10), and normalized RV hemodynamics and weight (Suppl. Fig. 11c–d). Notably, however, and in contrast to PGG, Dox also reduced LVSP and showed a trend to further increase LV hypertrophy, findings that are in line with previous reports that Dox aggravates cardiac hypertrophy and dysfunction in mice with aortic constriction^74^.

## Discussion

In this study, we identified impaired PA biomechanical competences as a hallmark of PH-LHD. PA stiffening in PH-LHD is associated with a dysregulation of ECM gene expression, a loss and decrimping of elastin fibers, and increased collagen abundance. Importantly, these changes already emerge in the PAs of LHD patients, suggesting that PA remodeling processes are already initiated prior to PH onset, ultimately causing PA stiffening. The pathophysiological relevance of this process is evident from preclinical experiments in which elastin stabilization restored lung vascular homeostasis and normalized PA biomechanics as well as pulmonary and RV hemodynamics despite persistent LV failure. PA biomechanics thus emerge as an important pathophysiological process that may serve as both a prognostic biomarker and a therapeutic target in PH-LHD.

### ECM remodeling in the PA media is evident in LHD patients prior to the onset of PH and PA stiffening

In healthy arteries, elastic fibers facilitate arterial compliance by straightening in response to load and recoiling in the unloaded state. To this end, elastic fibers are organized into continuous and tortuous lamellae that are circumferentially arranged in the arterial wall. Here, changes in elastic fiber composition could be detected in the PA wall of LHD patients even prior to the onset of PH or altered PA biomechanics. Specifically, the PAs of LHD w/o PH patients showed substantial dysregulation of genes controlling elastic fiber formation (FBN3, MFAP1, MFAP2, MFAP5, and EMILIN2)^47,75,76^ and elastolysis (CTSV and TIMP1)^10,13,77^. Microscopic analyses revealed elastic fiber fragmentation and degradation and increased fiber tortuosity, presumably as progressive fragmentation will result in the retraction of residual fiber pieces.

Although increased expression of collagen type I or accumulation of fibrillary collagen (as detected by SHG imaging) were not yet evident in the PAs of LHD w/o PH patients, existing collagen fibrils were more densely compacted and rearranged in TEM and SHG imaging, respectively, with a higher abundance of AGEs that crosslink collagen fibers^78^. Matrisome analysis showed elevated expression of several collagen types but also collagen-cleaving proteinases (Suppl. Figs. 4 and 6), suggesting an overall increased turnover of fibrillar collagen in LHD w/o PH patients. At this stage of disease, the observed changes in ECM composition did not yet result in altered PA biomechanics, which did not differ between LHD w/o PH patients and healthy-heart controls.

A subgroup analysis of LHD w/o PH patients did not reveal significant differences between patients with borderline PH (20 mmHg < mean PAP < 25 mmHg) and healthy donors or LHD patients with a mean PAP < 20 mm with respect to PA biomechanical competences. Yet, it should be noted that the median values in borderline PH exceeded those in respective controls in all 4 biomechanical parameters. As such, one may expect that much higher n-numbers will reveal differences between these groups, a notion that is supported by our finding that parameters of biomechanical stiffness increase gradually with higher mean PAP values and suggests a continuum rather than a fixed cut-off for the induction of PA remodeling in LHD.

### ECM remodeling progresses and is associated with PA stiffening in PH-LHD patients

In contrast to LHD w/o PH, the PAs of PH-LHD patients displayed altered circumferential tensile properties. Specifically, ex vivo measured stress/strain curves revealed steeper slopes of both the toe region. As the toe region reflects the properties of elastin-enriched material, these findings suggest a loss of load-bearing elastic fibers with an increased stiffness and/or straightening of the residual elastic fibers. Confocal microscopy confirmed extensive rarefaction in large and mid-sized elastic fibers, with a loss of the fiber elastin core at the ultrastructural level. In line with a steeper toe region and an earlier transition of load-bearing elements from elastic to collagen fibers, elastic fibers also appeared straightened, indicating their impaired capacity to recoil, presumably as a result of a constant increase in load and/or an overall stiffening of the ECM due to higher abundance and crosslinking of collagen fibers.

At the transcriptional level, the observed loss of elastic fibers was again (similar to our findings in LHD w/o PH patients) associated with dysregulated expression of elastic-fiber-associated genes and increased expression of elastolytic proteinases, specifically ADAMTS4^44^. Protein expression of elastin digesting MMP9^48^ was already increased in LHD w/o PH PAs, and macrophage elastase MMP12^48^ was additionally elevated in PH-LHD. As we will discuss later, these findings indicate that the homeostasis of elastic fibers becomes impaired early in LHD and may promote the development of PH once elastolysis reaches a critical level at which pulmonary arterial biomechanical competences deteriorate.

Progressive elastolysis was associated with a greater abundance of fibrillar collagen and, accordingly, an increase in the collagen-to-elastin ratio in the PA wall. These histological findings are consistent with our biomechanical analyses demonstrating an increased slope of the linear region of the stress/strain curves reflecting increased PA stiffness due to more and/or stiffer collagen fibers. Indeed, matrisome analysis identified upregulated genes coding for the subunits of 13 different collagen types. Of those, genes coding for fibrillar collagen types I and V, which did not differ between LHD w/o PH samples and healthy-heart controls, were specifically upregulated in PH-LHD patients. The upregulation of type I collagen, the predominant collagen subtype in healthy arterial walls, is typically associated with arterial stiffening in systemic arterial hypertension and PAH^57,58,79,80^, while collagen V controls the fibrillogenesis of type I collagen^57^. Over and above, increased expression of type II and XI fibrillar collagens in PAs of LHD w/o PH and PH-LHD patients may indicate cell de-differentiation and/or chondrogenic trans-differentiation processes in PAs^81^, as these collagens are characteristically expressed in cartilage tissue rather than SMCs^82–84^. Increased formation of fibrillar collagen and aggregation into rods was confirmed with SHG and validated by TEM imaging, and fibrillar collagen colocalized with increased levels of AGE, suggesting crosslinking^59–61^. This result is in line with previous works demonstrating increased serum levels of AGEs in heart failure patients that correlated with disease severity and poor prognosis^85,86^. Overall, these findings identify a loss of elastic fibers and concomitant abundance in fibrillar collagen as characteristics and likely causes of increased PA stiffness in PH-LHD.

### Elastic fiber fragmentation – an early marker and potential mechanism of PA remodeling

In the present study, we identified the fragmentation and degradation of elastic fibers as an early event in PA remodeling that precedes and likely contributes to PA stiffening and impaired arterial biomechanics in PH-LHD. The functional consequences of elastic fiber degradation may, however, extend beyond pure mechanobiology, as fiber degradation is known to mediate arterial remodeling processes consistent with those detected in PH-LHD PAs. Specifically, attachment to elastic fibers is important to maintain SMCs in their contractile phenotype^1–3^; in the PAs of LHD w/o PH and PH-LHD patients, however, SMC-to-fiber attachment was markedly reduced, potentially promoting SMC migration. Elastic fiber fragmentation also releases bioactive elastin-derived peptides (EDPs) that act through the membrane-bound elastin receptor complex and regulate a series of biological processes. Although the role of EDPs was not a focus of our current study, it is tempting to speculate that the observed PA remodeling processes in PH-LHD may be induced or enhanced by EDP-mediated signaling. For example, EDPs can regulate elastic fiber degradation through a positive feedback mechanism by stimulating cytokine signaling and activating inflammatory cells, which in turn secrete proteases to digest elastic fibers^7,87,88^. EDPs have also been implicated in the induction of hyperglycemia^89^, and the resulting high glucose levels may fuel the formation of AGEs, which were found in abundance in the PAs of both LHD w/o PH and PH-LHD patients.

As such, elastic fiber fragmentation may constitute an early event as well as a relevant pathophysiological mechanism of PA remodeling. Specifically, elastolysis appears to occur early in left heart failure but may initiate a cascade of remodeling processes, including progressive loss of elastin and increased production of fibrillar collagen, that ultimately cause PA stiffening and PH secondary to LHD. If this is indeed the case, the prevention of elastin degradation may present a promising strategy to attenuate ECM remodeling, improve arterial biomechanics, and potentially reduce PH and RV load.

### Elastic fiber stabilization rescues PA biomechanics, restores lung vascular homeostasis, and attenuates PH

To test the effect of elastic fiber stabilization on PA biomechanics and PH, we aimed to counteract elastic fiber degradation in PAs. To this end, polyphenols such as PGG and epigallocatechin gallate have shown considerable promise due to their ability to induce elastin synthesis, organization, and crosslinking while concomitantly blocking the activity of elastin-degrading enzymes^90^. Accordingly, local application of PGG could effectively decrease elastic fiber degeneration and reduce aneurysmal expansion in a rat AAA model, providing proof-of-principle for the ability of PGG to stabilize elastic fibers and improve arterial biomechanics in vivo^63^. This approach was recently further refined by PGG loading into EL-PGG-NPs. As these antibodies will preferentially bind to degrading elastin fibers, this system allows specific targeting of PGG to the sites of vascular damage^65^. In the rat AAA model, systemic delivery resulted in specific accumulation of EL-PGG-NPs at the AAA site and proved efficacious to warrant long-term aortic biomechanical stability in vivo, with the inhibition of macrophage infiltration and MMP activity and the restoration of an intact elastic lamina^65^.

As PGG has thus far not been tested on PAs, we assessed its effects on elastic fibers and arterial biomechanics in human PAs ex vivo. In both naïve PAs and in PAs treated with elastase, PGG increased elastin content and improved arterial biomechanics, as demonstrated by a shift in load-bearing elements from collagen to elastic fibers, further substantiating the therapeutic promise of PGG. Next, we probed for the effects of EL-PGG-NPs in vivo in a rat AoB model that replicates the cardinal features of human PH-LHD, namely, PA stiffening with a shift of load-bearing elements from elastin to collagen, increased RVSP, and RV hypertrophy. Systemic administration of EL-PGG-NPs yielded effective delivery of PGG to the PAs of AoB rats and improved PA biomechanics and RV hemodynamics in vivo. Importantly, and in line with the proposed targeting of EL-PGG-NPs to degrading fibers, this approach proved beneficial in a therapeutic setting, i.e., when EL-PGG-NPs were delivered at a time point when not only LHD but also PA stiffening, PH, and RV hypertrophy were evident. Moreover, EL-PGG-NPs not only prevented disease progression but also partially reversed impaired PA biomechanics and, as such, PH and RV hypertrophy. Specifically, EL-PGG-NP treatment right-shifted PA stress/strain curves and increased ε_t 1MPa_, indicating a reverse shift in load-bearing elements from collagen back to elastin. In line with this interpretation, EL-PGG-NP treatment also increased PA radial strain in vivo. The rescue of PA biomechanics was associated with improved RV hemodynamics and hypertrophy, highlighting the relevance of PA stiffening in the progression of PH and RV hypertrophy in PH-LHD. Dox replicated the effects of PGG on PA biomechanical competences, RV hemodynamics, and PA/RV remodeling, indicating an important role for MMP-mediated elastolysis in elastin degradation in PH-LHD. Yet, albeit Dox is already clinically approved, PGG may hold greater long-term therapeutic promise because a) of its dual mode of action, in that it both shields elastic fibers and reduces the expression of MMPs, and b) as – in contrast to Dox - it has no documented adverse effects on the left ventricle.

In the present study, we demonstrate considerable PA stiffening in patients with PH-LHD that is associated with a switch in load-bearing elements from an elastin to a collagen fiber-dominated tissue and altered ECM composition. Corresponding changes at the transcriptomic and microscopic levels were already evident in LHD patients prior to the onset of PA stiffening and PH, indicating that changes in PA ECM composition constitute an early event and potential pathomechanism in disease progression. In line with this notion, therapeutic stabilization of elastic fibers rescued PA biomechanics and attenuated PH, thus consolidating the pathogenic relevance of PA stiffening for RV hemodynamic alterations and highlighting the therapeutic potential of ECM-targeted interventions for the treatment of pulmonary vascular diseases associated with ECM remodeling and PA stiffening.

### ECM remodeling and PA stiffening in the pathophysiology of PH

While different underlying diseases cause PH via different pathomechanisms, they commonly share structural and mechanical changes in the pulmonary vasculature. Among the common characteristics of PA wall remodeling are increased proliferation and migration of vascular cells as well as inflammation and infiltration of immune cells. As discussed below, both processes are tightly interconnected with ECM remodeling and matrix stiffness.

In PH, PA stiffness is primarily increased by overproduction, imbalanced turnover, and cross-linking of collagens and a parallel increase in elastolysis due to increased enzymatic activity of proteolytic enzymes such as MMPs and elastases (^58,91^, and current study). Inflammation and biomechanical microinjuries seem to play a key role in initiating this imbalance in proteolytic enzymes^92^. Specifically, it has been proposed that initial injury to PA endothelial cells by shear stress or inflammation may disrupt the endothelial barrier and thus, allow for entry of growth factors from the circulation into the vascular wall where they will induce cellular hypertrophy and hyperproliferation and stimulate the release of serine elastases and MMPs as well as the production of collagens by PA SMCs^52,93,94^ and fibroblasts^95^. In addition to their direct role in ECM remodeling, MMPs also regulate the processing and release of inflammatory cytokines^52^, growth factors, and matrikines^96^ from the ECM, thus promoting proliferation of vascular cells as well as infiltration of macrophages and neutrophils^97,98^, which, in turn, release MMPs and serine elastases. Furthermore, ECM remodeling promotes the proliferation and migration of vascular cells directly, first by impaired cell-matrix contacts (as demonstrated in the present study) and second via mechanosensitive signaling pathways such as HIPPO^23^. Finally, proximal PA stiffening increases vascular shear stress and inflammation in distal PA regions by amplifying pulse wave transmission^99,100^, thus promoting distal (and in turn again proximal) vascular remodeling through the mechanical interplay between proximal and distal PA regions^23^. As such, ECM remodeling and the resulting PA stiffening are at once critically involved in several detrimental feed-forward loops that drive and maintain both proximal and distal lung vascular remodeling in PH.

In line with this notion, therapeutic administration of PGG in PH-LHD rats not only stabilized elastic fibers and restored biomechanical competences in conduit PAs, but also normalized distal vessel morphology, perivascular infiltration of immune cells, and the homeostasis of inflammatory cytokines, proteolytic enzymes, and collagens in the lung.

### Study limitations

Due to the limited size of PA material obtained from human patients, PA biomechanical competences had to be assessed by uniaxial, rather than state-of-the-art biaxial tensile testing in the present study. For comparability, the same method was used for rat PAs. It should be considered that the uniaxial assessment of PA biomechanics does not capture multi-axial ECM dynamics, thereby limiting the accuracy, sensitivity, and interpretation of the biomechanical measurements. While the study can thus not yield information on the anisotropic behavior of arterial tissue in the setting of PH-LHD, the fact that uniaxial stress testing was able to reliably detect significant differences between study groups in both humans and rats attests to the qualitative validity of the reported findings. It should be emphasized that the interpretation of the uniaxial stress/strain relationships in terms of predominantly elastin- and collagen-dominated material competences is an oversimplification of the considerable complexity of ECM regulation by elastin-associated proteins and matrix molecules involved in collagen fibrillogenesis that is also hinted at by our transcriptomic analyses. Further, potential intrinsic differences in biaxial connectivity between controls and diseased groups (e.g., increased collagen cross-linking due to AGEs etc.) may not be captured by uniaxial tensile tests, posing an additional limitation to the interpretation of biomechanical results even when calibrated against corresponding uniaxial controls. While our data are to our knowledge the first to demonstrate increased PA stiffness in PH-LHD, further in-depth analysis of the molecular changes and associated biomechanical effects in the PA wall is warranted to provide for a more comprehensive picture of PA stiffening.

In the present study, we used doxycycline as a broad-spectrum MMP inhibitor. While FDA approval of Dox as an antibiotic attests to the translational relevance of our findings, it should be recognized that Dox is not MMP specific, and that off-target effects may have contributed to its effects on the pulmonary circulation and the left and right ventricle, respectively.

PGG-delivering nanoparticles have been previously shown to target the arterial wall via vasa vasorum from the adventitial side^65,71^. As such, excessive deposition of collagen in the adventitia may be expected to limit the targeting of NPs to the arterial media, and early treatment with PGG may hence be more efficient than late intervention in PH-LHD. Alternatively, elastin stabilization may be combined with parallel strategies to reduce collagen deposition as a synergistic approach.

## Methods

### Collection of human PA samples and clinical data analyses

Human tissue samples and blood were collected following approval by the Ethics Committee of the Charité-University Medicine Berlin (EA4/035/18 and EA2/043/19) and with the written informed consent of the patients. The study was conducted in accordance with the principles of the Declaration of Helsinki. A DSMB was not involved; participants did not receive compensation. Specimens from the pulmonary trunk (hereafter referred to as PA samples) were collected during orthotopic heart, lung, or combined heart-lung transplantations from donors (healthy-heart control group, *n* = 33), patients with LHD without PH (LHD w/o PH group, *n* = 41), patients with PH due to LHD (PH-LHD group, *n* = 49), and patients with PAH (PAH group, *n* = 4) when the length of the PA was adjusted before anastomosis. The diagnosis of PH or PAH was verified by data from right heart catheterization (RHC) obtained within 6 months prior to transplantation using a cutoff value of a mean PAP of 25 mm Hg^101^.

Blood samples were collected from LHD patients (*n* = 18) and healthy donors (*n* = 4) in BD Vacutainer Plastic K2EDTA tubes (Fisher Scientific). Cells were removed from the plasma by centrifugation for 10 min at 4,000 rpm using a refrigerated (4 °C) centrifuge. The supernatant (plasma) was collected and stored at −80 °C.

Demographic data for age and sex did not differ significantly between healthy-heart donors and LHD w/o PH and PH-LHD patients and are reported in Suppl. Table 2 and Suppl. Fig. 3. Underlying diseases are listed in Suppl. Table 2 and comprised ischemic cardiomyopathy and nonischemic cardiomyopathy for patients with LHD w/o PH and PH-LHD^102^ and idiopathic PAH and associated PAH for the PAH group. Pulmonary hemodynamics for LHD w/o PH, PH-LHD, and PAH patients are summarized in Suppl. Table 3.

PA dimensions were quantified based on 3D reconstructions of CT images using JiveX Demonstration Client software v. 4.7.1 (VISUS). The control cohort for these measurements comprised patients with no structural or functional cardiac abnormalities at rest undergoing coronary bypass surgery. Pulmonary trunk dimensions were assessed just proximal to the PA bifurcation at the level of the aorta. PA diameter was determined in three different orientations on cross-sectional images, and PA dimension was calculated as the arithmetic average.

### Biomechanical testing of human PA samples

#### Uniaxial tensile test

Following harvesting, human PA samples were kept in normal saline on ice and assessed for biomechanics within 2-4 h. Samples were checked for holes or tears, the presence of which resulted in exclusion from biomechanical testing. Loose connective and adipose tissue were carefully removed, and sample dimensions (length and width) were measured using a digital caliper. The circumferential tensile properties of PAs were assessed using a MyoDynamics Muscle Strip Myograph System (840DM, Danish Myo Technology) with controlled temperature (37 °C) and aeration. To this end, circumferential rectangular sections (2×8 mm) were excised from the pulmonary trunk and mounted at 5 mm length (Suppl. Fig. 1a–b). Arterial tissue was preconditioned by 5 extension-relaxation cycles at a low force of 5-10 mN to avoid damaging the elastin material. After sample equilibration at a baseline force of 1 mN, an automated displacement (ΔL) of 4 mm was applied at a rate of 0.5 mm/s. In each tested sample, a full strain (ε) of 80% was achieved. The generated force (F) was recorded in real-time by a data acquisition system (PowerLab 4/35-1605 and LabChart Pro v. 8, ADInstruments) and displayed as a force/displacement curve. Between 2 and 4 specimens of each PA were tested, and the results for these technical replicates were averaged. Recorded force/displacement curves were converted to engineering stress/strain curves, where stress (σ) was calculated as F divided by the initial cross-sectional area of the tested sample, and strain (ε) was calculated as ΔL divided by the initial length of the tested sample. The cross-sectional area was determined as the product of sample width in the short axis (2 mm) and PA wall thickness as assessed in histological sections and adjusted for tissue shrinkage resulting from histological processing. Next, engineering σ/ε curves were converted into true stress σ_t_ versus true strain ε_t_ curves. σ_t_ was calculated based on the instantaneous cross-sectional area as σ_t_ = σ(1+ ε) under the assumption that the sample volume is conserved and deformation happens uniformly. ε_t_ was calculated as ε_t_ = ln(1+ε). σ_t_ versus ε_t_ curves were then fitted with a 6-degree polynomial regression. Instantaneous Young’s modulus E as a measure of stiffness was calculated from the 1^st^ derivative of the polynomial equation and plotted against true strain ε_t_. Group averages of σ_t_ versus ε_t_ curves and E versus ε_t_ curves are provided in the figures. For statistical comparisons and correlations, we analyzed stiffness E versus true strain ε_t_ curves in two ways: (a) as stiffness E at a true strain of 0.5 (E_0.5_), and (b) as true strain ε_t_ at 1 MPa E (ε_t 1MPa_).

#### Effects of PGG on PA biomechanics

The effect of PGG on PA biomechanical properties was assessed by ex vivo culture of PA specimens followed by uniaxial tensile testing. Freshly isolated PAs were handled under sterile conditions. One piece of the artery was excised and directly underwent uniaxial tensile testing to record PA biomechanics at baseline. The rest of the PA was prepared as 2×8-mm strips and placed in a 12-well plate for ex vivo culture in serum-free Dulbecco’s modified Eagle’s medium (DMEM, Gibco) supplied with penicillin-streptomycin (Thermo Fisher) at 37 °C with controlled humidity at 21% O_2_ and 5% CO_2_. PGG (penta-O-galloyl-β-D-glucose hydrate, Sigma-Aldrich) was dissolved in dimethyl sulfoxide (DMSO, Sigma-Aldrich) as a stock solution and added to the medium at a final concentration of 0.1%. Similar concentrations of PGG have previously been shown to have minimal cytotoxicity^63^. Type IV elastase from porcine pancreas (E0258, Sigma-Aldrich) was prepared in Dulbecco’s phosphate-buffered saline (DPBS, Gibco) as a stock solution, and 1 U was added to the medium based on the results from previous studies in porcine aortas demonstrating effective digestion of the majority of elastic fibers over 24 h^103,104^. The following four treatment conditions were tested: control, elastase only, PGG only, and PGG prior to elastase. PGG treatment was performed for 1 h at 37 °C prior to culturing PAs for 24 h in the presence or absence of elastase. Control samples were incubated with the corresponding concentrations of DMSO.

### Rat model of PH secondary to LHD

All animal procedures were approved by the local governmental animal care and use committee (Landesamt für Gesundheit und Soziales (LaGeSO), Berlin) under protocol number G0030/18. All experiments were performed in accordance with the ARRIVE guidelines and the “Guide for the Care and Use of Laboratory Animals” (Institute of Laboratory Animal Resources, 8th edition 2011). The animals were kept in groups at controlled temperature, humidity, and 12 h light/dark cycle conditions in cages equipped with wooden bedding, shelters, and nest-building material, and had ad libitum access to drinking water and food (V1534-300, Ssniff Spezialdiaeten).

#### Surgical procedure

Congestive heart failure was surgically induced in juvenile Sprague-Dawley rats (5-week-old animals of approx. 100 g Bw) from Janvier Labs by supracoronary AoB as previously described^105^. In brief, rats were anesthetized by intraperitoneal injection of ketamine (87 mg/kg Bw) and xylazine (13 mg/kg Bw), and adequate depth of anesthesia was checked regularly by the toe pinch test. In rats in the AoB group, a titanium clip of 0.8-mm inner diameter was placed on the ascending aorta. Perioperatively, rats were mechanically ventilated with room air at tidal volumes of 6 mL/kg Bw via tracheotomy as previously described^106^. Sham animals underwent all anesthetic and surgical procedures except for clip implantation and served as controls. Animals received moisturizing ointment for their eyes during anesthesia, as well as pre- and postoperative analgesia (carprofen, 5 mg/kg Bw intraperitoneally daily for 1 week) and postoperative antibiotics (amoxicillin, 500 mg/L in drinking water). After surgery, animal health was monitored daily in accordance with an approved score sheet, and animal weight was assessed twice a week. At 1, 3, or 5 weeks after surgery, endpoint measurements were performed, including invasive hemodynamic monitoring. After animals were euthanized under deep anesthesia by exsanguination, measurement of heart weight, postmortem control of clip placement, testing of PA biomechanical properties and PA histological analyses were performed as specified below.

#### Preparation of PGG loaded bovine serum albumin (PGG-NPs) and blank nanoparticles (BLN-NPs), and antibody conjugation

PGG-loaded bovine serum albumin (BSA) NPs (PGG-NPs) and blank BSA NPs (BLN-NPs) were prepared as previously described^65^. In brief, 250 mg of BSA (Seracare, Milford, MA) was dissolved in 4 mL of DI water, and PGG (125 mg PGG (Ajinomoto Omnichem) dissolved in 400 µL dimethyl sulfoxide) was added while stirring, followed by 37 µL of 8% glutaraldehyde for crosslinking at room temperature. The resultant nanoparticle size was previously shown to range between 220–240 nm^71^ with a minimal increase in size from 236 ± 9.5 nm to 240.6 ± 18.2 nm due to conjugation with anti-elastin antibody (see below). After 1 h of stirring, the NP mixture was added slowly to 24 mL of ethanol (Sigma, St. Louis, MO) under continuous sonication (Omni Ruptor 400 Ultrasonic Homogenizer, Omni International Inc, Kennesaw, GA) for 30 min. The pellet of PGG-NPs was obtained after centrifugation at 6000 rpm for 10 min. Blank (BLN) nanoparticles were prepared analogously without PGG addition.

PGG-NPs and BLN-NPs thus prepared were next conjugated with degraded elastin targeting rabbit anti-rat elastin polyclonal antibody (in-house developed at Clemson University) overnight as described previously^65^. Briefly, 10 mg of PGG-loaded or blank NPs were PEGylated with 2.5 mg α-maleimide-ω-N-hydroxysuccinimide ester poly (ethylene glycol) (mPEG-NHS, M.W. 2000, Nanocs, NY, U.S.A.) at room temperature for 1 h under gentle vortexing. Twenty μg of an in house-made anti-elastin antibody (EL) was thiolated with 68 μg Traut’s reagent (G-Biosciences, Saint Louis, MO) dissolved in (4-(2-hydroxyethyl)−1-piperazineethanesulfonic acid (20 mM HEPES) buffer (pH=9.0). The mixture was incubated for 1 h at room temperature. Next, the thiolated antibody was added to the PEGylated NPs and conjugated overnight at 4 °C under slow rocking.

#### EL-PGG-NP injection

PGG-loaded nanoparticles conjugated with elastin antibodies (EL-PGG-NPs) prepared in PBS were injected via the tail vein into AoB rats (10 mg/kg Bw) 3 and 4 weeks after AoB surgery. Control rats received elastin antibody-conjugated blank nanoparticles (EL-BLN-NPs).

#### Doxycycline (Dox) treatment

Rats were treated daily with Dox (30 mg/kg Bw in drinking water)^107^ during weeks 3-5 post-surgery. Control rats received water without Dox.

#### Transthoracic echocardiography

Echocardiography was performed in AoB-PGG or corresponding control rats prior to and after EL-PGG-NP and EL-BLN-NP treatment, respectively, at 3 and 5 weeks after AoB. In brief, rats were anesthetized with isoflurane (1.5% supplemented with O_2_ at 2 L/min), eyes were protected with a moisturizing ointment, and temperature and electrocardiograms were continuously monitored. Image acquisition was performed using a MX250 transducer with a Vevo 3100 preclinical imaging system (FUJIFILM VisualSonics). LVFS was evaluated from the time motion display (M-mode) and LVEF was evaluated from the 2D ultrasound image display (B-mode) acquired in the parasternal long-axis (PLAX) view (Fig. 6a). PA imaging was performed in a modified PLAX view obtained by B-mode shifting of the transducer probe toward the RV outflow tract (Fig. 6c). PA flow was assessed by pulsed-wave Doppler imaging. TAPSE was assessed in the M-mode by measuring the distance of tricuspid annular movement between end-diastole and end-systole in the mid-esophageal four-chamber view. The aortic arch view was used to control correct clip placement on the aorta and to record flow profiles in the ascending and descending aorta by pulsed-wave Doppler imaging and to acquire M-mode images from the transverse section of the PA (Fig. 6b). PA dimensions (maximum and minimum diameter of the pulmonary trunk) and pulmonary arterial blood flow velocity characteristics (PAT and PET) were analyzed using Vevo LAB v. 3.1.1 (FUJIFILM VisualSonics) analysis software. The pulmonary arterial radial strain was calculated as PARS = (D_Max_-D_Min_)/D_Min_, where D_Max_ and D_Min_ are the maximum and minimum PA diameters measured in the modified PLAX view, respectively.

#### Cardiac catheterization and hemodynamics

Animals were anesthetized with ketamine/xylazine, tracheotomized, and ventilated with room air as described above. Following a median thoracotomy, the pericardium was opened, and LVSP followed by RVSP were measured via the heart apex using a microtip Millar catheter, PowerLab 4/35-1605, and LabChart Pro v. 8 software (all ADInstruments).

#### Heart weight

Ventricular hypertrophy was assessed as the weight of the left ventricle (including the septum) and right ventricle normalized to Bw.

#### Ex vivo uniaxial tensile test

Similar to human PA samples, the circumferential tensile properties of rat PAs were assessed on a MyoDynamics Muscle Strip Myograph System (840DM, Danish Myo Technology). Freshly isolated pulmonary trunks were prepared in PBS and dissected into 2-mm-wide rings. Rings were mounted onto the material testing system with the help of hooks. Samples were preconditioned by 5 extension-relaxation cycles (5-10 mN each) and then equilibrated at the baseline pretension of 5 mN. An automated displacement of 5 mm maximal length at a rate of 0.5 mm/s was applied. Force/displacement curves recorded for rat samples were analyzed analogously to curves for human samples, and group averages of σ_t_ versus ε_t_ curves and E versus ε_t_ curves are provided in figures. Due to considerable noise in the long toe region of the σ_t_ versus ε_t_ curves and accordingly, also the E versus ε_t_ curves, the presentation of these curves is restricted to the ascending region, and quantification is solely based on the calculation of ε_t 1MPa_.

### Histology and microscopy

#### Histology

Human or rat PA samples were cryo-embedded in OCT Compound (Tissue-Tek) and sliced into 10-, 20-, or 50-µm transverse sections on a Microm HM560 cryostat. Slides were stored at −20 °C. Before staining, slides were thawed in PBS and fixed with 4% paraformaldehyde (Alpha Aesar, Thermo Fisher Scientific). Elastic and collagen fibers were visualized in 10-µm cryosections with EVG staining (Elastic Stain Kit, ab 1506667, Abcam) according to the manufacturer’s protocol. For PGG detection, 10-µm frozen OCT sections were mounted on positively charged glass slides and rinsed with tap water for 5 min to remove OCT. Sections were stained with 15% FeCl_3_ (Sigma-Aldrich) solution in deionized water for 7 min, washed, and observed directly by light microscopy. Mast cells in rat lungs were visualized by toluidine blue staining on 10-µm sections from paraffin-embedded lungs as follows: deparaffinized and rehydrated lung sections were incubated for 2 min with toluidine blue working solution containing 5 mL of 1% toluidine Blue O (Sigma-Aldrich) in 70% ethanol and 45 mL of 1% NaCl (pH 2–2.5), followed by dehydration in 95% ethanol, clearing in xylene and mounting in DPX Mount (Sigma-Aldrich) for microscopy.

#### Immunohistochemistry

Immunostaining was performed on 10-µm sections as previously described^108^. Nuclei were visualized by DRAQ5 (Abcam ab108410) according to the manufacturer’s instructions or DAPI (1 mg/mL in PBS, Sigma-Aldrich D9542) for 10 min. The following antibodies were used: primary rabbit polyclonal anti-LOX (Thermo Fisher, PA1-46020; validated by manufacturer to react with human and rat protein for use in WB, IHC, IF) and rabbit polyclonal anti-AGE (Abcam, ab23722, species independent reactivity; validated by manufacturer for use in IF) at a 1:100 dilution and secondary goat anti-rabbit Alexa Fluor 568 (Thermo Fisher, A-11036) at a 1:500 dilution. Samples were mounted with Mowiol medium prepared according to Cold Spring Harbor Protocols. Briefly, 2.4 g of Mowiol 4-88 (Sigma-Aldrich) was mixed with 6 g of glycerol (Roth) and 6 mL of dH_2_O and left for several hours at room temperature (RT). After adding 12 mL of 0.2 M Tris-Cl (pH 8.5), heating to 50 °C and mixing were applied to dissolve the Mowiol. The solution was clarified by centrifugation at 5000 g for 15 min and stored at −20 °C. Macrophages and mast cells in rat lungs were visualized on 10-µm paraffin-embedded sections by anti-CD68 or anti-mast cell tryptase (McT) immunostaining. In brief, lung sections were deparaffinized by incubation in Neo-Clear (Sigma-Aldrich) and rehydrated in ethanol at decreasing concentrations. Samples were boiled in Tris-EDTA buffer for 10 min for antigen retrieval. After 1 h blocking in 2.5% goat serum, samples were incubated with either rabbit polyclonal anti-CD68^109^ (AbBiotec, 250594; validated by manufacturer to react with rat protein for use in WB, IHC) or rabbit polyclonal anti-McT (Santa Cruz Biotechnology, sc-32889; validated by manufacturer to react with human and rat protein for use in WB, IHC) primary antibodies diluted 1:300 in PBS containing 10% BSA overnight at 4 °C. Following PBS wash, samples were stained with secondary fluorescently labelled antibodies for 2 h at room temperature. Finally, samples were washed with PBS, stained with DAPI, and mounted with Mowiol medium.

#### Bright-field microscopy

Bright-field imaging was performed on an Axioscope 40 microscope (Zeiss) with an Axiocam 506 color camera (Zeiss) and recorded using ZEN 2 blue edition v. 1.0 (Zeiss) software.

#### Scanning confocal microscopy

An A1Rsi+ confocal microscope and NIS-Elements AR v. 4.40.00 software (Nikon) were used to detect elastic lamellae by autofluorescence (488 nm/0.66 mW laser), DAPI staining (405 nm/0.37 mW laser), DRAQ5 staining (647 nm/1.40 mW laser), and antibody staining (561 nm/1.37 mW laser).

Fibrillary collagen was imaged by SHG imaging on a Leica SP5 II microscope using a × 25 water immersion objective and Leica Application Suite X (LAS X) v. 4.13.0 (Leica Microsystems) software. The SHG signal was generated by a Spectra Physics Ti:Sapphire laser (Mai Tai HP) with a 100-fs pulse width at 80 MHz and wavelength of 910 nm and detected at 450–460 nm as described previously^110^.

#### Transmission electron microscopy

Samples were fixed immediately with 2.5% glutaraldehyde in 0.1 M sodium cacodylate buffer (both Serva) for 30 min at RT and stored at 4 °C. Postfixation was performed with 1% osmium tetroxide (Electron Microscopy Sciences) and 0.8% potassium ferrocyanide II (Roth) in 0.1 Mol/L cacodylate buffer for 1.5 h followed by the dehydration of the samples in a graded ethanol series and the embedding of the samples in Epon resin (Roth). Finally, ultrathin sections with a thickness of 70 nm were stained with uranyl acetate and lead citrate. Samples were examined using a Zeiss EM 906 electron microscope at 80-kV acceleration voltage (Carl Zeiss).

#### Image analyses

EVG staining and anti-LOX or anti-AGE immunofluorescence were quantified based on intensities, areas, and patterns on single-plane images using Fiji-ImageJ-win64 v.2.11.0. Elastic fiber length was measured based on automatic fiber detection with Skeletonizing ImageJ. Elastic fiber tortuosity was calculated as the arc-to-chord ratio in a semiautomated manner using a self-developed ImageJ plugin. For elastic fibers, images were acquired from 20-µm sections as 2D z-stacks at 2 µm steps and reconstructed into 3D images using Arivis 4D v. 2.12.5 software. Elastic particles were first filtered by >1 µm 2D long sides and sorted by volume into groups, and their number and cumulative surface area were quantified and normalized to image volume. The number of DRAQ5-stained nuclei was calculated from the same 3D reconstructions. The elastic fiber networks in Fig. 3a and the Suppl. Video were visualized in 50-µm-thick sections imaged first as a 2D z-stack in 2-µm intervals and reconstructed with Arivis 4D v. 2.12.5 software. The fibrillar collagen particle number, surface area, and volume were detected by SHG from 20 µm sections as 2D z-stacks at 2-µm steps and quantified from 3D-reconstructed images using Imaris 9.2 software.

Toluidine blue labeled mast cells and CD68 positive macrophages in the perivascular space were quantified as previously described^66,111^. Arterial wall thickness was determined on 10-µm sections from paraffin-embedded rat lungs or OCT-embedded human arteries stained with hematoxylin and eosin (Sigma-Aldrich).

### RNA sequencing and matrisome analysis

A global analysis of pulmonary arterial ECM gene expression was performed by total RNA sequencing and subsequent analysis of matrisome genes. Snap-frozen PA specimens were subjected to TRIzol-RNA extraction. RNA quality was assessed by measuring the RNA integrity number (RIN) using a Fragment Analyzer HS Total RNA Kit (Advanced Analytical Technologies, Inc.). Library preparation for total RNA-Seq was performed using TruSeq™ Stranded Total RNA with Ribo-Zero Gold (Illumina, Cat. Nr. RS-122-2301) starting from 1000 ng of total RNA. The size range of the final cDNA libraries was determined by applying the SS NGS Fragment 1- to 6000-bp Kit on the Fragment Analyzer (average 340 bp). Accurate quantification of cDNA libraries was performed by using the QuantiFluor™ dsDNA System (Promega). cDNA libraries were amplified and sequenced by using HiSeq 2000 from Illumina^112^.

#### Raw read and quality check

Sequence images were transformed with BaseCaller Illumina software to BCL files and demultiplexed to fastq files with bcl2fastq v2.20.0.422. Sequencing quality was determined using FastQC v. 0.11.5 software (http://www.bioinformatics.babraham.ac.uk/projects/fastqc/).

#### Mapping and normalization

Sequences were aligned to the reference genome *Homo sapiens* (hg38 version 96, https://www.ensembl.org/Homo_sapiens/Info/Index) using STAR aligner^113^ v. 2.5.2a, allowing for 2 mismatches within 50 bases. Subsequently, read counting was performed using featureCounts^114^ v. 1.5.0-p1. Read counts were analyzed in the R/Bioconductor environment v. 3.6.1 (www.bioconductor.org) using the DESeq2^115^ package v. 1.24.0. Candidate genes were filtered using a false detection rate (FDR)-corrected *p*-value threshold of 0.05. Genes were annotated using the *Homo sapiens* GTF file hg38 version 96 (https://www.ensembl.org/Homo_sapiens/Info/Index) to quantify the reads within genes.

#### Matrisome analysis

Matrisome analysis was performed by using repositories of ECM-related genes identified in the Matrisome project^116^. The human matrisome was uploaded from http://matrisomeproject.mit.edu/ and used as a reference for the analysis of collagens, glycoproteins, and ECM regulators. Heat maps for matrisome groups and PCA plots were created using QLUCORE Omics Explorer 3.5 software.

### Western blotting

PA samples of human patients and rat lung samples were powdered in liquid nitrogen, and tissue lysates were prepared in NP-40 buffer (300 mM NaCl, 100 mM Tris pH 8.0, 1% Triton X, 1 tablet protease inhibitor per 10 mL). Sample loading was normalized to protein content measured by a bicinchoninic acid assay (BCA Protein assay kit, Pierce, Rockford, IL), and 25 µg of protein was loaded for each sample on 8–10% SDS–PAGE gels. Plasma samples were diluted with RIPA and 6x Laemmli buffer in a ratio 1:50, and boiled at 95 °C for 5 min. For each sample, 10 µg of protein were loaded on 10–15% SDS-PAGE gels.

After electrophoresis, proteins were transferred to 0.2 µm nitrocellulose (1620112, Bio Rad) membranes. Protein transfer was controlled by membrane staining with Ponceau S Staining Solution (59803, Cell Signaling Technology). Membranes were blocked in 3% dried milk (8076.3, Roth) for 30 min at RT and then stained at 4 °C overnight with the following primary antibodies at a dilution of 1:1000: rabbit polyclonal anti-α-Elastin (Abcam, ab21607, 68 kDa; validated by manufacturer to react with human protein for use in WB, IHC), mouse monoclonal anti-Fibrillin-1 (Abcam, ab124334, clone 3H6, 350 kDa; validated by manufacturer to react with human protein for use in WB, IHC), rabbit monoclonal anti-Collagen-I (Abcam, ab138492, clone EPR7785, 250 kDa pro-collagen, and 139 kDa cleaved fragment; validated by manufacturer to react with human protein for use in WB and IHC, and by Xia et al. in rat ^117^), rabbit monoclonal anti-collagen V (Abcam, ab275881, clone EPR23762-54, 145 kDa full length and 32 kDa C-terminal cleaved peptide; validated by manufacturer to react with human and rat protein for use in WB), rabbit polyclonal anti-LOX (Thermo Fisher, PA1-46020, 52 kDa; validated by manufacturer to react with human and rat protein for use in WB, IHC, IF), mouse monoclonal anti-IL-6 (Abcam, ab9324, clone 1.2-2B11-2G10, 25 kDa monomer and 50 kDa dimer^118^; validated by manufacturer to react with human and rat protein for use in WB), rabbit polyclonal anti-IFN-γ (Invitrogen, PA5-95560, 22 kDa; validated by manufacturer to react with human and rat protein for use in WB, IHC), mouse monoclonal anti-MMP2 (Invitrogen, 436000, clone 101, 70 kDa proenzyme and 65 kD cleaved fragment that reflects the active form of MMP2; validated by manufacturer to react with human and rat protein for use in WB, IHC), rabbit monoclonal anti-MMP9 (Invitrogen, MA5-32705, clone JA80-73, 82 kDa; validated by manufacturer to react with human and rat protein for use in WB, IHC, IF), rabbit polyclonal anti-MMP12 (Abcam, ab128030, 54 kDa proenzyme, and 45 kDa and 29 kDa cleaved fragments, the latter reflects the active form of MMP12^119^; validated by manufacturer to react with human protein for use in IHC), rabbit polyclonal anti-MMP13 (Invitrogen, PA5-16566, 53 kDa proenzyme, and 48 kDa and 35 kDa cleaved fragments, the latter reflects the active form of MMP13^120^; validated by manufacturer to react with human and rat protein for use in WB, IHC), rabbit monoclonal anti-CD68 (Abcam, ab227458, clone EPR20545, 90 kDa; validated by manufacturer to react with human protein for use in WB, IHC), rabbit polyclonal anti-mast cell tryptase (McT) (Santa Cruz Biotechnology, sc-32889, 30 kDa monomer and 60 kDa dimer^121^; validated by manufacturer to react with human and rat protein for use in WB, IHC), rabbit monoclonal anti-TGF-β1 (Abcam, ab179695, clone EPR18163, 12 kDa monomer and 25 kDa dimer; validated by manufacturer to react with human and rat protein for use in WB), and mouse monoclonal anti-GAPDH (Abcam, ab8245, clone 6C5, 37 kDa; validated by manufacturer to react with human and rat protein for use in WB). Next, membranes were washed 3 times for 5 min each with TBST (20 mMol/L Tris-HCl [pH 7.4], 150 mMol/L NaCl, 0.1% Tween-20) and incubated for 1 h at room temperature with either of the following secondary antibodies at a dilution of 1:10,000: goat anti-rabbit HRP (sc-2004, Santa Cruz) or goat anti-mouse HRP (sc-2031, Santa Cruz). For detection of β-Actin as loading control horseradish peroxidase (HRP)-conjugated mouse monoclonal anti-β-Actin (Abcam, ab3280-500, clone mAbcam 8226, 45 kDa; validated by manufacturer to react with human and rat protein for use in WB) antibodies were used.

After washing with TBST 3 times, the immunoreactive bands were detected by a Celvin chemiluminescence and fluorescence imager and SnapAndGo softeware (Biostep). The quantification of protein bands was performed using Fiji-ImageJ-win64 v.2.11.0. software and signals were normalized to GAPDH, β-Actin, or Ponceau S as loading controls.

### Data analyses and statistics

σ_t_/ε_t_ and E/ε_t_ curves are shown as mean ± standard error of the mean (SEM) for human samples and as mean ± standard deviation (SD) for rat samples. All other data are presented either as box-and-whisker plots or violine plots. Statistical analyses and data visualization were performed using GraphPad Prism 8, OriginPro 8, Microsoft Excel 2016, and QLUCORE Omics Explorer 3.5, respectively.

For statistical comparison of the two groups, the two-tailed nonparametric Mann-Whitney U test and the Wilcoxon matched-pairs signed rank test were used for unpaired and paired samples, respectively. For multiple group comparisons, Kruskal-Wallis one-way analysis of variance (ANOVA) on ranks followed by comparison to the control group or pairwise multiple comparisons (Dunn’s test) was applied. The relationship between the two variables was evaluated by a two-tailed nonparametric Spearman’s rank correlation coefficient. All *p*-values for statistically significant differences (*p* < 0.05) are shown in figures (or corresponding figure legends or tables).

### Reporting summary

Further information on research design is available in the Nature Portfolio Reporting Summary linked to this article.

## Supplementary information

Supplementary Information

Description of Additional Supplementary File

Supplementary Video 1- Elastic fibers in donor pulmonary artery

Supplementary Video 2 - Elastic fibers in LHD w/o PH pulmonary artery

Supplementary Video 3- Elastic fibers in PH-LHD pulmonary artery

Reporting Summary

## Data Availability

Publicly available data of the human genome hg38 version 96 (https://www.ensembl.org/Homo_sapiens/Info/Index) and of the human matrisome (http://matrisomeproject.mit.edu/) were used. RNA-seq data of the human PA transcriptome generated in this study are deposited in Gene Expression Omnibus (GEO) data repository under GSE236251 number. All other data underlying or supporting the findings in this study are given in the main article and associated files. Source data are provided with this paper.

## Source data

Source Data

## Code availability

No original code was generated in this study.
