# Supplementary material for: Elastin stabilization prevents impaired biomechanics in human pulmonary arteries and pulmonary hypertension in rats with left heart disease
Source: Nat Commun. 2023 Jul 21;14:4416. doi: 10.1038/s41467-023-39934-z (PMC10362055; doi:10.1038/s41467-023-39934-z)
Supplement: Supplementary file 1 — Supplementary Information [file 41467_2023_39934_MOESM1_ESM.pdf]

Supplementary Material

Supplementary Tables

Suppl. Table 1. Correlation matrix

p-value	PAP sys	PAP dias	PAP mean	PCWP	TPG	CO	CI	PVR	E 0.5	σt 1MPa	Age
**PAP sys**		9.2054E-18	6.689E-25	1.7769E-16	0.00023095	0.05668168	0.00075945	0.00020576	0.00424887	0.03610612	0.05319756
**PAP dias**	9.2054E-18		2.8127E-26	1.7274E-14	8.8309E-06	0.19654905	0.0316959	5.8443E-06	0.00466381	0.01313668	0.13580809
**PAP mean**	6.689E-25	2.8127E-26		8.857E-18	1.249E-05	0.16425377	0.01136559	1.8709E-05	0.0056207	0.02144032	0.22959234
**PCWP**	1.7769E-16	1.7274E-14	8.857E-18		0.04585938	0.08392796	0.01007379	0.0092285	0.00430816	0.02330523	0.36904932
**TPG**	0.00023095	8.8309E-06	1.249E-05	0.04585938		0.71172626	0.96106241	2.6064E-08	0.12830844	0.12213188	0.51238498
**CO**	0.05668168	0.19654905	0.16425377	0.08392796	0.71172626		2.3624E-13	0.0337304	0.97936681	0.91268416	0.00720519
**CI**	0.00075945	0.0316959	0.01136559	0.01007379	0.96106241	2.3624E-13		0.02983893	0.42824919	0.61269661	0.19313284
**PVR**	0.00020576	5.8443E-06	1.8709E-05	0.0092285	2.6064E-08	0.0337304	0.02983893		0.44448533	0.28565155	0.34299387
**E 0.5**	0.00424887	0.00466381	0.0056207	0.00430816	0.12830844	0.97936681	0.42824919	0.44448533		3.4318E-31	0.20370881
**σt 1MPa**	0.03610612	0.01313668	0.02144032	0.02330523	0.12213188	0.91268416	0.61269661	0.28565155	3.4318E-31		0.29297683
**Age**	0.05319756	0.13580809	0.22959234	0.36904932	0.51238498	0.00720519	0.19313284	0.34299387	0.20370881	0.29297683	

Spearman r	PAP sys	PAP dias	PAP mean	PCWP	TPG	CO	CI	PVR	E 0.5	σt 1MPa	Age
**PAP sys**		0.88393001	0.94312025	0.90688885	0.53895924	-0.335009	-0.4838231	0.64582579	0.39757599	-0.3098753	0.27506929
**PAP dias**	0.88393001		0.95032493	0.87972523	0.62711241	-0.2306599	-0.3207498	0.74334884	0.3937882	-0.3630806	0.2139021
**PAP mean**	0.94312025	0.95032493		0.91619629	0.61889066	-0.2478855	-0.3740543	0.71550006	0.38606888	-0.3383771	0.17299593
**PCWP**	0.90688885	0.87972523	0.91619629		0.3098178	-0.3324185	-0.40727	0.48299595	0.43170254	-0.3672845	0.14218883
**TPG**	0.53895924	0.62711241	0.61889066	0.3098178		0.07307438	-0.0080803	0.83814954	0.23846198	-0.2551281	0.10395121
**CO**	-0.335009	-0.2306599	-0.2478855	-0.3324185	0.07307438		0.90950308	-0.4024659	-0.0046827	0.02018534	-0.4590552
**CI**	-0.4838231	-0.3207498	-0.3740543	-0.40727	-0.0080803	0.90950308		-0.4109184	-0.1210644	0.08041272	-0.1976379
**PVR**	0.64582579	0.74334884	0.71550006	0.48299595	0.83814954	-0.4024659	-0.4109184		0.15054058	-0.2131949	0.18611853
**E 0.5**	0.39757599	0.3937882	0.38606888	0.43170254	0.23846198	-0.0046827	-0.1210644	0.15054058		-0.976311	0.18285938
**σt 1MPa**	-0.3098753	-0.3630806	-0.3383771	-0.3672845	-0.2551281	0.02018534	0.08041272	-0.2131949	-0.976311		-0.1584301
**Age**	0.27506929	0.2139021	0.17299593	0.14218883	0.10395121	-0.4590552	-0.1976379	0.18611853	0.18285938	-0.1584301	

Sample size	PAP sys	PAP dias	PAP mean	PCWP	TPG	CO	CI	PVR	E 0.5	σt 1MPa	Age
**PAP sys**		50	50	42	42	33	45	28	50	46	50
**PAP dias**	50		50	42	42	33	45	28	50	46	50
**PAP mean**	50	50		42	42	33	45	28	50	46	50
**PCWP**	42	42	42		42	28	39	28	42	38	42
**TPG**	42	42	42	42		28	39	28	42	38	42
**CO**	33	33	33	28	28		33	28	33	32	33
**CI**	45	45	45	39	39	33		28	45	42	45
**PVR**	28	28	28	28	28	28	28		28	27	28
**E 0.5**	50	50	50	42	42	33	45	28		46	50
**σt 1MPa**	46	46	46	38	38	32	42	27	46		46
**Age**	50	50	50	42	42	33	45	28	50	46	

Right heart catheterization

PAP sys, systolic pulmonary arterial pressure
PAP dias, diastolic pulmonary arterial pressure
PAP mean, mean pulmonary arterial pressure
PCWP, pulmonary capilary wedge pressure
TPG, transpulmonary gradient
CO, cardiac output
CI, cardiac index
PVR, pulmonary vascular resistance

Uniaxial tensile test

E0.5, stiffness at 0.5 strain
εt 1MPa, true strain at 1MPa stiffness

Age, patient's age

Statistically significant association

Note, statistical analysis is not adjusted for multiple comparisons.

Suppl. Table 2. Patient characteristics

**Studied Group**	**Donor (n=33)**	**LHD w/o PH (n=41)**	**PH-LHD (n=49)**	**PAH (n=4)**
**Sex, female/male**	10/23	18/23	14/35	1/3
**Age (years); median (range)**	41 (16-72)	55 (19-65)	55 (31-65)	39 (15-54)
**Body weight (kg); median (range)**				
Female	67 (60-90)	70 (56-122)	65 (50-91)	74
Male	80 (65-110)	94 (69-117)	85 (55-127)	70 (55-76)
**Underlying diseases, n/%**				
ICM	-	10/24.4	16/32.6	-
NICM	-	31/75.6	33/67.3	-
DCM	-	27/65.9	31/63.3	-
HCM	-	1/2.4	1/2.0	-
NCC	-	1/2.4	-	-
GCM	-	1/2.4	-	-
VCM	-	-	1/2.0	-
Loeffler endocarditis	-	1/2.4	-	-
iPAH	-	-	-	3/75
***aPAH** (in connective tissue disease)*	-	-	-	1/25

n, number of patients; ICM, ischemic cardiomyopathy; NICM, nonischemic cardiomyopathy; DCM, dilatative cardiomyopathy; HCM, hypertrophic cardiomyopathy; NCC, noncompaction cardiomyopathy; GCM, giant cell myocarditis; VCM, valvular cardiomyopathy; iPAH, idiopathic pulmonary arterial hypertension; aPAH, associated arterial hypertension.

Suppl. Table 3. Patient pulmonary hemodynamics

Studied Group	LHD w/o PH	PH-LHD	PAH
CO, L/min	4.9±1.6	3.4±0.9	3.87±0.9
CI, L/m/m^2	2.4±0.6	2.0±0.4	2.0±0.6
PAP systolic, mmHg	25.0±7.6	42.0±6.1	90±25.3
PAP diastolic, mmHg	12.0±5.3	22.0±5.1	39±16
PAP mean, mmHg	17.0±4.9	30.0±4.8	56±21
PCWP, mmHg	10.0±4.0	21.0±5.1	9±8
TPG, mmHg	7.0±3.7	10.0±3.6	34±2
PVR, dyn×s×cm^{-5}	102.0±76.2	194.0±135.7	919±342

Data are presented as the median±standard deviation. CO, cardiac output; CI, cardiac index; PAP, pulmonary arterial pressure; PCWP, pulmonary capillary wedge pressure; TPG, transpulmonary gradient; PVR, pulmonary vascular resistance.

Supplementary Figures

Suppl. Figure 1. Assessment of biomechanical properties in PAs

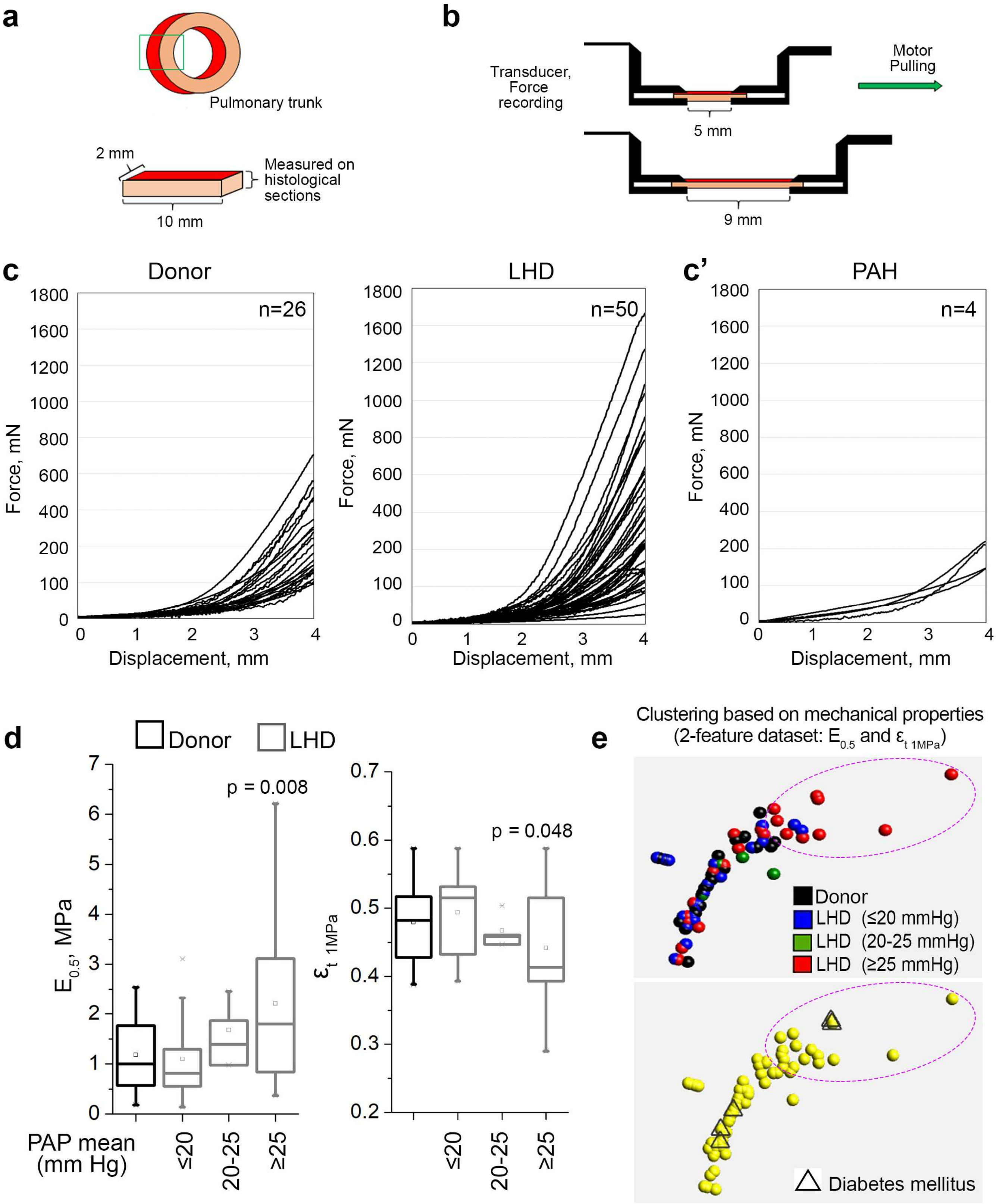

Suppl. Figure 1. a, Schematic illustration depicting the preparation of PA samples. Arrow points to the pulmonary trunk used for analysis, from which circumferential strips of 10 x 2 mm were prepared. **b**, Illustration of the experimental setup for uniaxial tensile testing of arterial biomechanical properties *ex vivo*. **c**, Force/displacement curves acquired by uniaxial tensile tests on PA samples obtained from donors, LHD patients, and PAH patients. **d**, Box-and-whisker plot graphs show $E_{0.5}$ in biologically independent PA samples from donors (n=26) and LHD patients with mean PAP ≤ 20 mmHg (n=24), mean PAP 20-25 mmHg (n=4), and mean PAP ≥ 25 mmHg (n=22), and $\varepsilon_{t\ 1MPa}$ in biologically independent PA samples from donors (n=22) and LHD patients with mean PAP ≤ 20 mmHg (n=20), mean PAP 20-25 mmHg (n=4), and mean PAP ≥ 25 mmHg (n=22) (subgroup analysis of dataset in Figure 1e). Samples of LHD patients with mean PAP ≥ 25 mmHg differ from donor controls with p=0.008 for $E_{0.5}$ and p=0.048 for $\varepsilon_{t\ 1MPa}$. **e**, Upper PCA plot generated based on a 2-feature biomechanical dataset ($E_{0.5}$ and $\varepsilon_{t\ 1MPa}$) shows that LHD samples with a mean PAP ≥ 25 mmHg (red) cluster apart from donor (black) and LHD samples with either mean PAP ≤ 20 mmHg (blue) or mean PAP 20-25 mmHg (green). Note that LHD samples with mean PAP 20-25 mmHg (green) are clustered together with samples with mean PAP ≤ 20 mm Hg (blue), and apart from stiff samples with mean PAP ≥ 25 mmHg (red). Lower PCA plot shows of same samples shows 5 patients with diabetes mellitus (triangle) that cluster with both stiff (n=2) and non-stiff (n=3) samples alike. Dashed circles outline stiffened PA samples.

Box-and-whisker plots (**d**) show mean (rectangle), median (line within the box), lower and upper 25% quartiles (limits of the box), 1.5 IQR (whiskers) and outliers (if applicable).

Statistics: **d,** Unpaired two-tailed Mann-Whitney U test (comparisons vs. donor).

Source data are provided as a Source Data file.

Suppl. Figure 2. PA ectasia in PH-LHD

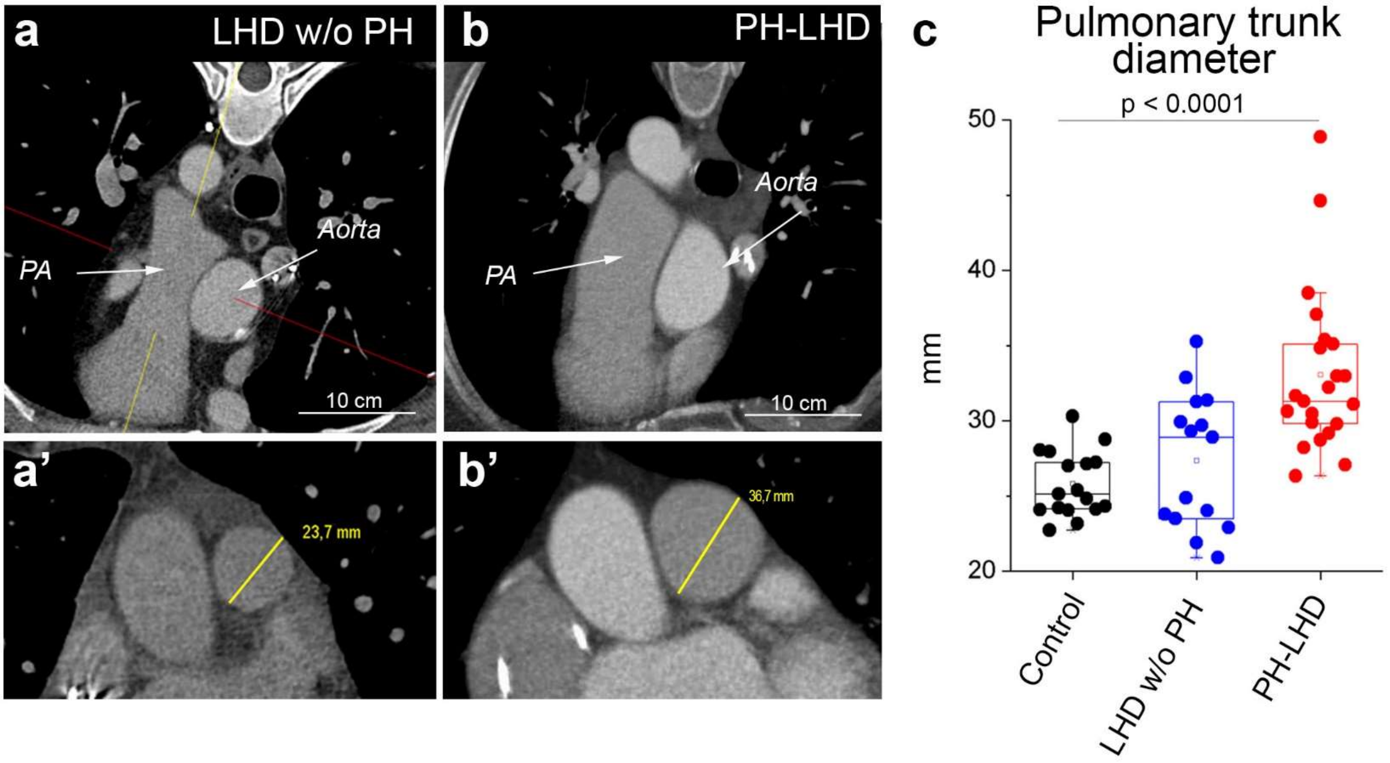

Suppl. Figure 2. a-b, Representative CT images show longitudinal (top) and cross-sectional (bottom) views of the PA trunk in an LHD w/o PH (left) and a PH-LHD (right) patient, respectively. PA diameter in **a'-b'** is given in yellow. **c**, Box-and-whisker plot shows pulmonary trunk diameter in control (n=17), LHD w/o PH (n=16), and PH-LHD (n=22) patients (p<0.0001 for PH-LHD vs. donor).

Box-and-whisker plots overlaid with dot plots (**c**) show individual data points, mean (rectangle), median (line within the box), lower and upper 25% quartiles (limits of the box), 1.5 IQR (whiskers) and outliers (if applicable). *Statistics:* **c**, Kruskal-Wallis one-way ANOVA on ranks followed by pairwise multiple comparison (Dunn's test). Source data are provided as a Source Data file.

Suppl. Figure 3. Impact of age and sex on pulmonary arterial stiffening

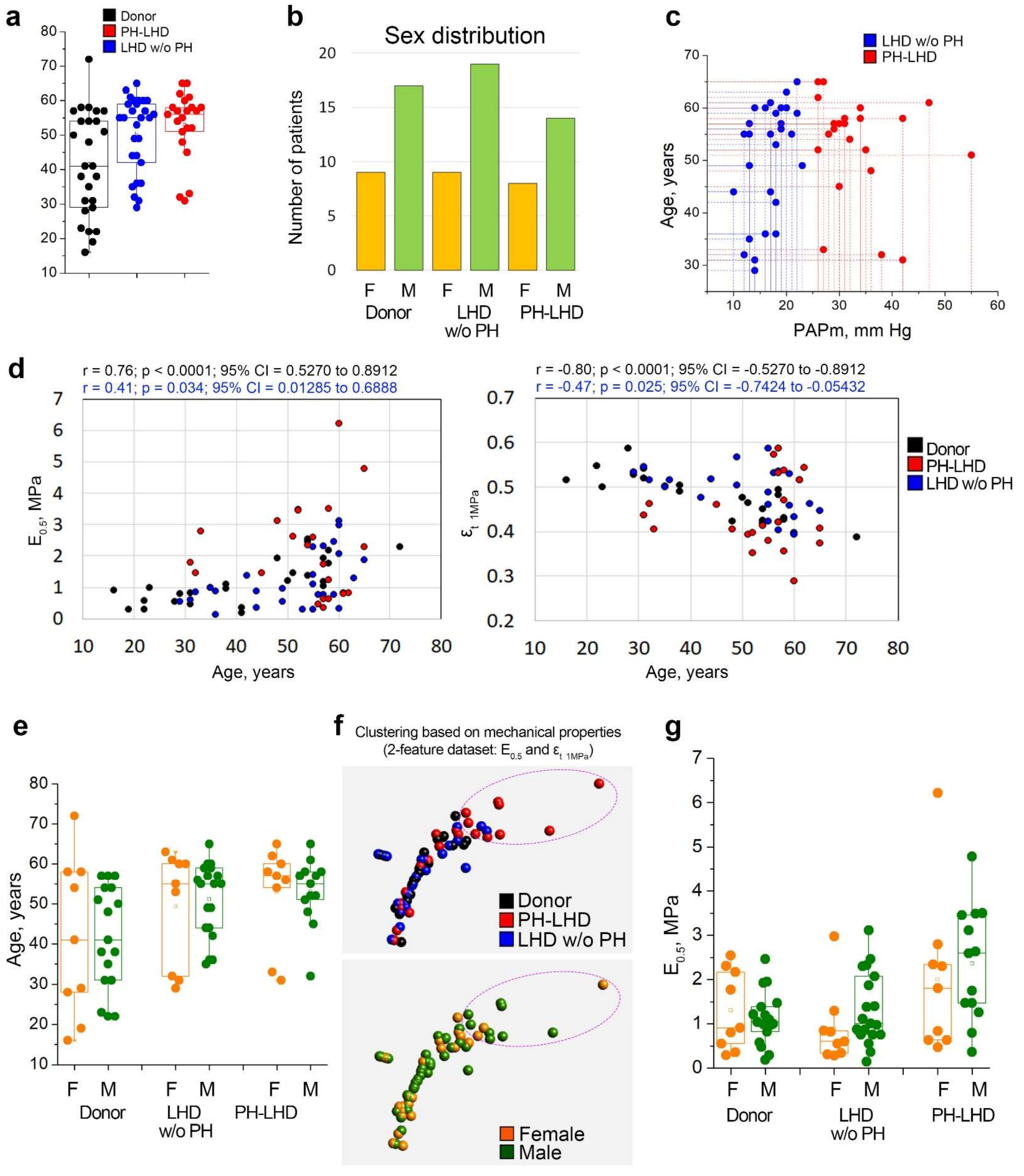

Suppl. Figure 3. a, Box-and-whisker plot shows the distribution of patient age in the donor (n=26), LHD w/o PH (n=28), and PH-LHD (n=22) groups. **b**, Bar graph shows the number of female (F) and male (M) patients in each group. **c**, Scatter diagram shows the relationship between patient age and mean PAP (PAPm). No significant correlation was detected by Spearman´s correlation analysis. **d**, Scatter plots show relationships between age and the arterial biomechanical parameters $E_{0.5}$ and $\varepsilon_{t\ 1MPa}$ in PAs of donors, LHD w/o PH patients, and PH-LHD patients. Biomechanical properties of PA samples correlate significantly with patient age in donor and LHD w/o PH cohorts, indicating a significant impact of physiological aging on PA stiffening. In the PH-LHD cohort, however, none of the PA biomechanical parameters correlated with age, highlighting the impact of PH-related remodeling rather than age in these patients. **e**, Box-and-whisker plot shows age distribution in female (F) and male (M) patients in studied groups. **f**, PCA plots generated based on $E_{0.5}$ and $\varepsilon_{t\ 1MPa}$ differentiated by study group (donor, LHD w/o PH, and PH-LHD) and by sex (F and M). PCA revealed that the majority of stiff PA samples (encircled area) were from male patients. **g**, Box-and-whisker plot shows $E_{0.5}$ differentiated for female (F) and male (M) sex in the PAs of donors, LHD w/o PH patients and PH-LHD patients. Compared to female samples, male samples showed a tendency for higher $E_{0.5}$, particularly in the PH-LHD group, but the difference did not reach significance.

Box-and-whisker plots overlaid with dot plots (**a,e,g**) show individual data points, mean (rectangle), median (line within the box), lower and upper 25% quartiles (limits of the box), 1.5 IQR (whiskers) and outliers (if applicable). *Statistics:* **c-d,** Spearman's coefficient of correlation Rho (r) and corresponding two-tailed statistics; 95% CI, r- and p-values are given. Note: statistical analyses (**d**) are not adjusted for multiple comparisons, **g**, Unpaired two-tailed Mann-Whitney U test.

Source data are provided as a Source Data file.

Suppl. Figure 4. Differential expression of genes coding for collagens

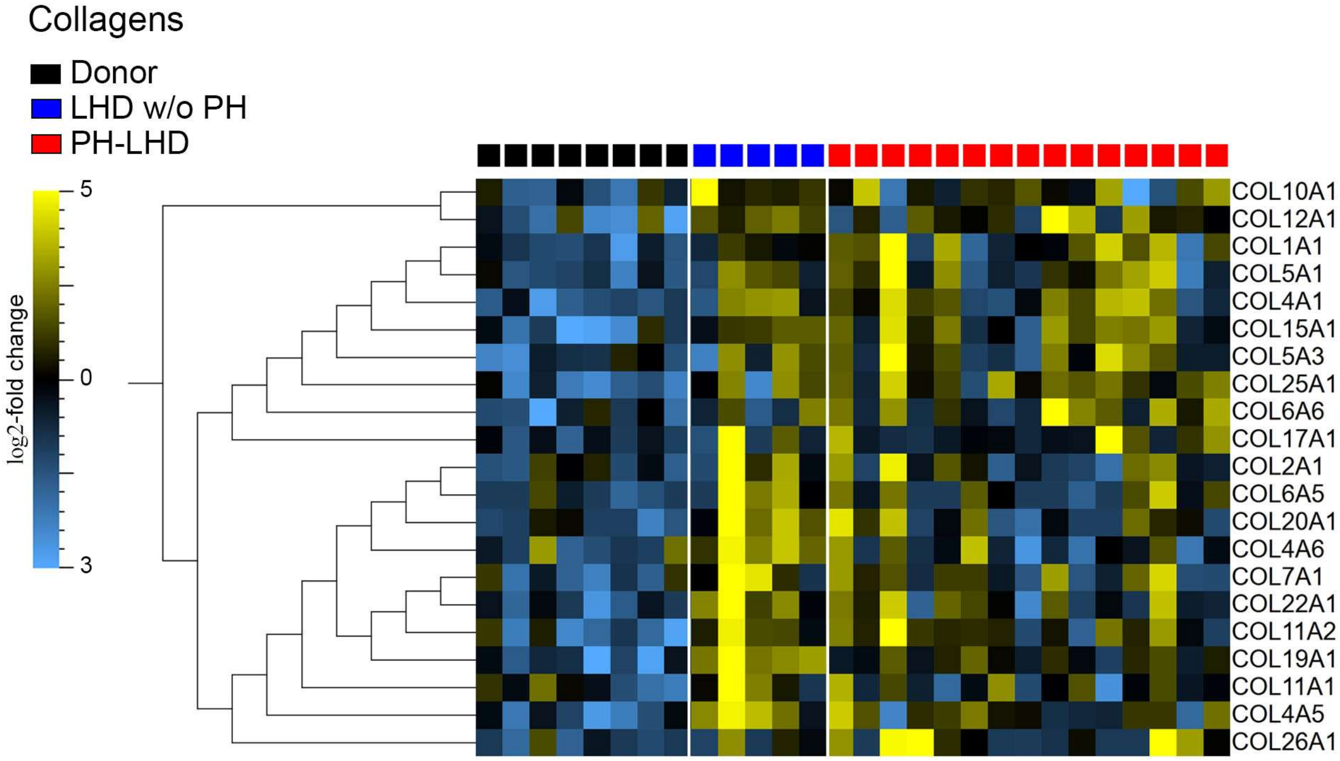

Suppl. Figure 4. Heat map shows log2-fold change in expression for differentially expressed genes coding for collagen types in PA samples of LHD w/o PH and PH-LHD patients relative to donor samples.

Statistics: Please see Source Data file corresponding to Suppl. Figure 4.

Suppl. Figure 5. Differential expression of genes coding for glycoproteins

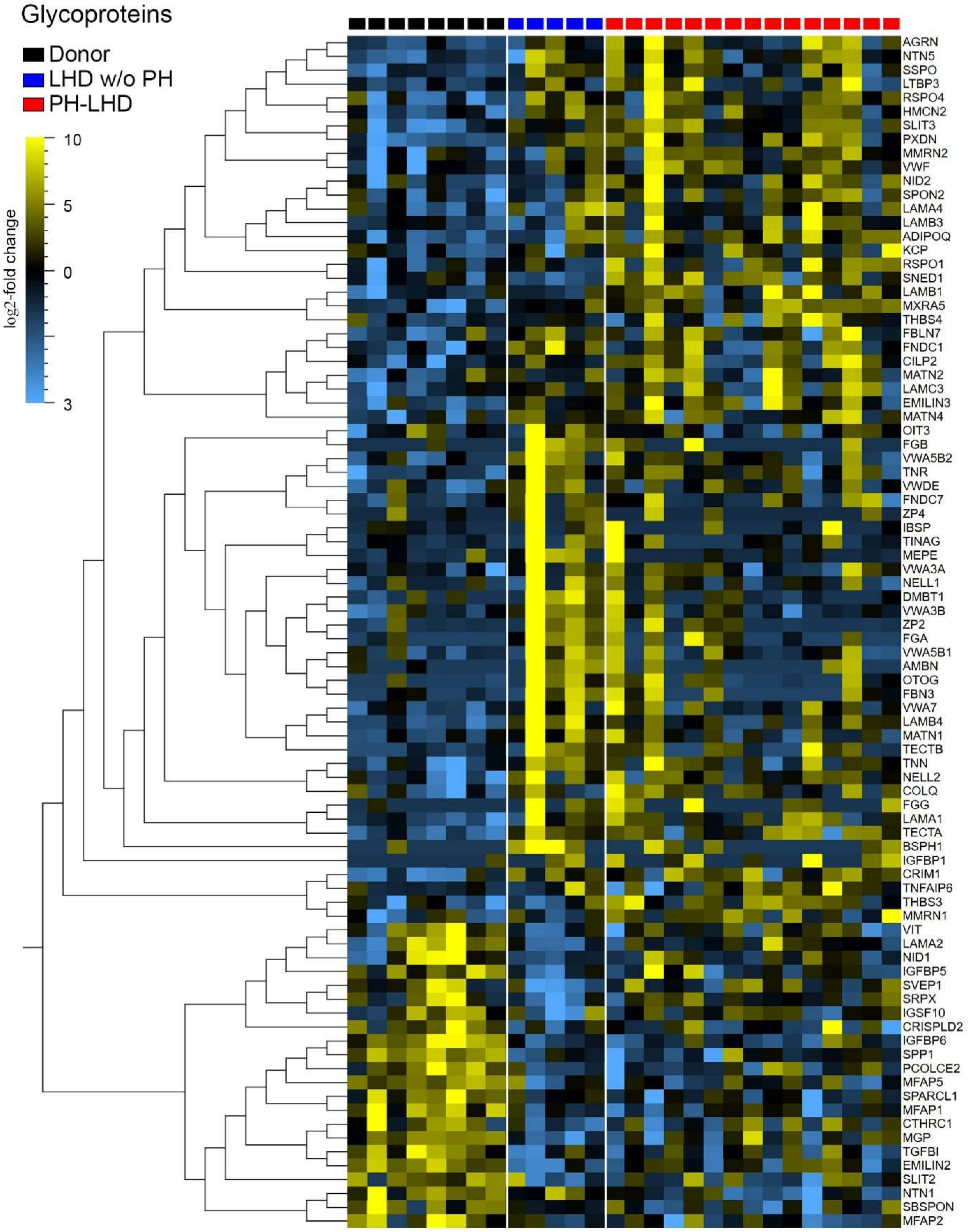

Suppl. Figure 5. Heat map shows log2-fold change in expression for differentially expressed genes coding for glycoproteins in PA samples of LHD w/o PH and PH-LHD patients relative to donor samples.

Statistics: Please see Source Data file corresponding to Suppl. Figure 5.

Suppl. Figure 6. Differential expression of genes coding for proteins that regulate the ECM

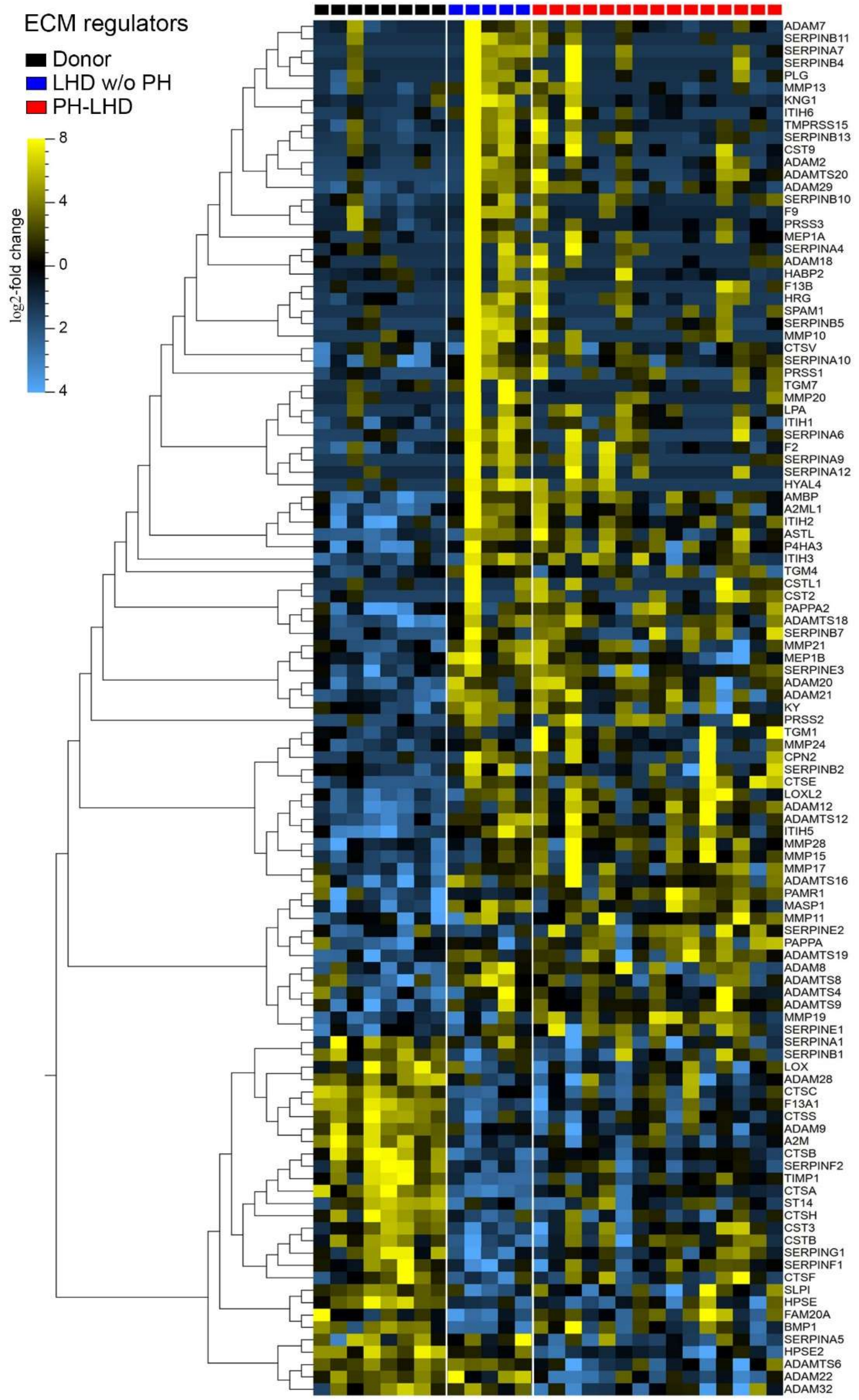

Suppl. Figure 6. The heat map shows the log2-fold change in expression for differentially expressed genes coding for proteins that regulate ECM formation, composition, and degradation.

Statistics: Please see Source Data file corresponding to Suppl. Figure 6.

Suppl. Figure 7. Ultrastructure and composition of elastic fibers

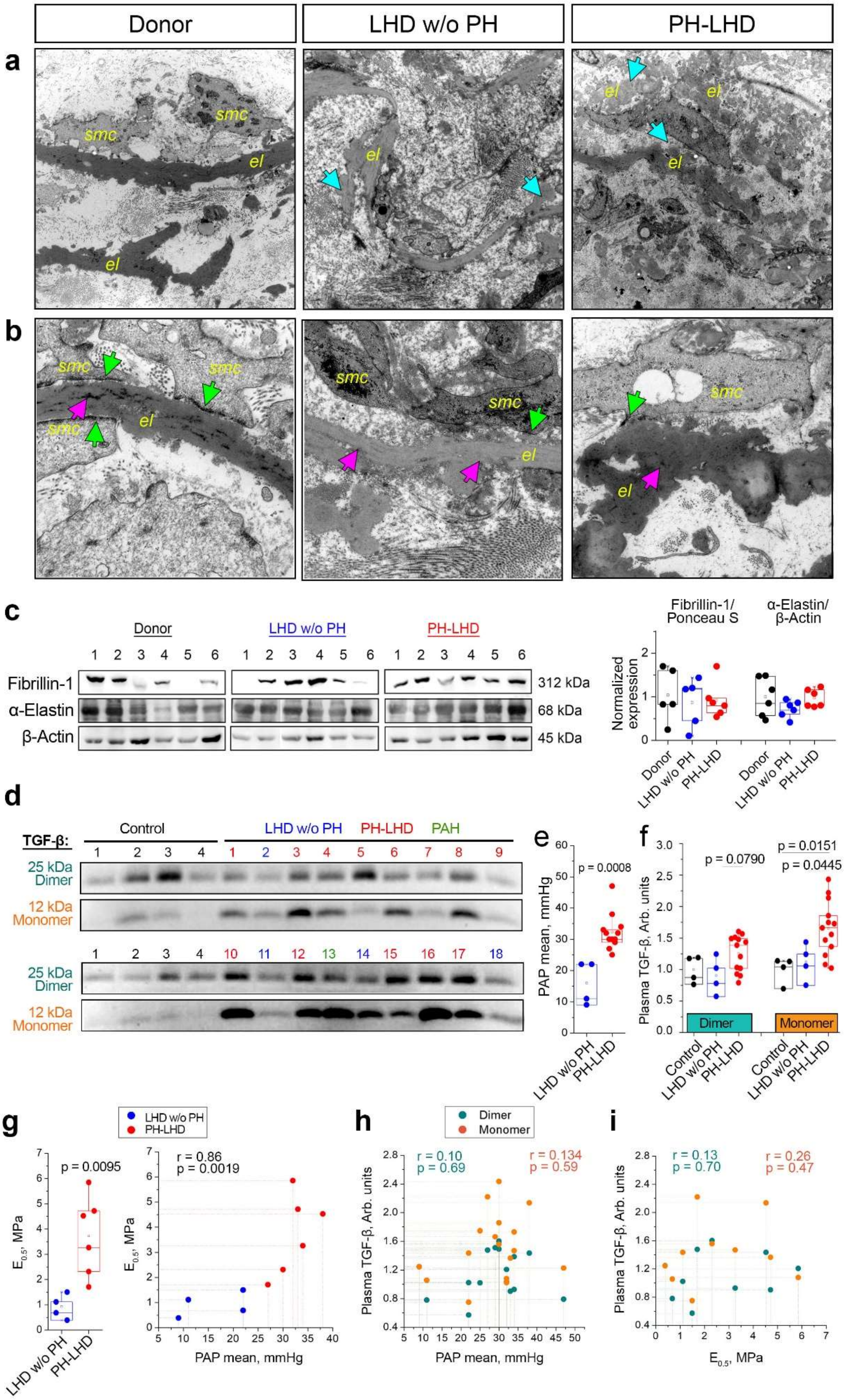

Suppl. Figure 7. a-b, TEM images show PA media in donor, an LHD w/o PH patient and a PH-LHD samples. *Smc*, smooth muscle cell; *el*, elastic fibers; arrows in blue - fragmented elastic fibers, green – attachment sites of SMCs on elastic fibers, pink - elastin core. Phenotype reproduced in n=2 biologically independent PA samples in each study group. **c,** Western blots and quantitative densitometric data (box-and-whisker plot) show expression of α-Elastin and Fibrillin-1 in biologically independent PA samples from donors (n=6), LHD w/o PH (n=6), or PH-LHD (n=6) patients. Box-and-whisker plots show quantitative densitometric data of Fibrillin-1 normalized to Ponceau S and of α-Elastin normalized to β-Actin. **d**, Western blots show levels of mature TGF-β (25 kDa dimer and 12 kDa monomer) detected in plasma of four healthy control subjects (three males aged 44, 54, and 39 years, and one 36-year-old female), one PAH patient (sample No 13, excluded from further analyses in **e-i**), four LHD w/o PH patients (samples No 2, 11, 14, 18), and thirteen PH-LHD patients (samples No 1, 3-10, 12, 15-17). **e**, Box-and-whisker plot shows mean PAP in LHD w/o PH (n=4) and PH-LHD (n=13) patients analyzed for plasma TGF-β levels in **d**. **f**, Quantitative densitometric data (box-and-whisker plots) show levels of TGF-β dimer and monomer in plasma of control (n=4), LHD w/o PH (n=4) and PH-LHD (n=13) patients normalized to the mean of the control group. **g**, Box-and-whisker plot shows PA stiffness parameter $E_{0.5}$ in LHD w/o PH (n=4) and PH-LHD (n=6) patients analyzed for plasma TGF-β levels (p=0.0095). Scatter plots show relationships between $E_{0.5}$ and mean PAP (n=10 patients, r=0.86; p=0.0019). **h-i**, Scatter plots show relationships between mean PAP and TGF-β (n=17 patients, r=0.10; p=0.69), and between $E_{0.5}$ and TGF-β (n=10 patients, r=0.14; p=0.59), respectively. Phenotype reproduced in n=2 biologically independent PA samples in each study group.

Box-and-whisker plots overlaid with dot plots (**c,e-g**) show individual data points, mean (rectangle), median (line within the box), lower and upper 25% quartiles (limits of the box), 1.5 IQR (whiskers) and outliers (if applicable). *Statistics:* **c,e-g,** Unpaired two-tailed Mann-Whitney U test. Spearman's coefficient of correlation Rho (r) and corresponding two-tailed statistics. Statistical analysis (**h,i**) is not adjusted for multiple comparisons.

Source data are provided as a Source Data file. Full scan blots are provided in Supplementary Figure 12.

Suppl. Figure 8. Ultrastructure and crosslinking of collagen fibers

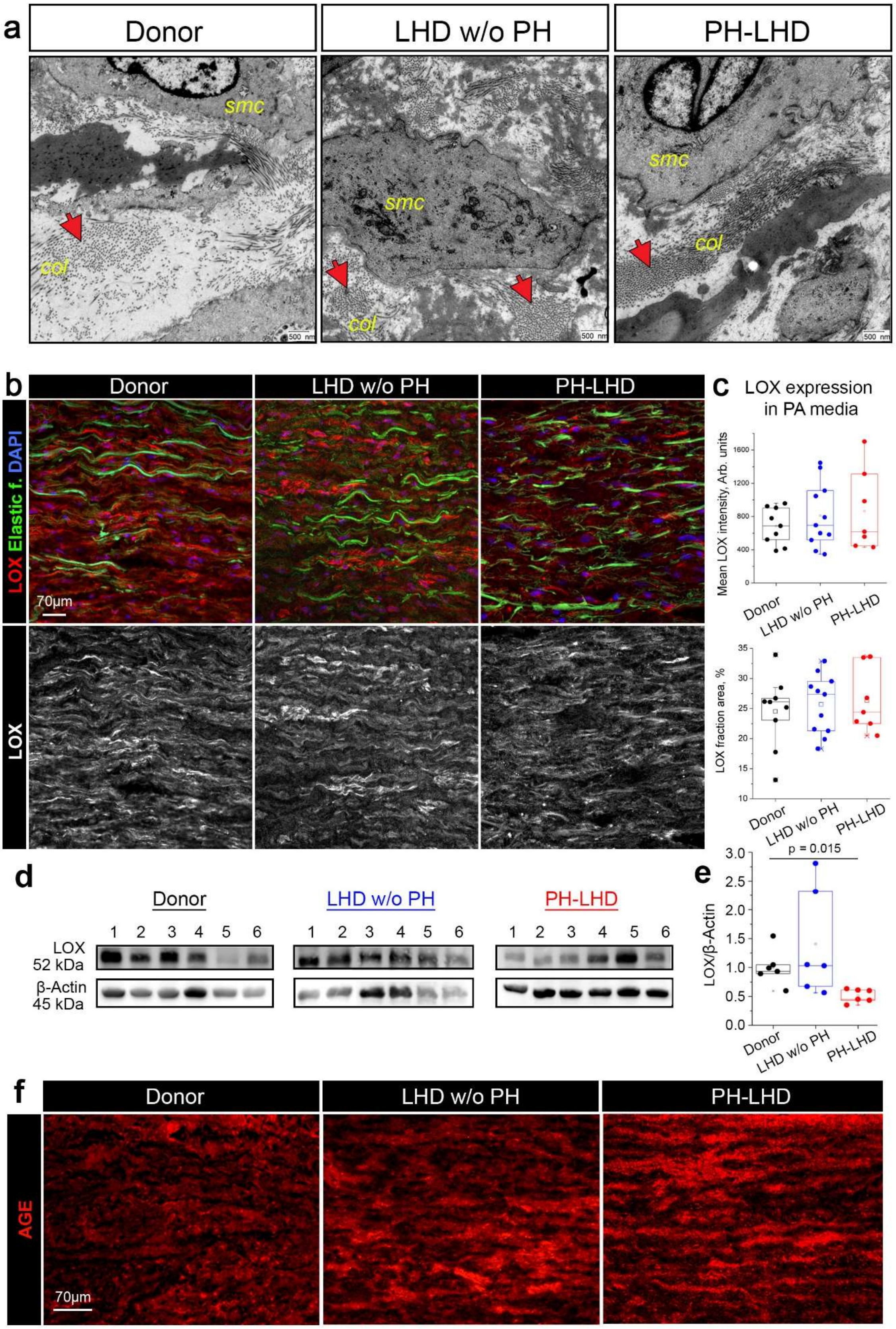

Suppl. Figure 8. a, TEM images show PA media in a sample from a donor, an LHD w/o PH patient and a PH-LHD patient. *Smc*, smooth muscle cell; *col,* collagen; red arrows show collagen fibrils clustered into fibers. Note the dense clustering of collagen fibrils in the PH-LHD sample. Phenotype reproduced in n=2 biologically independent PA samples in each study group. **b**, Representative confocal images of the PA media in samples from donors, LHD w/o PH patients and PH-LHD patients immunostained with anti-LOX (red), elastic fibers detected by autofluorescence (green) and nuclei detected by DAPI (blue). **c**, Box-and-whisker plots show the LOX intensity and LOX-positive area normalized to the image area in biologically independent PA samples from donors (n=9), LHD w/o PH (n=11), and PH-LHD (n=7) patients. **d-e**, Western blot and quantitative densitometric data (box-and-whisker plot) show LOX protein expression in biologically independent PA samples from donors (n=6), LHD w/o PH (n=6), and PH-LHD (n=6) patients. **f**, Representative images of the PA media immunostained with anti-AGE in samples from donors, LHD w/o PH and PH-LHD patients. Phenotype reproduced in biologically independent PA samples from donors (n=10), LHD w/o PH (n=8), and PH-LHD (n=8) patients.

Box-and-whisker plots overlaid with dot plots (**c,e-g**) show individual data points, mean (rectangle), median (line within the box), lower and upper 25% quartiles (limits of the box), 1.5 IQR (whiskers) and outliers (if applicable).

Statistics: **c,e**, Unpaired two-tailed Mann-Whitney U test.

Source data are provided as a Source Data file. Full scan blots are provided in Supplementary Figure 12.

Suppl. Figure 9. ***Ex vivo*** **stabilization of PA elastin by PGG**

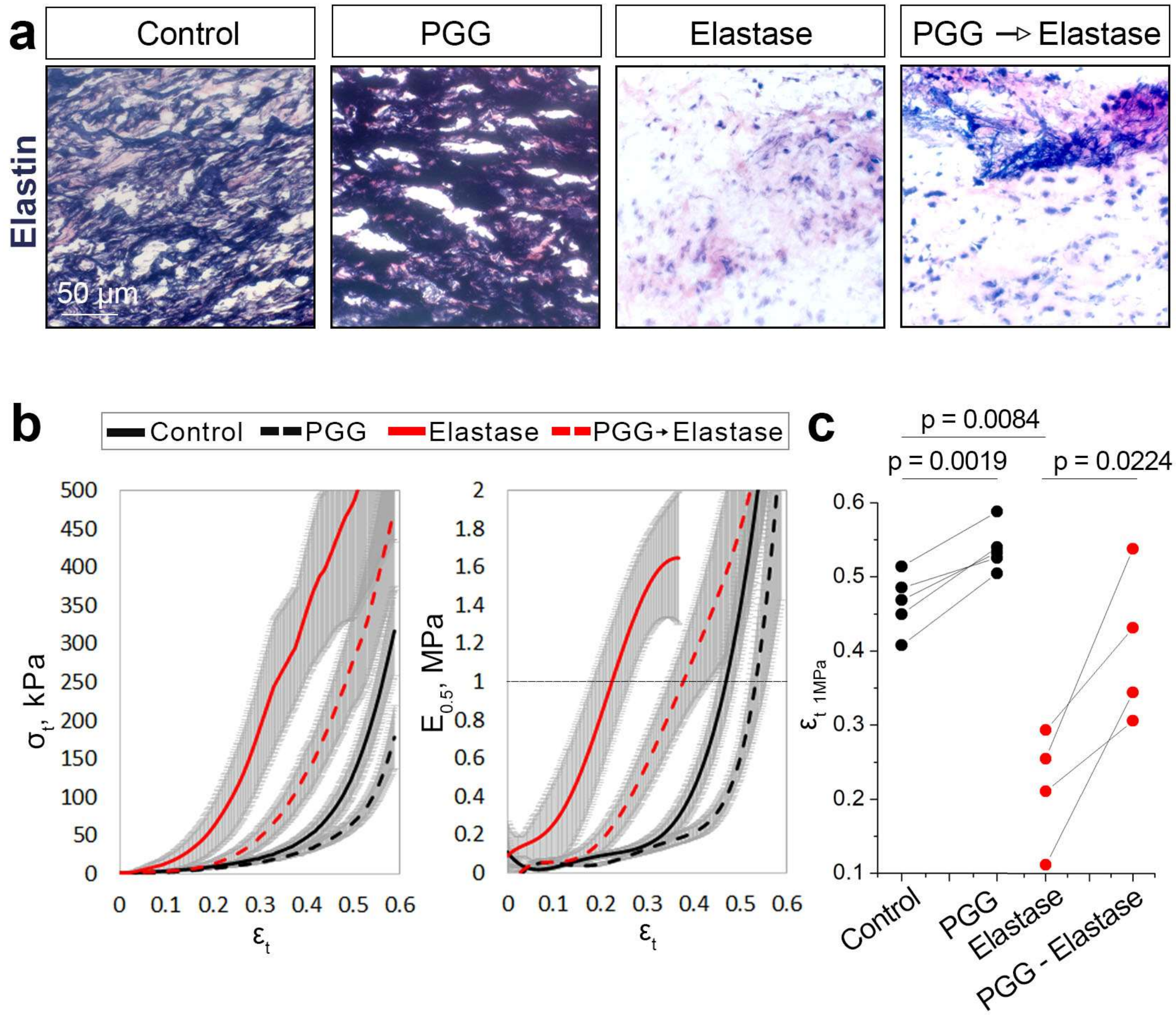

Suppl. Figure 9. a, Representative images show EVG stained human PA as naïve control, following treatment with PGG, elastase, or PGG followed by elastase. Note increased elastin content upon PGG application in naïve and elastase-treated samples. **b**, Group data show σ_t/ε_t and corresponding E/ε_t curves (mean±SEM) for untreated control (n=5), PGG-treated (n=5), elastase-treated (n=4), or PGG-followed-by-elastase treated (n=4) biologically independent donor PA samples. PGG treatment reduced PA stiffness as indicated by a right shift of both σ_t/ε_t and E/ε_t curves for both naïve and elastase-treated samples. **c**, Line graphs show significant increase in $\varepsilon_{t\ 1MPa}$ upon PGG application in naïve (n=5; p=0.0019) and elastase-treated (n=4; p=0.0224) biologically independent samples.

Statistics: Paired two-tailed Student's t-test.

Source data are provided as a Source Data file.

Suppl. Figure 10. PGG and Dox treatment reduces thickness of PA wall in AoB rats

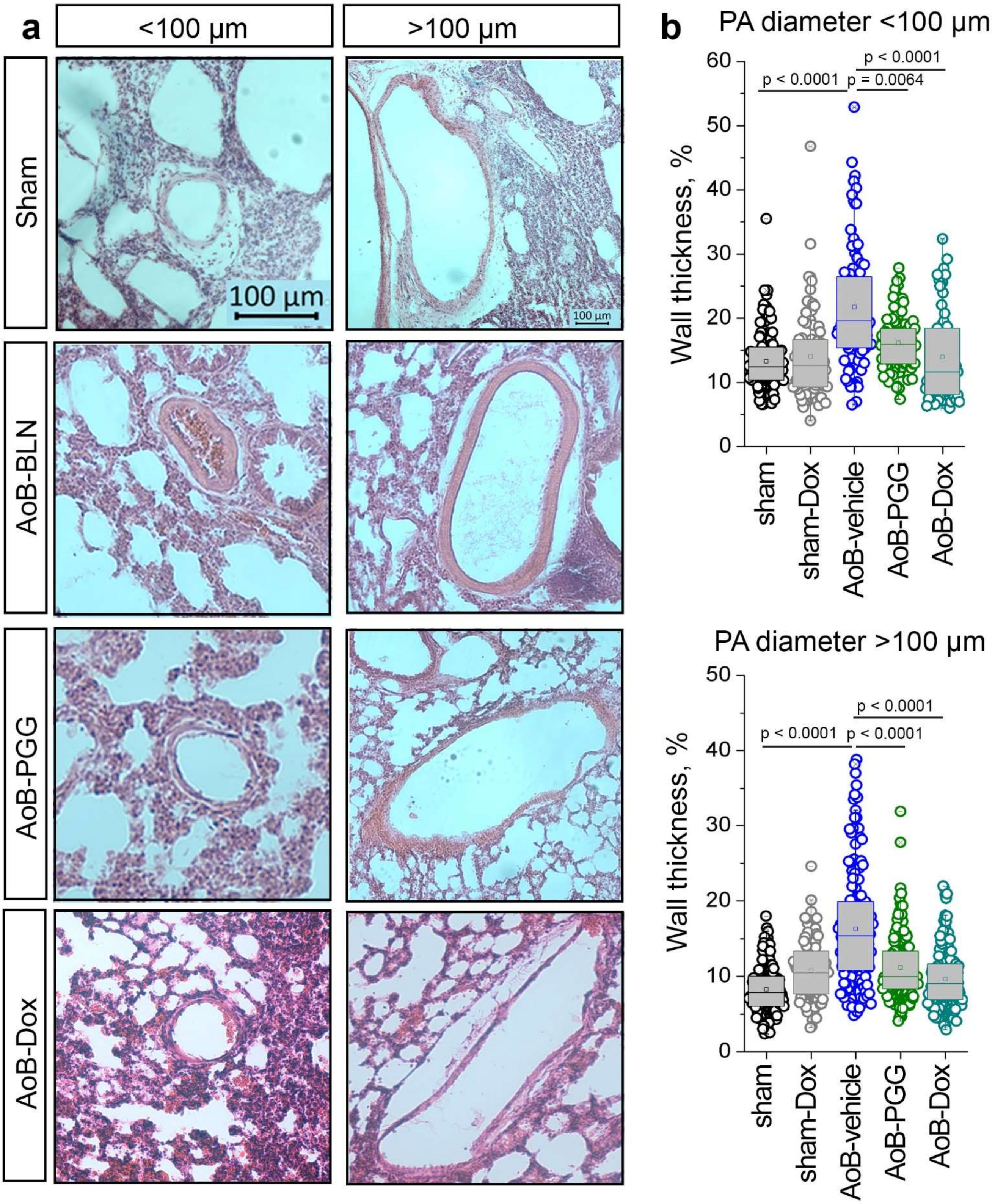

Suppl. Figure 10. a, Representative images of < 100 µm and > 100 µm diameter PAs visualized by HE staining in lungs of sham, AoB-vehicle, AoB-PGG, and AoB-Dox rats. **b**, Box-and-whisker plot shows wall thickness (quantified relative to arterial diameter in %) in PAs of < 100 µm and > 100 µm diameter. Individual dots represent individual arteries in sham (n=5), sham-Dox (n=4), AoB-vehicle (n=5), AoB-PGG (n=5), and AoB-Dox (n=4) rats. Vascular wall thickness differs between study groups as follows: in < 100 µm arteries ($p<0.0001$ for AoB-vehicle vs. sham, $p<0.0001$ for AoB-Dox vs. AoB-vehicle, $p=0.0064$ for AoB-PGG vs. AoB-vehicle) and in > 100 µm arteries ($p<0.0001$ for AoB-vehicle vs. sham, $p<0.0001$ for AoB-Dox vs. AoB-vehicle, $p<0.0001$ for AoB-PGG vs. AoB-vehicle).

Box-and-whisker plots overlaid with dot plots (**b**) show individual data points, mean (rectangle), median (line within the box), lower and upper 25% quartiles (limits of the box), 1.5 IQR (whiskers) and outliers (if applicable). *Statistics:* Kruskal-Wallis one-way ANOVA on ranks followed by pairwise multiple comparisons (Dunn's test). Source data are provided as a Source Data file.

Suppl. Figure 11. Dox attenuates PA stiffening and PH in a rat model of PH-LHD

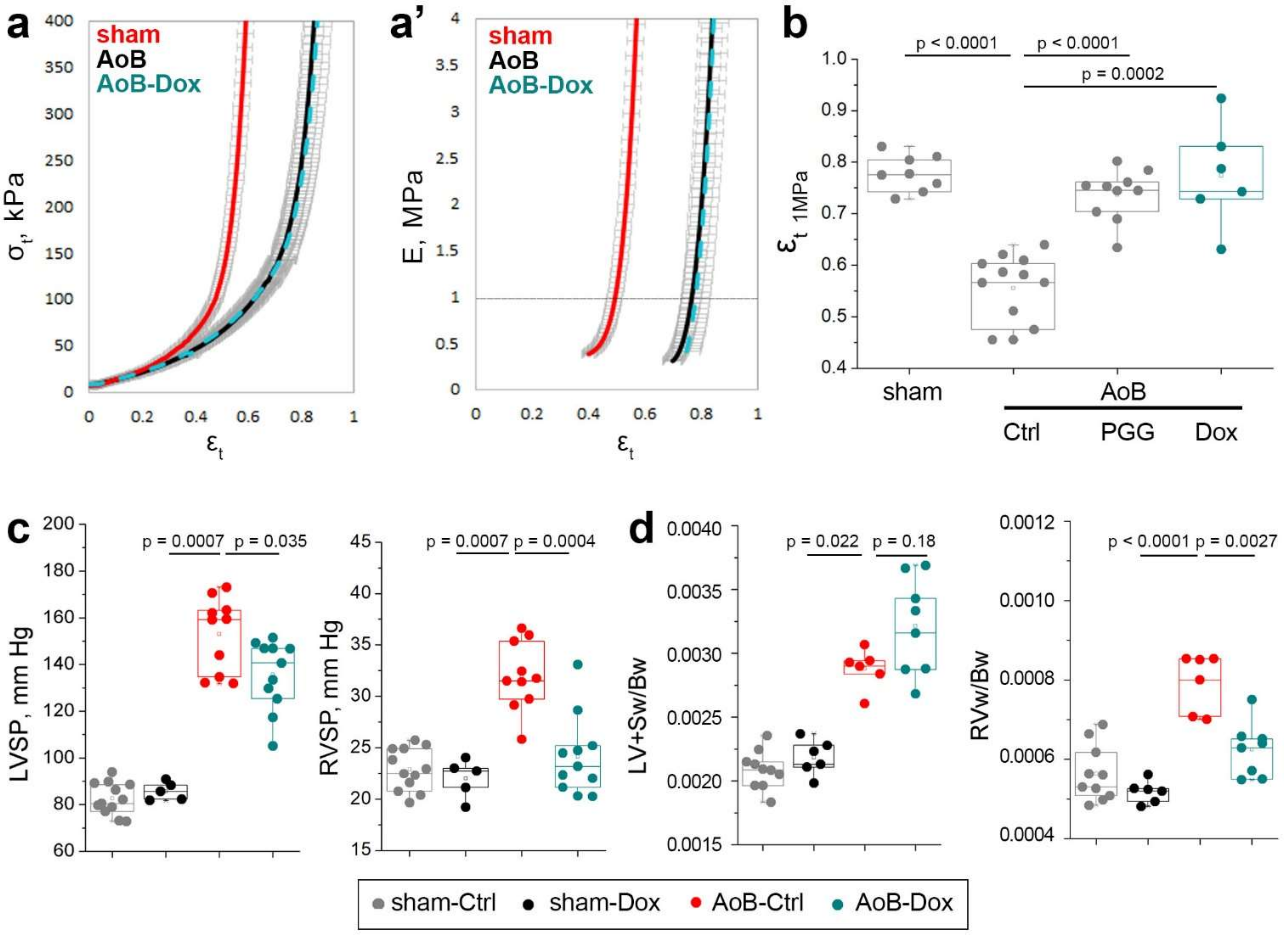

Suppl. Figure 11. a-a', Group data show σ_t/ε_t curves (mean±SD) and corresponding E/ε_t curves (mean±SD) for PAs of sham (n=8), AoB (n=12), and AoB-Dox (n=6) rats at 5 weeks after surgery. Dashed line indicates 1 MPa stiffness for which corresponding strain ($\varepsilon_{t\ 1MPa}$) is reported. **b**, Box-and-whisker plot shows effect of Dox treatment on $\varepsilon_{t\ 1MPa}$ (p=0.0002 for AoB-Dox vs AoB-Ctrl) as compared to PGG treatment (datasets for sham, AoB-Ctrl, and AoB-PGG are replicated from Figure 5f). **c**, Box-and-whisker plots show left (LVSP) and right (RVSP) ventricular systolic pressures assessed by cardiac catheterization in sham-Ctrl (n=12), sham-Dox (n=5), AoB-Ctrl (n=10), and AoB-Dox (n=11) rats. Hemodynamic parameters differ between groups as follows: LVSP (p=0.0007 for AoB-Ctrl vs. sham-Dox, p=0.035 for AoB-Dox vs. AoB-Ctrl) and RVSP (p=0.0007 for AoB-Ctrl vs. sham-Dox, p=0.0004 for AoB-Dox vs. AoB-Ctrl). **d**, Box-and-whisker plots show left (LV+Sw/Bw) and right (RVw/Bw) ventricular weights normalized to body weight in sham-Ctrl (n=10), sham-Dox (n=6), AoB-Ctrl (n=6), and AoB-Dox (n=8) rats. Cardiac weight differs between groups as follows: LV+Sw/Bw (p=0.022 for AoB-Ctrl vs. sham-Dox, p=0.18 for AoB-Dox vs. AoB-Ctrl) and RVw/Bw (p<0.0001 for AoB-Ctrl vs. sham-Dox, p=0.002 for AoB-Dox vs. AoB-Ctrl).

Statistics: Unpaired two-tailed Mann-Whitney U test.

Supplementary Figure 12. Original blots presented in figures

Figures to which blots correspond are indicated. Blots that were re-probed after stripping are listed under the same blot number. Red rectangles show the region presented in the respective figures.

Original blots corresponding to **Figure 2d**

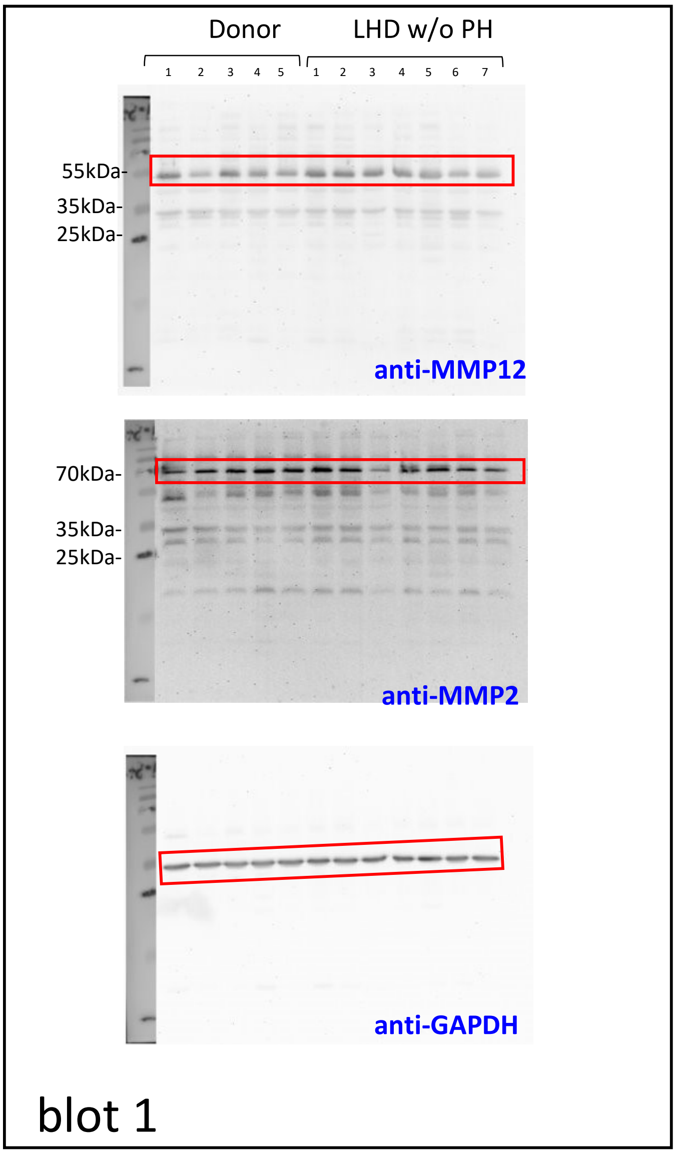

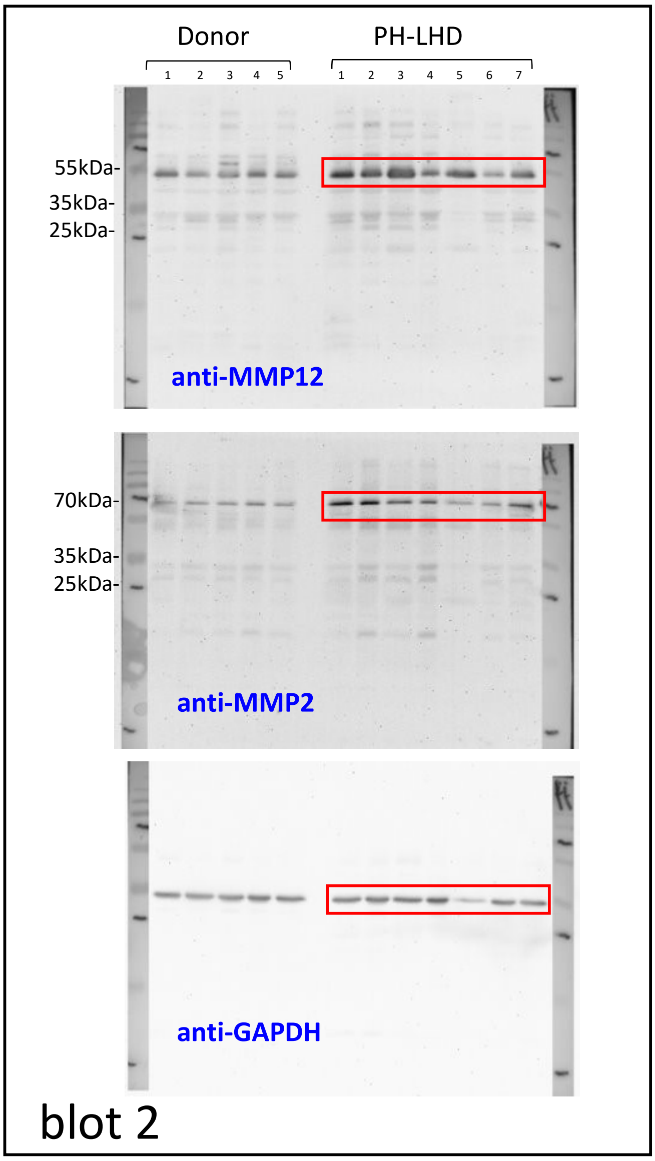

Figure 12 (continuation).

Original blots corresponding to **Figure 2d**

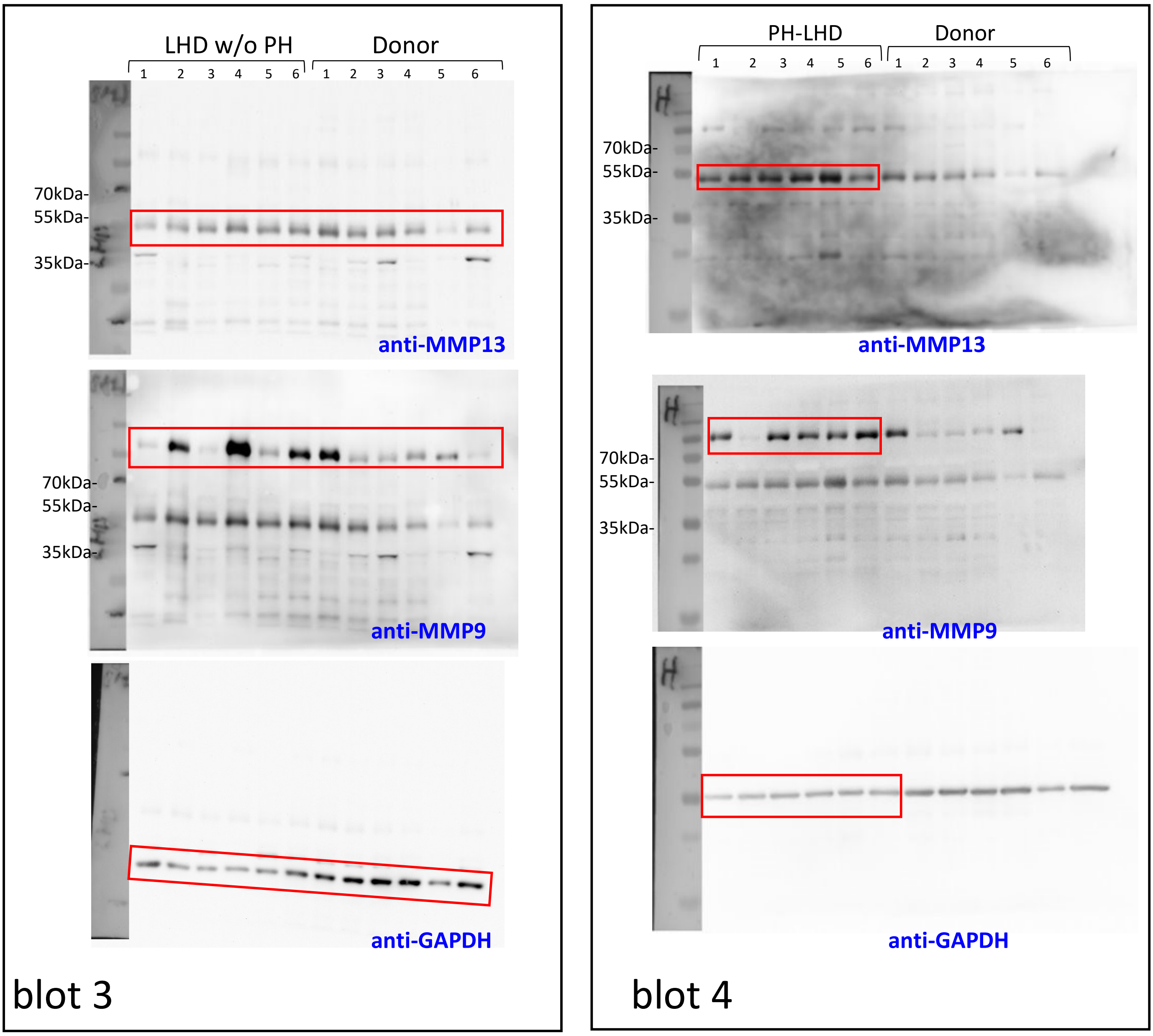

Figure 12 (continuation).

Original blots corresponding to **Figure 4c‘**

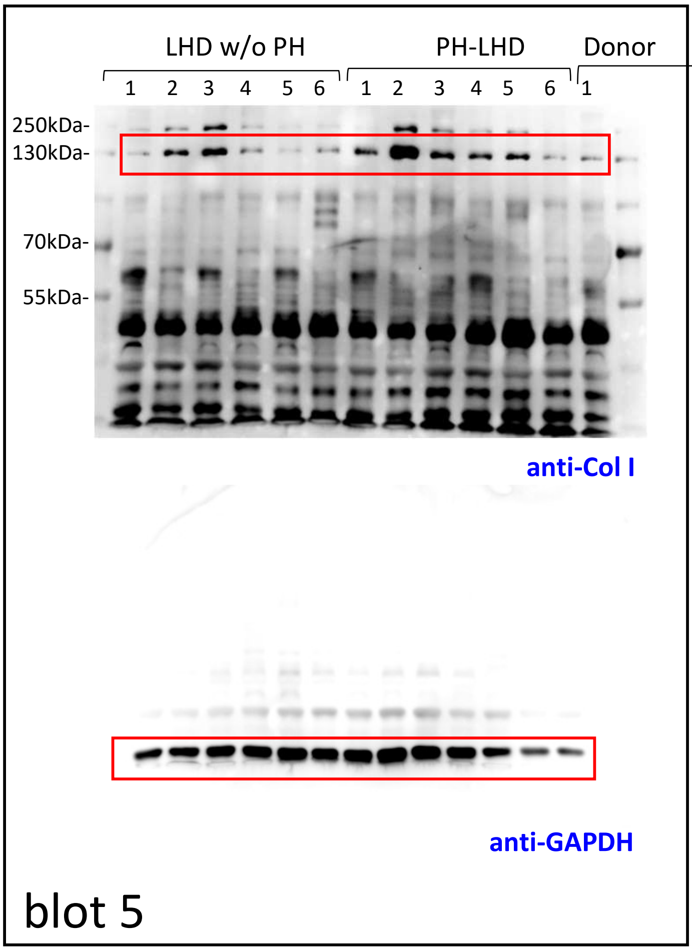

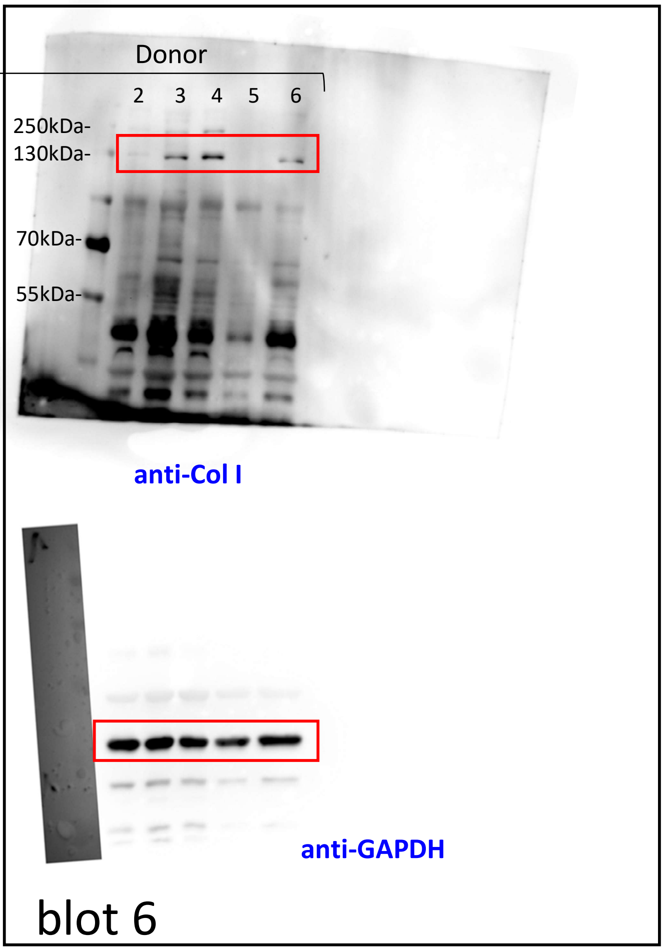

Note: these gels were prepared and processed in parallel

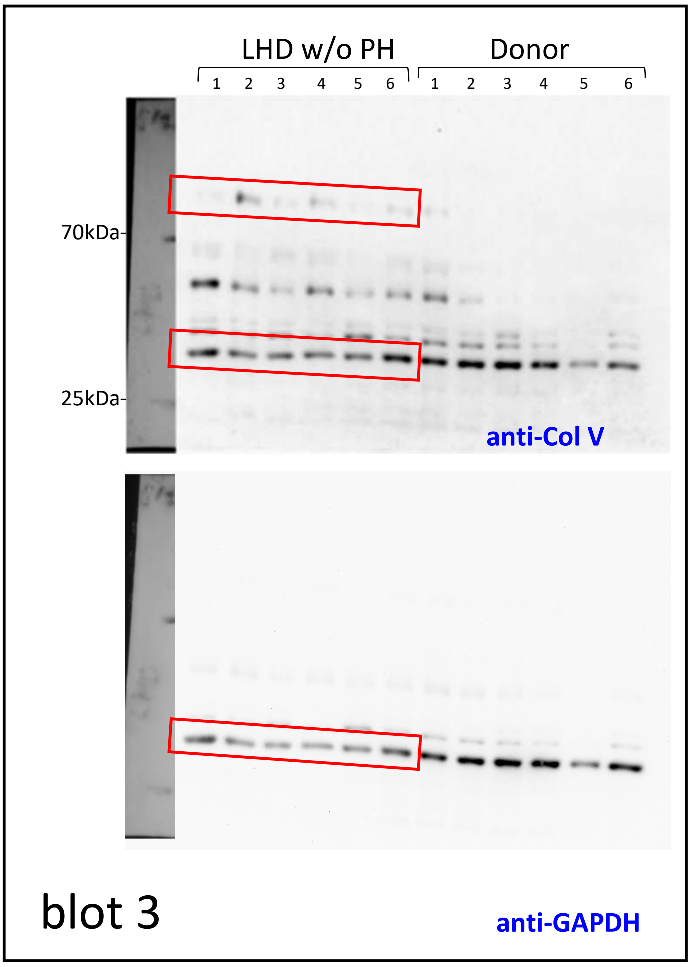

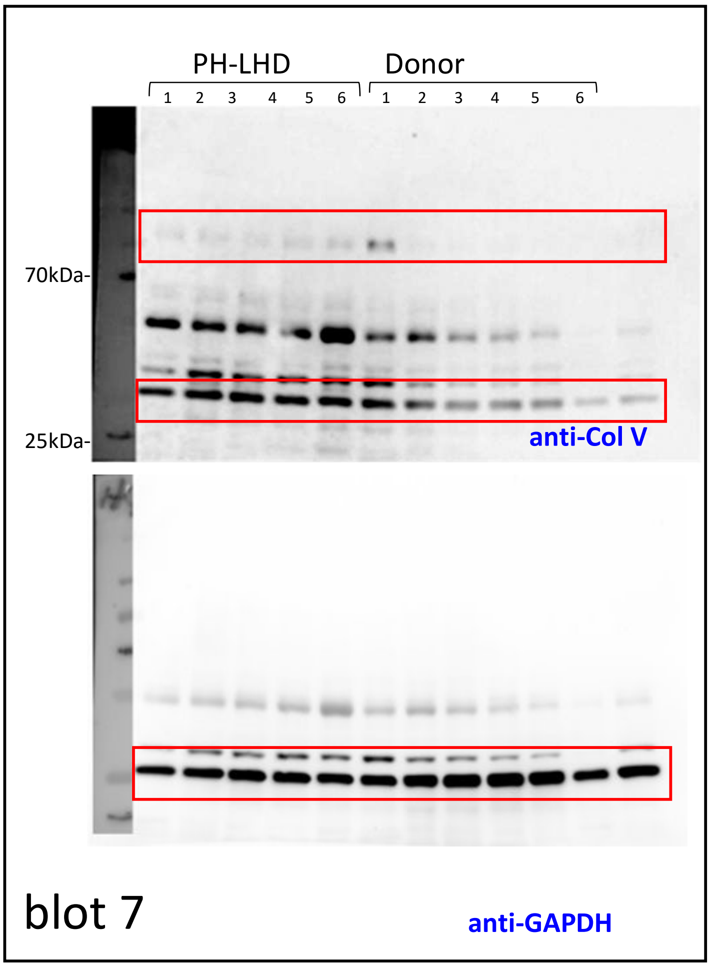

Figure 12 (continuation).

Original blots corresponding to **Figure 7a**

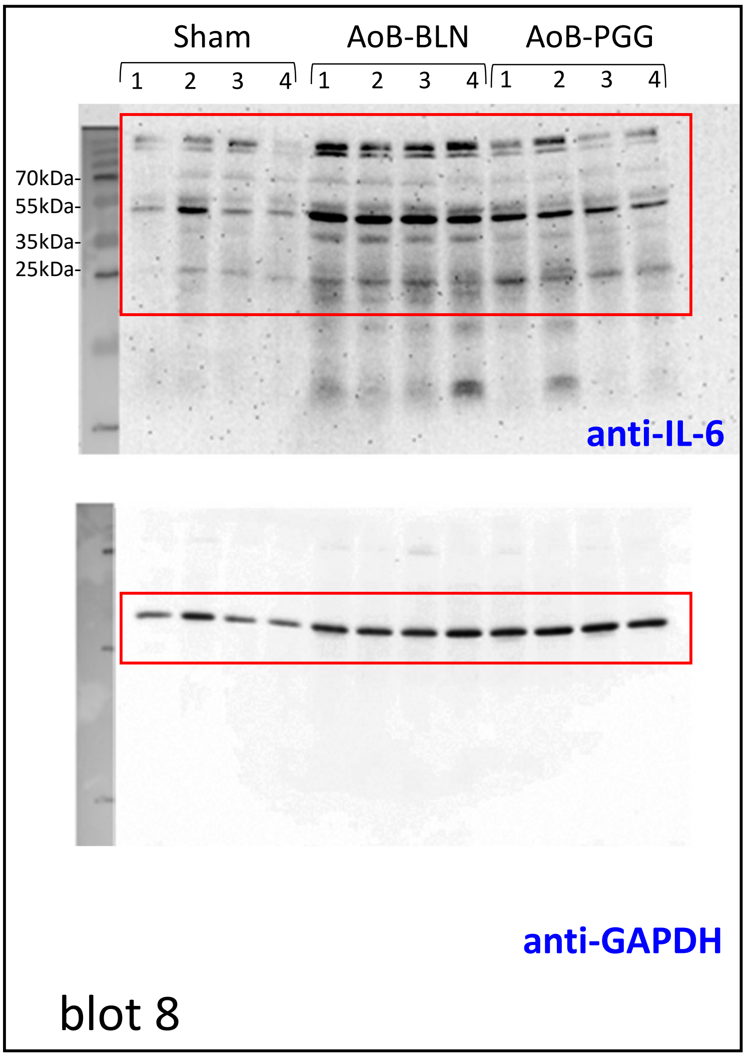

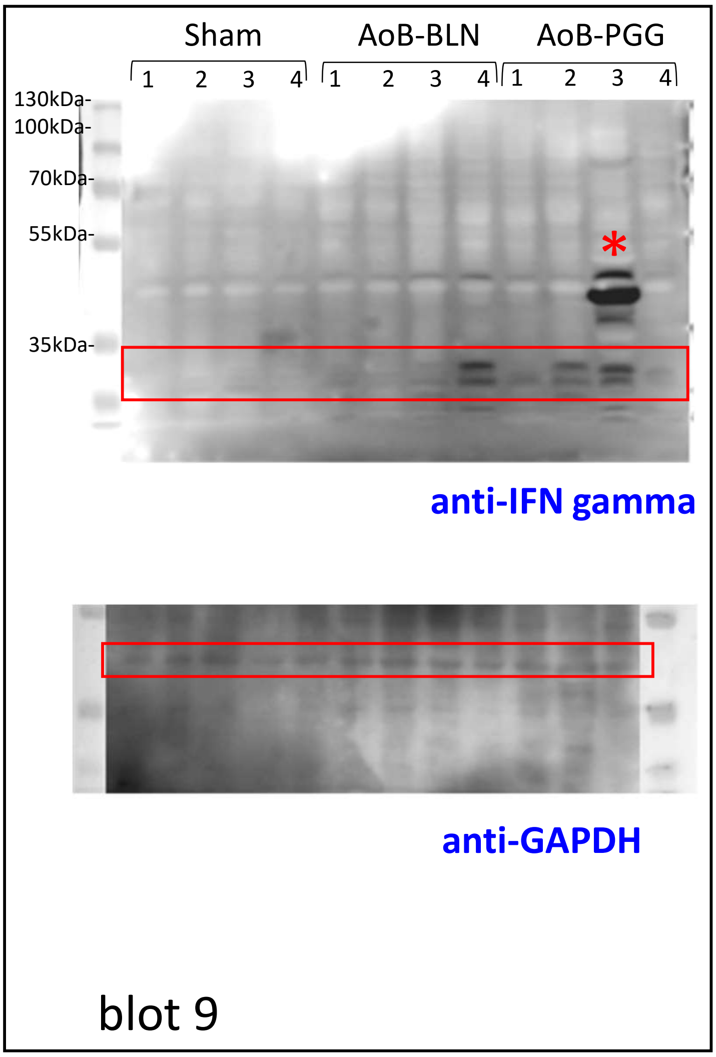

*sample excluded from quantification

Figure 12 (continuation).

Original blots corresponding to **Figure 7c**

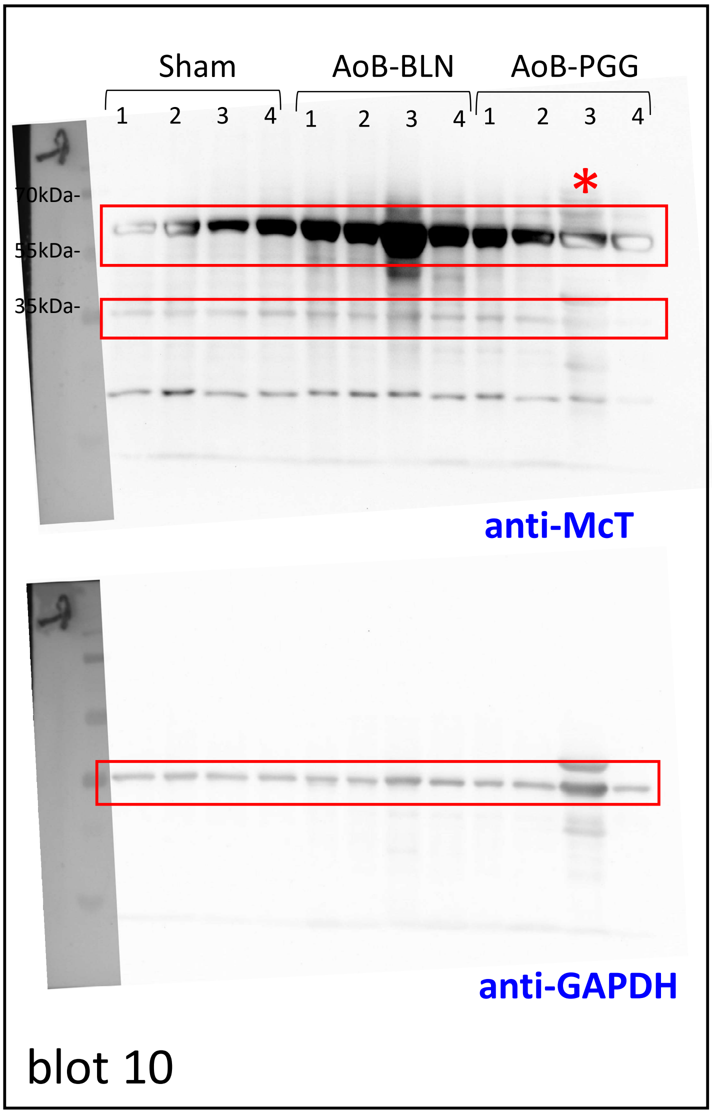

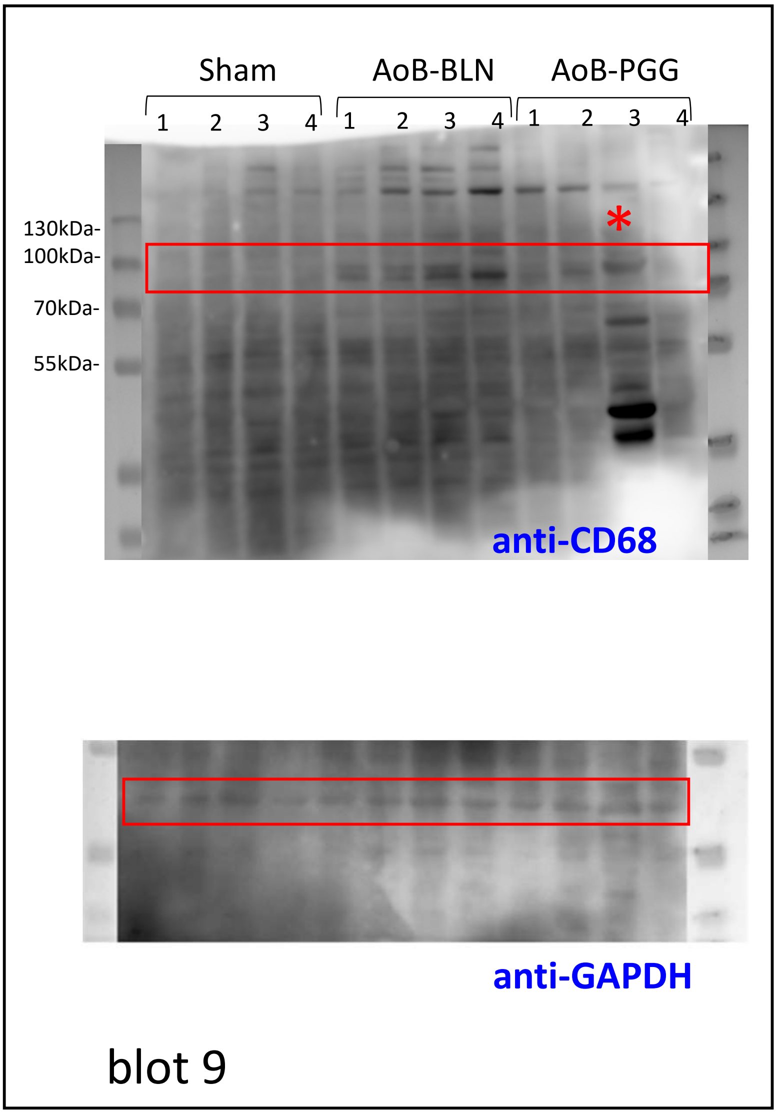

*sample excluded from quantification

Figure 12 (continuation).

Original blots corresponding to **Figure 7g**

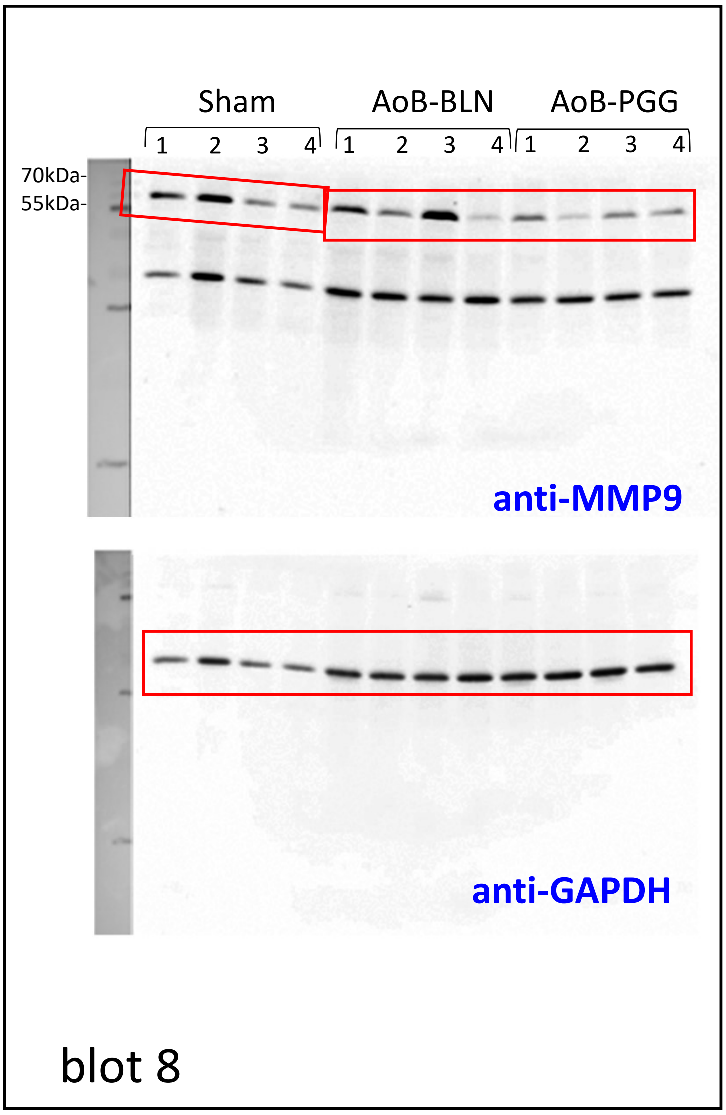

Original blots corresponding to **Figure 7i**

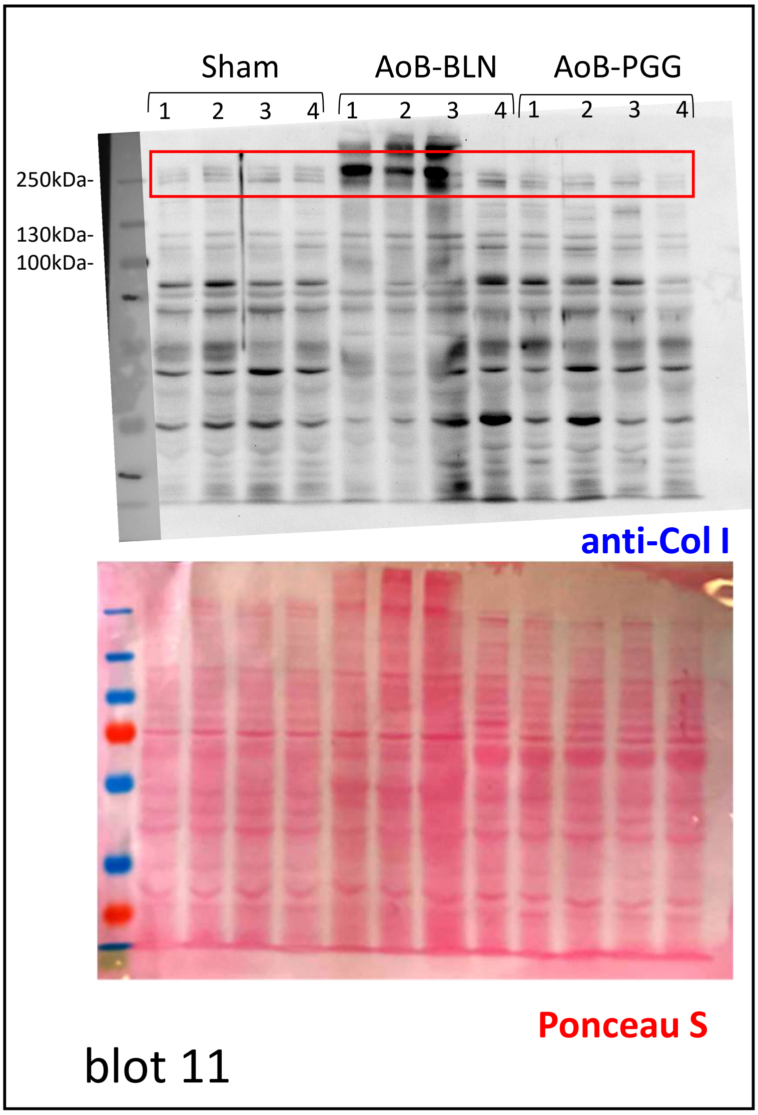

Figure 12 (continuation).

Original blots corresponding to **Supplementary Figure 7c**

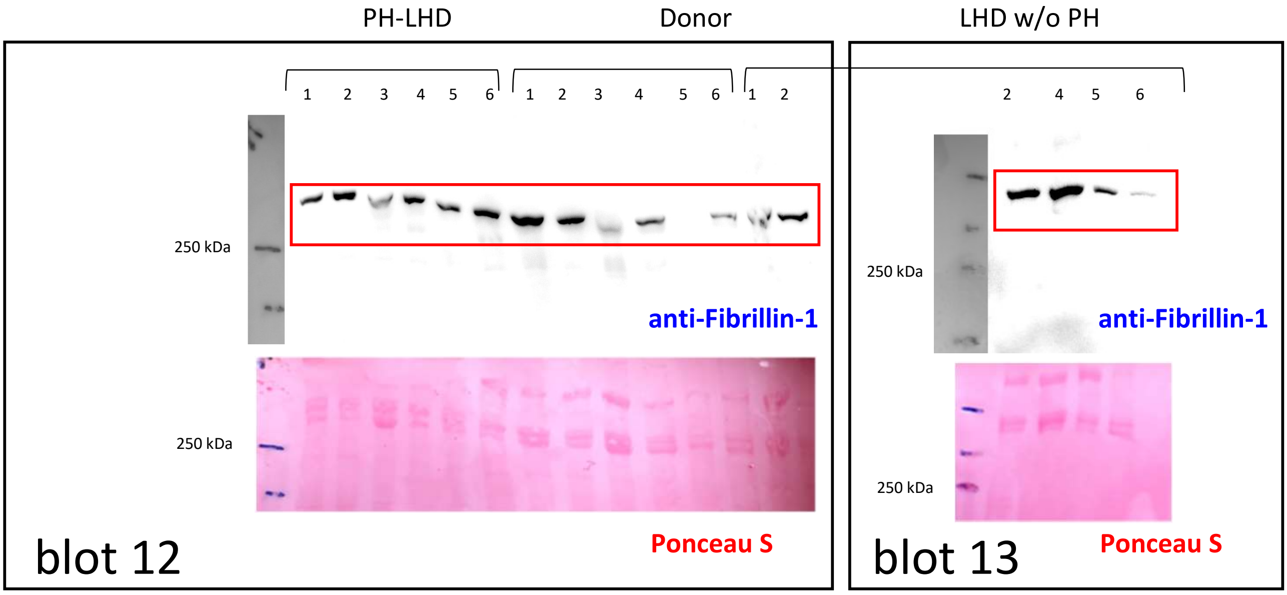

Note: gels were prepared, run and processed in parallel

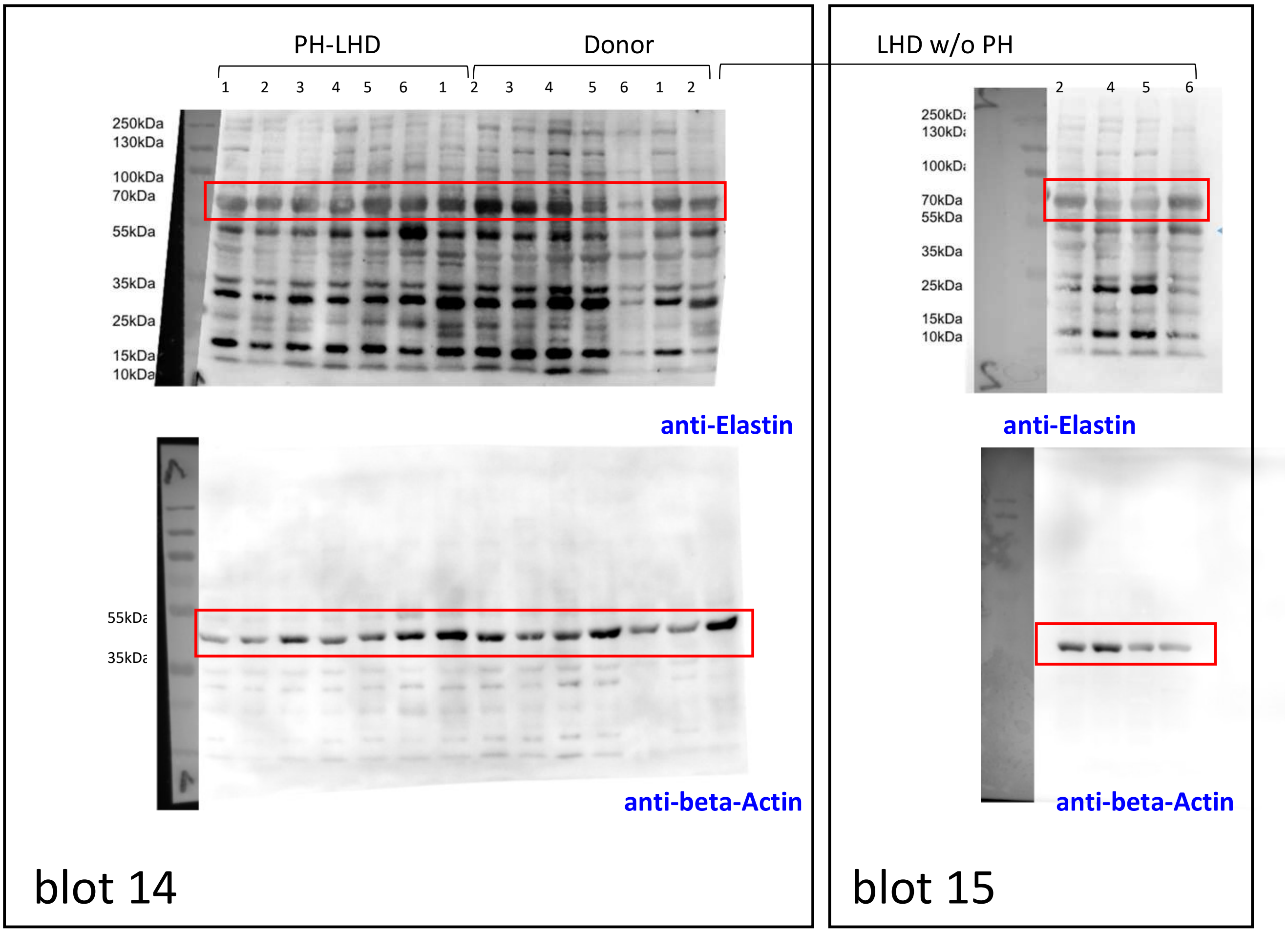

Note: gels were prepared, run and processed in parallel

Figure 12 (continuation).

Original blots corresponding to **Supplementary Figure 7d**

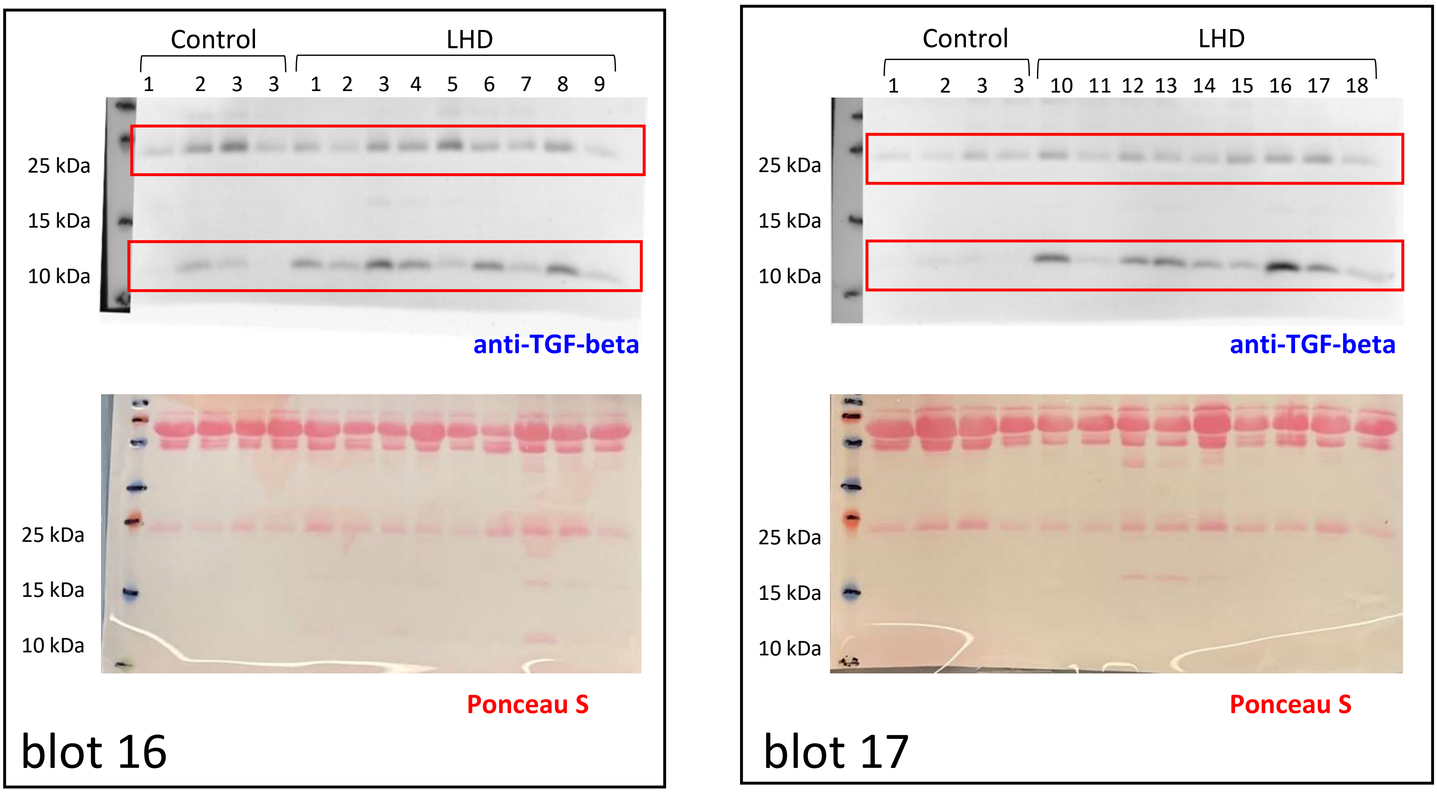

Original blots corresponding to **Supplementary Figure 8d**

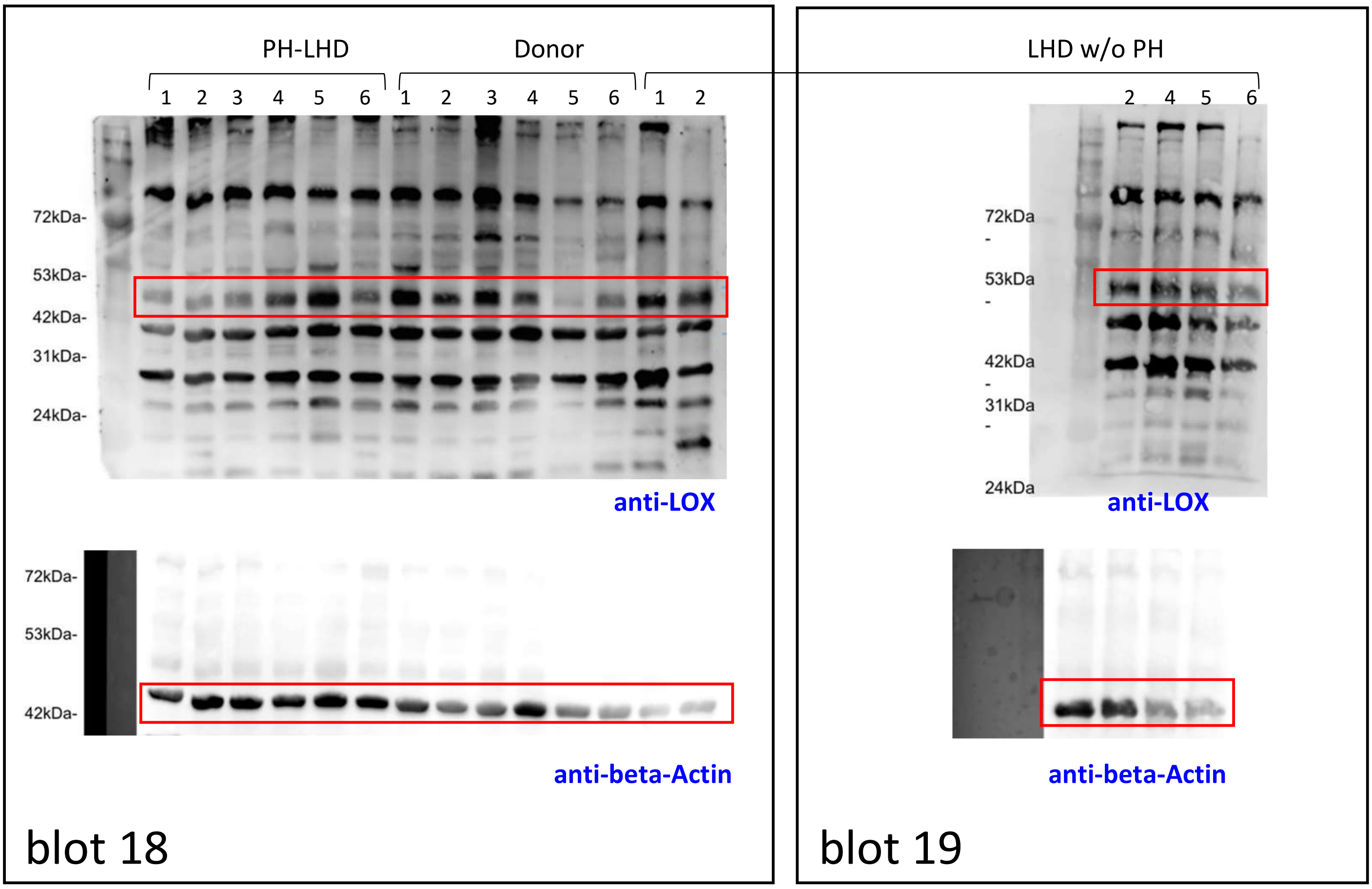

Note: gels were prepared, run and processed in parallel
