# Supplementary material for: Elastin stabilization prevents impaired biomechanics in human pulmonary arteries and pulmonary hypertension in rats with left heart disease
Source: Nat Commun. 2023 Jul 21;14:4416. doi: 10.1038/s41467-023-39934-z (PMC10362055; doi:10.1038/s41467-023-39934-z)
Supplement: Supplementary file 2 — Description of Additional Supplementary File [file 41467_2023_39934_MOESM2_ESM.pdf]

Description of Additional Supplementary File

Supplementary Video Legends:

Supplementary Video 1: 3D reconstruction of elastic fibers in the media of a healthy-heart donor pulmonary artery as detected by autofluorescence. Pseudocolors reflect the vertical depth of fibers in the z-axis (scale).

Supplementary Video 2: 3D reconstruction of elastic fibers in the media of a pulmonary artery from a patient with left heart disease without pulmonary hypertension as detected by autofluorescence. Pseudocolors reflect the vertical depth of fibers in the z-axis (scale).

Supplementary Video 3: 3D reconstruction of elastic fibers in the media of a pulmonary artery from a patient with pulmonary hypertension due to left heart disease as detected by autofluorescence. Pseudocolors reflect the vertical depth of fibers in the z-axis (scale).
