# Supplementary material for: Elastin stabilization prevents impaired biomechanics in human pulmonary arteries and pulmonary hypertension in rats with left heart disease
Source: Nat Commun. 2023 Jul 21;14:4416. doi: 10.1038/s41467-023-39934-z (PMC10362055; doi:10.1038/s41467-023-39934-z)
Supplement: Supplementary file 6 — Reporting Summary [file 41467_2023_39934_MOESM6_ESM.pdf]

nature portfolio

Corresponding author(s): Wolfgang M. Kuebler, Christoph Knosalla

Last updated by author(s): Jul 3, 2023

Reporting Summary

Nature Portfolio wishes to improve the reproducibility of the work that we publish. This form provides structure for consistency and transparency in reporting. For further information on Nature Portfolio policies, see our Editorial Policies and the Editorial Policy Checklist.

Statistics

For all statistical analyses, confirm that the following items are present in the figure legend, table legend, main text, or Methods section.

n/a	Confirmed	
☐	☒	The exact sample size (*n*) for each experimental group/condition, given as a discrete number and unit of measurement
☐	☒	A statement on whether measurements were taken from distinct samples or whether the same sample was measured repeatedly
☐	☒	The statistical test(s) used AND whether they are one- or two-sided<br>*Only common tests should be described solely by name; describe more complex techniques in the Methods section.*
☐	☒	A description of all covariates tested
☐	☒	A description of any assumptions or corrections, such as tests of normality and adjustment for multiple comparisons
☐	☒	A full description of the statistical parameters including central tendency (e.g. means) or other basic estimates (e.g. regression coefficient) AND variation (e.g. standard deviation) or associated estimates of uncertainty (e.g. confidence intervals)
☐	☒	For null hypothesis testing, the test statistic (e.g. *F*, *t*, *r*) with confidence intervals, effect sizes, degrees of freedom and *P* value noted<br>*Give P values as exact values whenever suitable.*
☒	☐	For Bayesian analysis, information on the choice of priors and Markov chain Monte Carlo settings
☒	☐	For hierarchical and complex designs, identification of the appropriate level for tests and full reporting of outcomes
☒	☐	Estimates of effect sizes (e.g. Cohen's *d*, Pearson's *r*), indicating how they were calculated

Our web collection on statistics for biologists contains articles on many of the points above.

Software and code

Policy information about availability of computer code

Data collection	JiveX Demonstration Client software v. 4.7.1 (VISUS), LabChart Pro v. 8 (ADInstruments), Vevo 3100 preclinical imaging system (FUJIFILM VisualSonics), ZEN 2 blue edition v. 1.0 (Zeiss), NIS-Elements AR v. 4.40.00 (Nikon), Leica Application Suite X (LAS X) v. 4.13.0 (Leica Microsystems), bcl2fastq v2.20.0.422 (Illumina), FastQC v. 0.11.5, SnapAndGo (Biostep).
Data analysis	Vevo LAB v. 3.1.1 (FUJIFILM VisualSonics), LabChart Pro v. 8 (ADInstruments), Arivis 4D v. 2.12.5, Imaris 9.2, Fiji-ImageJ-win64 v. 2.11.0, STAR aligner v. 2.5.2a, featureCounts v. 1.5.0-p1, R/Bioconductor environment v. 3.6.1, DESeq2 v.1.24.0, QLUCORE Omics Explorer 3.5, Adobe Photoshop CC 2017, Microsoft Excel 2016, Microsoft Word 2016, OriginPro 8, GraphPad Prism 8, EndNote 20.

For manuscripts utilizing custom algorithms or software that are central to the research but not yet described in published literature, software must be made available to editors and reviewers. We strongly encourage code deposition in a community repository (e.g. GitHub). See the Nature Portfolio guidelines for submitting code & software for further information.

Data

Policy information about availability of data

All manuscripts must include a data availability statement. This statement should provide the following information, where applicable:

- Accession codes, unique identifiers, or web links for publicly available datasets
- A description of any restrictions on data availability
- For clinical datasets or third party data, please ensure that the statement adheres to our policy

Publicly available data of the human genome hg38 version 96 (https://www.ensembl.org/Homo_sapiens/Info/Index) and of the human matrisome (http://matrisomeproject.mit.edu/) were used. RNA-seq data of the human PA transcriptome generated in this study are deposited in Gene Expression Omnibus (GEO) data repository under GSE236251 number (https://www.ncbi.nlm.nih.gov/geo/query/acc.cgi?acc=GSE236251). All other data underlying or supporting the findings in this study are given in the main article and associated files. Source data are provided with this paper.

Human research participants

Policy information about studies involving human research participants and Sex and Gender in Research.

Reporting on sex and gender	The study comprises the analysis of human samples from 33 donors (69.7% male patients), 41 LHD patients without PH (56.1% male patients), 49 LHD with PH (71.4% male patients), and 4 PAH patients (75% males). The study was conducted after informed patient consent was obtained. Sample collection included the use of and access to human biomaterials and phenotyping data in agreement with the recommendations by the German Centre for Cardiovascular Research (Cardiovascular Research (DZHK) for patient for biomaterials and bio-data (https://dzhk.de/uploads/media/DZHK_Nutzungsordnung_engl_05.pdf9). Information on the sex of the patients was taken from the medical records. Results on potential effects of age and sex on PA stiffness are reported in Suppl. Figure 3.
Population characteristics	The detailed patient characteristics are outlined in Suppl. Table 2. As expected in a population of adult patients with advanced heart failure, male patients were predominant.
Recruitment	Human tissue samples were obtained from consecutive patients undergoing heart, lung, or combined heart-lung transplantation.
Ethics oversight	Human tissue samples and blood were collected following approval by the Ethics Committee of the Charité-University Medicine Berlin (EA4/035/18 and EA2/043/19) and with the written informed consent of the patients. The study was conducted in accordance with the principles of the Declaration of Helsinki. A DSMB was not involved; participants did not receive compensation.

Note that full information on the approval of the study protocol must also be provided in the manuscript.

Field-specific reporting

Please select the one below that is the best fit for your research. If you are not sure, read the appropriate sections before making your selection.

☒ Life sciences ☐ Behavioural & social sciences ☐ Ecological, evolutionary & environmental sciences

For a reference copy of the document with all sections, see nature.com/documents/nr-reporting-summary-flat.pdf

Life sciences study design

All studies must disclose on these points even when the disclosure is negative.

Sample size	The analysis of human patient PA samples was an exploratory study that aimed to identify PA stiffening in PH-LHD. Therefore, in screening for differences in PA biomechanics every study group contained more then 20 samples. Other quantitative experiments on human samples included at least 5 biologically independent samples per group. In animal studies, sample size was approximated and derived from extensive publications using the AoB model and based on an a priori power analysis with an anticipated effect size, anticipated standard deviation, p=0.05, and the power of 80%. The average experimental values for RVSP, RVw/Bw, PA wall thickness, and mast cell number in lung in the AoB model vs. sham obtained in our study are in agreement with previous publications. ref 61, 66.
Data exclusions	All obtained data on human patients (patient samples) were included. The exclusion criteria in animal study was improper placement of the clip (detected by echocardiography and/or post-mortem) in the AoB group.
Replication	All experiments and analyses were replicated in multiple biologically independent samples and/or animals as indicated in the respective figure legends. Animal experiments were independently conducted for each subject rat, and all results were pulled.
Randomization	Randomization of rats was performed for selection for surgery and treatment groups among males generated from the same litters.
Blinding	Wherever possible, samples were analyzed in a blinded manner by assigning serial sample numbers for patient samples and/or animals.

Blinding	Experimentators were blinded on group allocation during biomechanical testing of PA samples, RNA-sequencing, image acquisition and processing for histology and immunohistology, CT, TEM, confocal and SHG microscopy. Western blotting experiments were not blinded as samples were loaded by groups. Experimentators could not be blinded during data collection in the animal model, as the placed clip is visible by echocardiography and during cardiac catheterization; yet experimentators were blinded to group assignment for treatment and subsequent analyses were done in a blinded fashion.

Behavioural & social sciences study design

All studies must disclose on these points even when the disclosure is negative.

Study description	*Briefly describe the study type including whether data are quantitative, qualitative, or mixed-methods (e.g. qualitative cross-sectional, quantitative experimental, mixed-methods case study).*
Research sample	*State the research sample (e.g. Harvard university undergraduates, villagers in rural India) and provide relevant demographic information (e.g. age, sex) and indicate whether the sample is representative. Provide a rationale for the study sample chosen. For studies involving existing datasets, please describe the dataset and source.*
Sampling strategy	*Describe the sampling procedure (e.g. random, snowball, stratified, convenience). Describe the statistical methods that were used to predetermine sample size OR if no sample-size calculation was performed, describe how sample sizes were chosen and provide a rationale for why these sample sizes are sufficient. For qualitative data, please indicate whether data saturation was considered, and what criteria were used to decide that no further sampling was needed.*
Data collection	*Provide details about the data collection procedure, including the instruments or devices used to record the data (e.g. pen and paper, computer, eye tracker, video or audio equipment) whether anyone was present besides the participant(s) and the researcher, and whether the researcher was blind to experimental condition and/or the study hypothesis during data collection.*
Timing	*Indicate the start and stop dates of data collection. If there is a gap between collection periods, state the dates for each sample cohort.*
Data exclusions	*If no data were excluded from the analyses, state so OR if data were excluded, provide the exact number of exclusions and the rationale behind them, indicating whether exclusion criteria were pre-established.*
Non-participation	*State how many participants dropped out/declined participation and the reason(s) given OR provide response rate OR state that no participants dropped out/declined participation.*
Randomization	*If participants were not allocated into experimental groups, state so OR describe how participants were allocated to groups, and if allocation was not random, describe how covariates were controlled.*

Ecological, evolutionary & environmental sciences study design

All studies must disclose on these points even when the disclosure is negative.

Study description	*Briefly describe the study. For quantitative data include treatment factors and interactions, design structure (e.g. factorial, nested, hierarchical), nature and number of experimental units and replicates.*
Research sample	*Describe the research sample (e.g. a group of tagged Passer domesticus, all Stenocereus thurberi within Organ Pipe Cactus National Monument), and provide a rationale for the sample choice. When relevant, describe the organism taxa, source, sex, age range and any manipulations. State what population the sample is meant to represent when applicable. For studies involving existing datasets, describe the data and its source.*
Sampling strategy	*Note the sampling procedure. Describe the statistical methods that were used to predetermine sample size OR if no sample-size calculation was performed, describe how sample sizes were chosen and provide a rationale for why these sample sizes are sufficient.*
Data collection	*Describe the data collection procedure, including who recorded the data and how.*
Timing and spatial scale	*Indicate the start and stop dates of data collection, noting the frequency and periodicity of sampling and providing a rationale for these choices. If there is a gap between collection periods, state the dates for each sample cohort. Specify the spatial scale from which the data are taken*
Data exclusions	*If no data were excluded from the analyses, state so OR if data were excluded, describe the exclusions and the rationale behind them, indicating whether exclusion criteria were pre-established.*
Reproducibility	*Describe the measures taken to verify the reproducibility of experimental findings. For each experiment, note whether any attempts to repeat the experiment failed OR state that all attempts to repeat the experiment were successful.*
Randomization	*Describe how samples/organisms/participants were allocated into groups. If allocation was not random, describe how covariates were controlled. If this is not relevant to your study, explain why.*

Blinding | Describe the extent of blinding used during data acquisition and analysis. If blinding was not possible, describe why OR explain why blinding was not relevant to your study.

Did the study involve field work? ☐ Yes ☐ No

Field work, collection and transport

Field conditions	Describe the study conditions for field work, providing relevant parameters (e.g. temperature, rainfall).
Location	State the location of the sampling or experiment, providing relevant parameters (e.g. latitude and longitude, elevation, water depth).
Access & import/export	Describe the efforts you have made to access habitats and to collect and import/export your samples in a responsible manner and in compliance with local, national and international laws, noting any permits that were obtained (give the name of the issuing authority, the date of issue, and any identifying information).
Disturbance	Describe any disturbance caused by the study and how it was minimized.

Reporting for specific materials, systems and methods

We require information from authors about some types of materials, experimental systems and methods used in many studies. Here, indicate whether each material, system or method listed is relevant to your study. If you are not sure if a list item applies to your research, read the appropriate section before selecting a response.

Materials & experimental systems

n/a	Involved in the study	
☐	☒	Antibodies
☒	☐	Eukaryotic cell lines
☒	☐	Palaeontology and archaeology
☐	☒	Animals and other organisms
☒	☐	Clinical data
☒	☐	Dual use research of concern

Methods

n/a	Involved in the study	
☒	☐	ChIP-seq
☒	☐	Flow cytometry
☒	☐	MRI-based neuroimaging

Antibodies

Antibodies used

Rabbit polyclonal anti-LOX (Thermo Fisher, PA1-46020), rabbit polyclonal anti-AGE (Abcam, ab23722), goat anti-rabbit Alexa Fluor 568 (Thermo Fisher, A-11036), rabbit polyclonal anti-CD68 (AbBiotec, 250594), rabbit monoclonal anti-CD68 (Abcam, ab227458, clone EPR20545), rabbit polyclonal anti-McT (Santa Cruz Biotechnology, sc-32889), rabbit polyclonal anti-α-Elastin (Abcam, ab21607), mouse monoclonal anti-Fibrillin-1 (Abcam, ab124334, clone 3H6), rabbit monoclonal anti-Collagen-I (Abcam, ab138492, clone EPR7785), rabbit monoclonal anti-collagen V (Abcam, ab275881), mouse monoclonal anti-IL-6 (Abcam, ab9324, clone 1.2-2B11-2G10), rabbit polyclonal anti-IFN-γ (Invitrogen, PA5-95560), mouse monoclonal anti-MMP2 (Invitrogen, 436000, clone 101), rabbit monoclonal anti-MMP9 (Invitrogen, MA5-32705, clone JA80-73), rabbit polyclonal anti-MMP12 (Abcam, ab128030), rabbit polyclonal anti-MMP13 (Invitrogen, PA5-16566), rabbit polyclonal anti-mast cell tryptase (Santa Cruz Biotechnology, sc-32889), rabbit monoclonal anti-TGF-β1 (Abcam, ab179695, clone EPR18163), horseradish peroxidase (HRP)-conjugated mouse monoclonal anti-β-Actin (Abcam, ab3280-500, clone mAbcam 8226), mouse monoclonal anti-GAPDH (Abcam, ab8245, clone 6C5), goat anti-rabbit HRP (sc-2004, Santa Cruz), and goat anti-mouse HRP (sc-2031, Santa Cruz).

Validation

Rabbit polyclonal anti-LOX (Thermo Fisher, PA1-46020; validated by manufacturer to react with human and rat protein for use in WB, IHC, IF), rabbit polyclonal anti-AGE (Abcam, ab23722, species independent reactivity; validated by manufacturer for use in IF), goat anti-rabbit Alexa Fluor 568 (Thermo Fisher, A-11036), rabbit polyclonal anti-CD68 (AbBiotec, 250594; validated by manufacturer to react with rat protein for use in WB, IHC), rabbit monoclonal anti-CD68 (Abcam, ab227458, clone EPR20545; validated by manufacturer to react with human protein for use in WB, IHC), rabbit polyclonal anti-McT (Santa Cruz Biotechnology, sc-32889; validated by manufacturer to react with human and rat protein for use in WB, IHC), rabbit polyclonal anti-α-Elastin (Abcam, ab21607; validated by manufacturer to react with human protein for use in WB, IHC), mouse monoclonal anti-Fibrillin-1 (Abcam, ab124334, clone 3H6; validated by manufacturer to react with human protein for use in WB, IHC), rabbit monoclonal anti-Collagen-I (Abcam, ab138492, clone EPR7785; validated by manufacturer to react with human protein for use in WB and IHC, and by Xia et al. in rat 117), rabbit monoclonal anti-collagen V (Abcam, ab275881, clone EPR23762-54; validated by manufacturer to react with human and rat protein for use in WB), mouse monoclonal anti-IL-6 (Abcam, ab9324, clone 1.2-2B11-2G10; validated by manufacturer to react with human and rat protein for use in WB), rabbit polyclonal anti-IFN-γ (Invitrogen, PA5-95560; validated by manufacturer to react with human and rat protein for use in WB, IHC), mouse monoclonal anti-MMP2 (Invitrogen, 436000, clone 101; validated by manufacturer to react with human and rat protein for use in WB, IHC), rabbit monoclonal anti-MMP9 (Invitrogen, MA5-32705, clone JA80-73; validated by manufacturer to react with human and rat protein for use in WB, IHC, IF), rabbit polyclonal anti-MMP12 (Abcam, ab128030; validated by manufacturer to react with human protein for use in IHC), rabbit polyclonal anti-MMP13 (Invitrogen,

PA5-16566; validated by manufacturer to react with human and rat protein for use in WB, IHC), rabbit polyclonal anti-mast cell tryptase (Santa Cruz Biotechnology, sc-32889; validated by manufacturer to react with human and rat protein for use in WB, IHC), rabbit monoclonal anti-TGF-β1 (Abcam, ab179695, clone EPR18163; validated by manufacturer to react with human and rat protein for use in WB), horseradish peroxidase (HRP)-conjugated mouse monoclonal anti-β-Actin (Abcam, ab3280-500, clone mAbcam 8226; validated by manufacturer to react with human and rat protein for use in WB), mouse monoclonal anti-GAPDH (Abcam, ab8245, clone 6C5; validated by manufacturer to react with human and rat protein for use in WB), goat anti-rabbit HRP (sc-2004, Santa Cruz), and goat anti-mouse HRP (sc-2031, Santa Cruz).

Animals and other research organisms

Policy information about studies involving animals; ARRIVE guidelines recommended for reporting animal research, and Sex and Gender in Research

Laboratory animals	Sprague Dawley Rat, 5 weeks old (app. 100g bw) at the beginning of the experiment
Wild animals	No wild animals were used in the study
Reporting on sex	In the current study male rats were used. Terminal heart failure is more prevalent in the male population, and in our analyses of human PA samples, we also found PA stiffening to be more pronounced in males as compared to female patients.
Field-collected samples	No field collected samples were used in the study
Ethics oversight	All animal procedures were approved by the local governmental animal care and use committee (Landesamt für Gesundheit und Soziales (LaGeSO), Berlin) under protocol number G0030/18. All experiments were performed in accordance with the ARRIVE guidelines and the "Guide for the Care and Use of Laboratory Animals" (Institute of Laboratory Animal Resources, 8th edition 2011).

Note that full information on the approval of the study protocol must also be provided in the manuscript.
